# Non-Redfield, nutrient synergy and flexible internal elemental stoichiometry in a marine bacterium

**DOI:** 10.1093/femsec/fix059

**Published:** 2017-05-09

**Authors:** Kathleen Trautwein, Christoph Feenders, Reiner Hulsch, Hanna S. Ruppersberg, Annemieke Strijkstra, Mirjam Kant, Jannes Vagts, Daniel Wünsch, Bernhard Michalke, Michael Maczka, Stefan Schulz, Helmut Hillebrand, Bernd Blasius, Ralf Rabus

**Affiliations:** 1 General and Molecular Microbiology, Institute for Chemistry and Biology of the Marine Environment (ICBM), University Oldenburg, Oldenburg 26111, Germany; 2 Mathematical Modelling, Institute for Chemistry and Biology of the Marine Environment (ICBM), University Oldenburg, Oldenburg 26111, Germany; 3 Research Unit Analytical Biogeochemistry, HelmholtzZentrum München, Neuherberg 85764, Germany; 4 Institute for Organic Chemistry, Technische Universität Carolo-Wilhelmina, Braunschweig 38106, Germany; 5 Department of Planktology, Institute for Chemistry and Biology of the Marine Environment (ICBM), Carl von Ossietzky University of Oldenburg, Oldenburg 26111, Germany

**Keywords:** *Phaeobacter inhibens* DSM 17395, growth physiology, N:P ratio, Redfield, Liebig limitation, ecological stoichiometry

## Abstract

The stoichiometric constraints of algal growth are well understood, whereas there is less knowledge for heterotrophic bacterioplankton. Growth of the marine bacterium *Phaeobacter inhibens* DSM 17395, belonging to the globally distributed *Roseobacter* group, was studied across a wide concentration range of NH_4_^+^ and PO_4_^3−^. The unique dataset covers 415 different concentration pairs, corresponding to 207 different molar N:P ratios (from 10^−2^ to 10^5^). Maximal growth (by growth rate and biomass yield) was observed within a restricted concentration range at N:P ratios (∼50−120) markedly above Redfield. Experimentally determined growth parameters deviated to a large part from model predictions based on Liebig's law of the minimum, thus implicating synergistic co-limitation due to biochemical dependence of resources. Internal elemental ratios of *P. inhibens* varied with external nutrient supply within physiological constraints, thus adding to the growing evidence that aquatic bacteria can be flexible in their internal elemental composition. Taken together, the findings reported here revealed that *P. inhibens* is well adapted to fluctuating availability of inorganic N and P, expected to occur in its natural habitat (e.g. colonized algae, coastal areas). Moreover, this study suggests that elemental variability in bacterioplankton needs to be considered in the ecological stoichiometry of the oceans.

## Teaser

*Phaeobacter inhibens* DSM 17395, member of the marine *Roseobacter* group, grows optimally at N:P supply ratios >16, exhibits phytoplankton-like flexible internal elemental stoichiometry and different nutrients control growth synergistically.

## INTRODUCTION

Activity and productivity of microorganisms in marine ecosystems are largely controlled by the availability of the macronutrients nitrogen (N) and phosphorous (P). Most marine organisms use N and P from inorganic (e.g. NO_3_^−^, NH_4_^+^, PO_4_^3−^) as well as organic sources (e.g. proteins, amino acids, nucleic acids, nucleotides, phospholipids and other cell envelope components). Some marine microorganisms (in particular cyanobacteria) transitorily deposit N and P within intracellular storage compounds, e.g. the organic N-rich polymer cyanophycin or inorganic polyphosphates (e.g. Allen 1984; Brock *et al.*^2012^; Burnat, Herrero and Flores 2014). Across different marine systems, e.g. from nutrient-rich estuaries, coastal and upwelling regions to oligotrophic open ocean water bodies, the availability of both macronutrients varies strongly. Further variations emerge from spreading oxygen minimum zones and the associated N loss (Franz *et al.*^2012^; Tsementzi *et al.*^2016^). In the environment, concentrations of total N and P range approximately from 6 to 200 μM and 0.03 to 20 μM, respectively, and molar N:P ratios vary from ∼5 to 310 with an average of 37 (Downing 1997).

Despite this huge variability in nutrient ratios (Hecky and Kilham 1988), oceanographers noticed relatively constant N:P stoichiometry of the particulate matter (seston) in the open ocean (Redfield 1934, 1958). This has been related to the ability of phytoplankton to stabilize the oceanic N:P ratio through nitrogen fixation (Lenton and Klausmeier 2007). Following the same rationale, the canonical Redfield ratio (molar C:N:P of 106:16:1) was suggested to be a global attractor of elemental composition achieved by phytoplankton at growth rates close to μ_max_ (Goldman, McCarthy and Peavey 1979) or through conserved homeostatic relationships between macromolecules (Loladze and Elser 2011). Empirical evidence suggests, however, that phytoplankton can be highly flexible in cellular N:P ratios, reflecting the strongly varying availability of both elements in nature (Guildford and Hecky 2000). This high flexibility is not only observed at the level of entire communities, but even within single species grown under different nutrient supply ratios (Hillebrand *et al.*^2013^). Resolving this discrepancy, Klausmeier *et al.* (2004) modeled optimal N:P ratios for phytoplankton during exponential growth and competitive equilibrium by differentially addressing the cellular machinery for uptake (N-rich proteins) and assembly (P-rich ribosomes). This and other studies indicated that optimal N:P ratios differ between species with the Redfield ratio representing only the median across environmental conditions and phylogenies (Quigg *et al.*^2003^; Klausmeier *et al.*^2004^; Klausmeier, Litchman and Levin 2004; Hillebrand *et al.*^2013^). At low nutrient concentrations, the external N:P ratio determines the elemental composition of phytoplankton (Sterner and Elsner 2002), whereas at high nutrient concentrations, optimal nutrient uptake results in optimal internal N:P stoichiometry independent of the external N:P supply ratio (Klausmeier *et al.*^2004^).

Generally, heterotrophic bacteria compete with phytoplankton for available sources of N and P (e.g. Kirchman 1994; Jørgensen, Kroer and Coffin 1994). Bacteria take up a large proportion of inorganic nutrients (Kirchman 1994) and can outcompete phytoplankton when nutrients are scarce (Currie 1990; Thingstad, Skjoldal and Bone 1993; Joint *et al.*^2002^). Moreover, the presence of bacteria can alter the relative availability of nutrients for autotrophs, and thus shift nutrient limitation (and internal N:P ratios) for phytoplankton (Danger *et al.*^2007^). The uptake and recycling of elements by bacteria is thus a cornerstone of aquatic biogeochemistry (Cotner and Biddanda 2002). Makino *et al.* (2003) paraphrased the central question by asking whether heterotrophic bacteria are more like plants or animals, i.e. whether they show flexibility in elemental composition (as most autotrophs) or not (as most metazoan animals). Heterotrophs are in general significantly more inflexible (homeostatic) than autotrophs (at least with respect to N:P ratios), but they also deviate from homeostasis to variable degrees (Persson *et al.*^2010^). *Escherichia coli* was reported to be rather inflexible (Makino *et al.*^2003^), whereas bacterial communities (Chrzanowski *et al.*^1996^; Vrede *et al.*^2002^; Godwin and Cotner 2014) and single strains (Chrzanowski and Grover 2008; Chan *et al.*^2012^; Godwin and Cotner 2015a,b) exhibited variable internal stoichiometry if nutrient supply varied. Given the central role of bacteria in elemental cycles, the question of how bacterial growth and nutrient incorporation responds to different supply rates and ratios of inorganic nutrients remains a central question for marine ecosystems ecology.

Roseobacters constitute a metabolically diverse group within the alphaproteobacterial *Rhodobacterales* and can account for ∼20% of coastal and ∼15% of mixed-layer ocean bacterioplankton communities. They inhabit coastal and open oceans, sea ice and the sea floor and occur in the planktonic state as well as associated to particles (Buchan, González and Moran 2005; Wagner-Döbler and Biebl 2006). Roseobacters contribute to the recycling of seasonal biomass peaks generated during phytoplankton blooms (Teeling *et al.*^2012^, ^2016^; Luo and Moran 2014; Buchan *et al.*^2014^) and possess a highly adaptive potential towards habitat changes (Luo *et al.*^2014^). *Phaeobacter inhibens* DSM 17395 is a nutritionally versatile representative of roseobacters (Thole *et al.*^2012^; Drüppel *et al.*^2014^; Wiegmann *et al.*^2014^), appears to preferentially interact with biotic (e.g. algae, higher eukaryotes) and abiotic surfaces (e.g. Seyedsayamdost *et al.*^2011^; Gram *et al.*^2015^).

**Figure 1.**
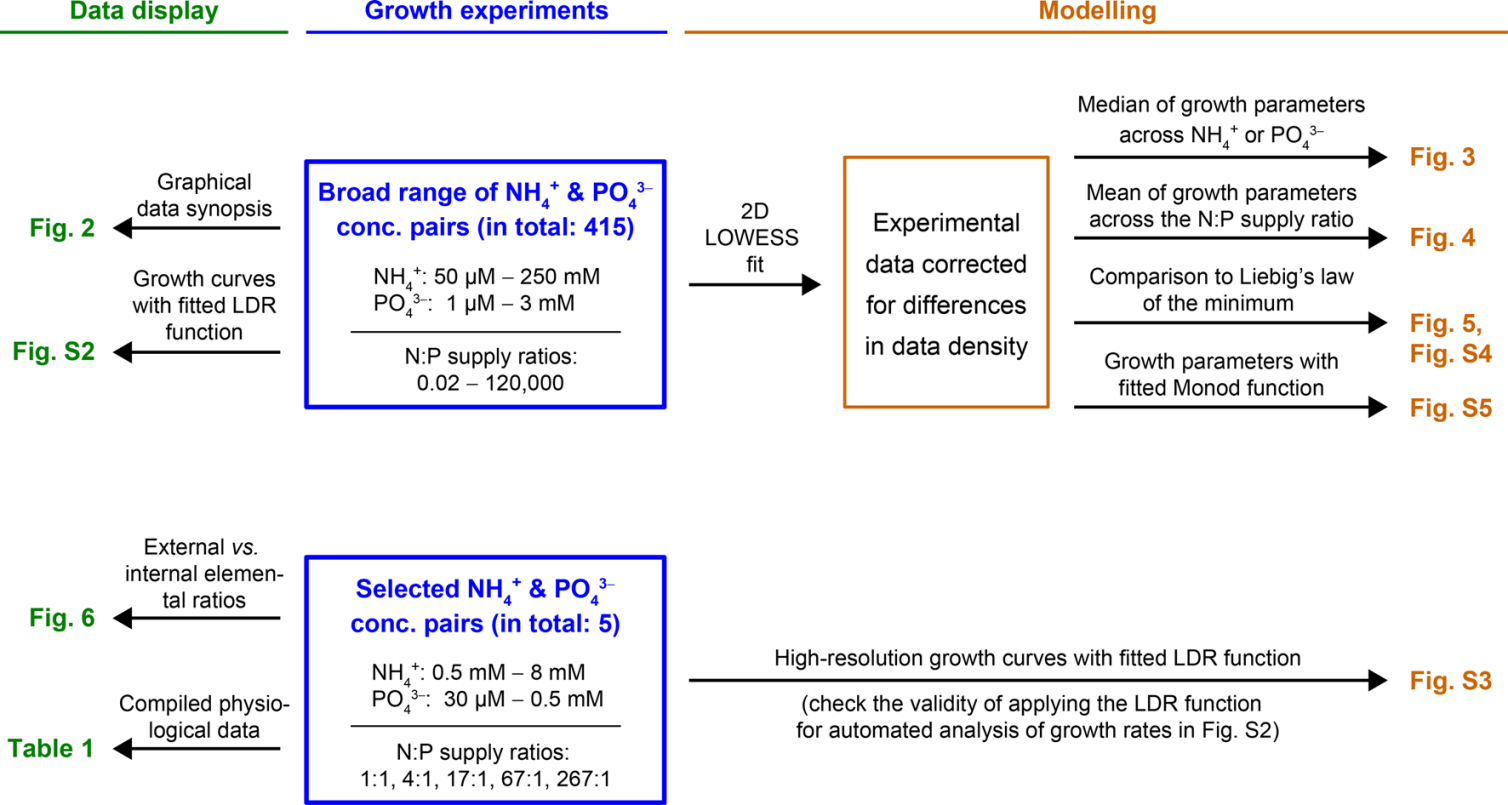
Conceptual design highlighting the interconnections of physiological and modeling approaches, as applied in this study.

*Phaeobacter inhibens* DSM 17395 (and roseobacters in general) dwells in marine systems with differing and/or fluctuating availabilities of nitrogen and phosphorous. This study combines comprehensive experiments on growth physiology with mathematical modeling (see Fig. 1 for conceptual framework) to investigate the nutritional plasticity of *P. inhibens* under widely varying concentrations of ammonium (NH_4_^+^) and phosphate (PO_4_^3−^) against a constant background of glucose as sole source of carbon and energy. We assessed the impact of varying external N:P supply ratios on growth of *P. inhibens* by determining achieved biomass yields (reflected by optical density and cellular dry weight) and growth rates. We found that (i) maximal values for assessed growth parameters were achieved at external N:P ratios **>**16; (ii) growth was synergistically controlled by more than just one nutrient (C, N, P), suspending effectiveness of Liebig's law of the minimum; and that (iii) internal elemental ratios (N:P, C:N, C:P) were flexible. Together, the here reported findings broaden our current perception on the potential of marine bacterioplankton to influence the cycling of macronutrients.

## MATERIALS AND METHODS

### Strain, media, cultivation and harvesting of cells

*Phaeobacter inhibens* DSM 17395 was obtained from the Deutsche Sammlung von Mikroorganismen und Zellkulturen GmbH (DSMZ, Braunschweig, Germany) and since then maintained in our laboratory. Only chemicals of analytical grade and membrane-purified water were used. All cultures were incubated on a rotary shaker (100 rpm) at 28°C in the dark, and growth was monitored by measuring the optical density (OD) at 600 nm (UVmini-1240; Shimadzu, Duisburg, Germany). For purity control, each culture was inspected by light microscopy and spreading on agar plates containing marine broth (MB) medium.

Each individual growth experiment was started according to a defined procedure (Fig. S1, Supporting Information) from a glycerol stock (stored at −80°C) beforehand prepared from cultures of *P. inhibens* grown in MB medium (Zech *et al.*^2009^). Stock cultures were revived using mineral medium (Zech *et al.*^2009^) supplemented with 11 mM glucose, 2 mM ammonium (provided as NH_4_Cl), 0.03 mM phosphate (provided as KH_2_PO_4_) and trace elements. Each nutrient was added from sterile stock solutions to the autoclaved mineral medium. To eliminate residual glycerol and MB medium, a serial dilution (up to 10^–5^) was prepared with 13.5 mL mineral medium (in 50 mL Erlenmeyer flasks) and a 1.5 mL glycerol stock. This was followed by two successive passages (50 mL culture in 250 mL Erlenmeyer flasks) in the same mineral medium, but with NH_4_^+^ and PO_4_^3−^ adjusted to one of 415 different concentration pairs. Main experiments were conducted in 1 L Erlenmeyer flasks containing 250 mL medium and with at least three biological replicates. All cultures were consistently inoculated with 2% (v/v) of the respective preceding culture when it had reached approximately half-maximal OD (∼0.5 OD_max_). To avoid carryover of any residual PO_4_^3−^ at very low PO_4_^3−^ concentrations (<50 μM), glassware was specifically cleansed by overnight incubation in HCl (1%, v/v), followed by thorough rinsing with membrane-purified water. Growth experiments with 415 different NH_4_^+^ and PO_4_^3−^ concentration pairs were conducted over a period of 5 years by seven different experimenters. Coherent experimental proceedings were ascertained for each experimenter by initial and repeated cultivation at the same NH_4_^+^ (2 mM) and PO_4_^3−^ (30 μM) concentration pair. The dataset compiled in Fig. S2 (Supporting Information) derives from ∼1300 individual cultures and ∼20 000 OD measurements.

Detailed cellular-physiological analyses were performed for five selected NH_4_^+^ and PO_4_^3−^ concentration pairs, corresponding to N:P supply ratios of 267:1, 67:1, 17:1, 4:1 and 1:1 (Fig. S3, Supporting Information). These are above, close to and below the Redfield ratio, respectively. Per supply ratio, high-resolution growth curves were obtained from four parallel cultures (250 mL in 1 L Erlenmeyer flasks) to monitor OD_600_ and absorbance at 398 nm (Abs_398_), as well as consumption of glucose, NH_4_^+^ and PO_4_^3−^. Simultaneously, sufficient cell material was generated for biomass analyses from eight additional parallel cultures per N:P supply ratio, harvested by centrifugation (11 300 × *g*, 20 min, 4°C; Avanti J-25, Beckmann Coulter, Krefeld, Germany) at ∼0.5 OD_max_ and OD_max_. In case of OD_max_, 45 mL of the culture supernatant was immediately filtered (0.2 μm CA; Sartorius, Göttingen, Germany), shock frozen in liquid nitrogen and analyzed within 2 days to quantify the excreted antibiotic tropodithietic acid (TDA). Cell pellets were used to determine the cellular dry weight (CDW) and the elemental composition at ∼0.5 OD_max_ and OD_max_ (see below section ‘Chemical analyses’).

### Standardized calculation of growth rates

**Figure 2.**
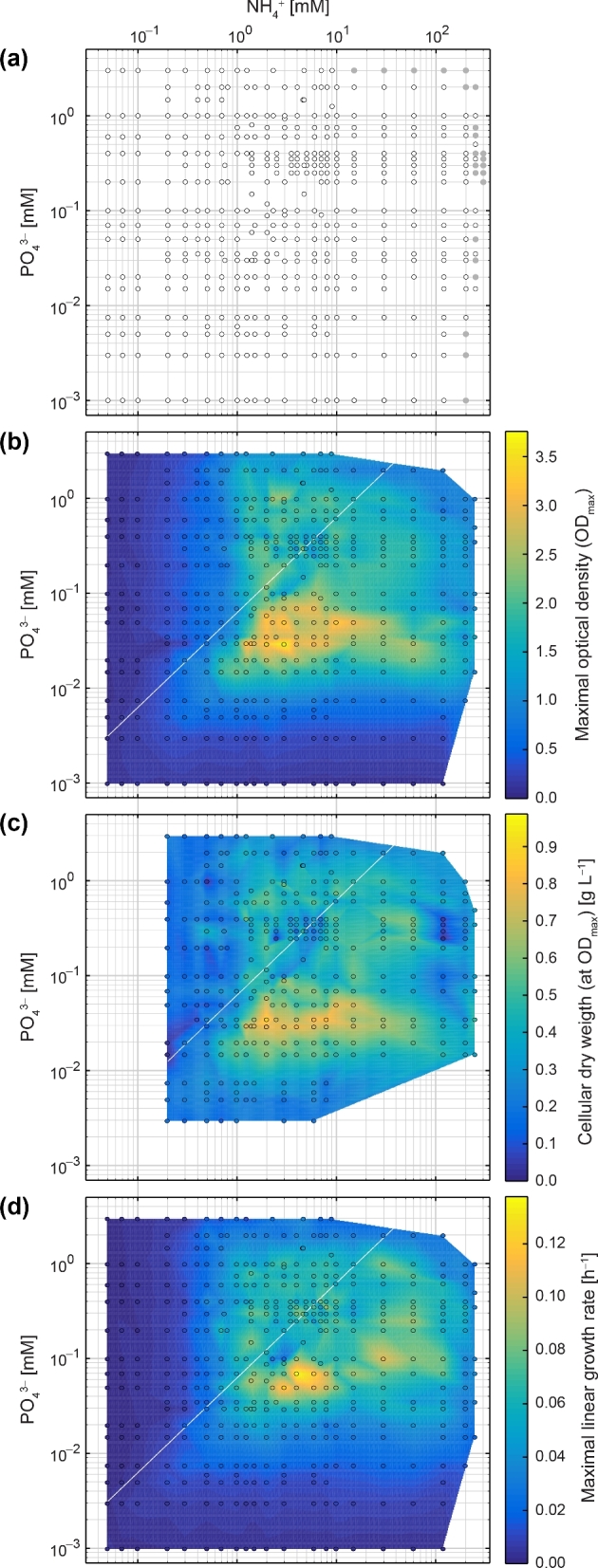
Growth of *P. inhibens* with varying concentrations of NH_4_^+^ (50 μM to 250 mM) and PO_4_^3−^ (1 μM to 3 mM). Glucose (sole source of carbon and energy) was in each case provided at the same initial concentration (11 mM). NH_4_^+^ and PO_4_^3−^ concentrations are plotted in logarithmic scale, growth parameters in colored linear scales. (**a**) Grid display of the analyzed 415 different concentration pairs of NH_4_^+^ and PO_4_^3−^ that correspond to 207 different N:P supply ratios ranging from 10^–2^ to 10^5^. Filled gray circles indicate concentrations where growth was not observed. Colored data maps display the (**b**) maximal optical density (OD_max_) reached upon entry into stationary growth phase, (**c**) the attained cellular dry weight at OD_max_ and (**d**) the calculated maximal growth rate (μ_lin_) during linear growth (representing the major active growth phase) for each concentration pair of NH_4_^+^ and PO_4_^3−^. The diagonal white line represents the canonical Redfield N:P ratio of 16:1. The circles in subfigures b–d represent experimentally determined values and colored areas between these data points were retrieved by linear interpolation. See Fig. 3 for analyzed growth parameters as a function of NH_4_^+^ or PO_4_^3−^ concentration. Corresponding growth curves with fitted logistic dose–response (LDR) function are compiled in Fig. S2 (Supporting Information).

**Figure 3.**
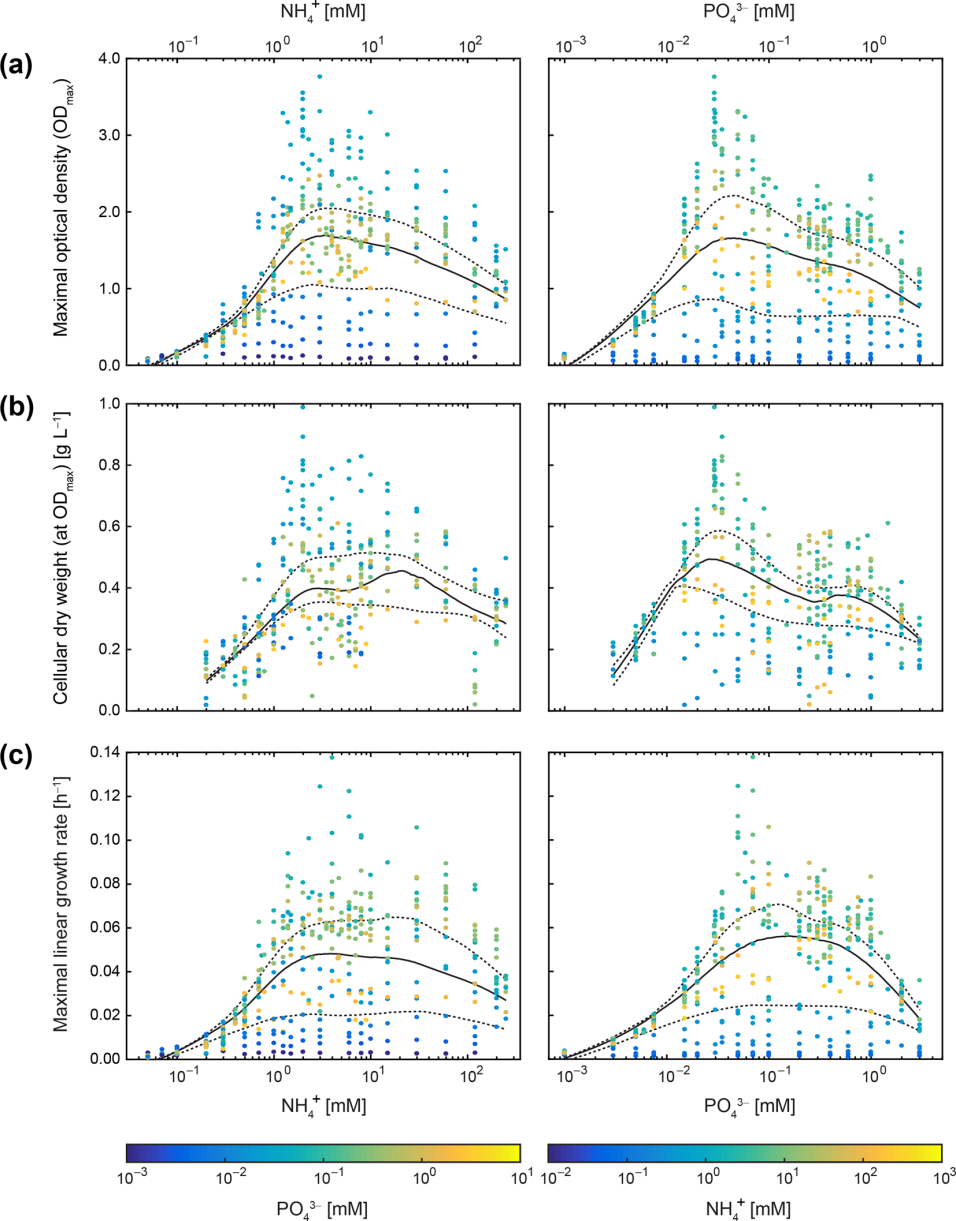
Median of growth parameters across NH_4_^+^ and PO_4_^3−^ concentrations. Semilogarithmic profiles of (**a**) maximal optical density (OD_max_), (**b**) cellular dry weight at OD_max_ and (**c**) maximal linear growth rate (μ_lin_) for *P. inhibens* as a function of NH_4_^+^ or PO_4_^3−^ concentration (see Fig. 2b–d for joint display in color maps). Colors of data points indicate the corresponding NH_4_^+^ or PO_4_^3−^ concentration, respectively. The black solid line displays the calculated median and the dashed ones the 25% and 75% quantiles (based on 2D LOWESS fit to experimental data). A complementary fitting of a Monod function to the experimental data is shown in Fig. S5 (Supporting Information).

**Figure 4.**
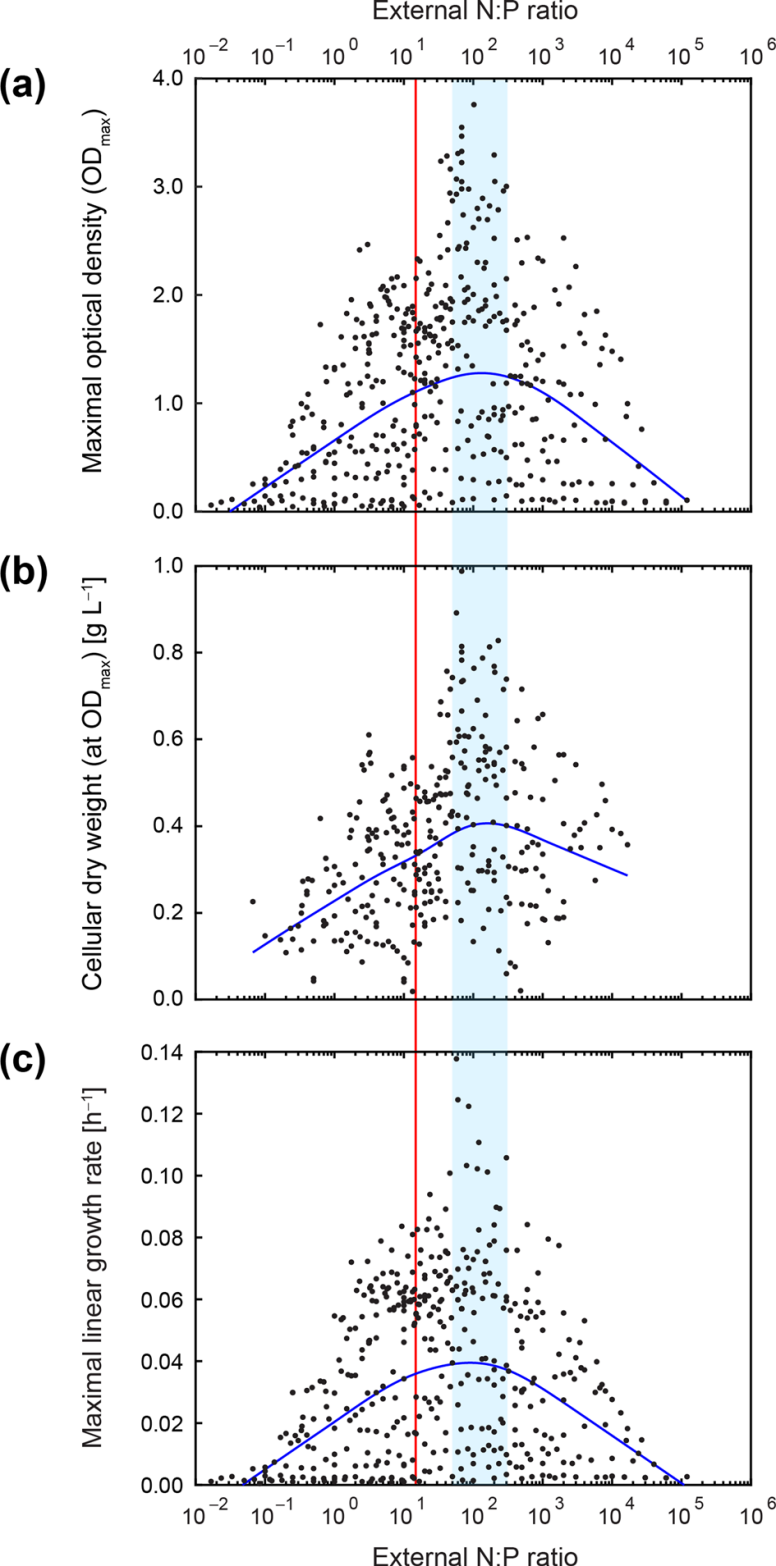
Mean of growth parameters across the N:P supply ratios. Semilogarithmic profiles of (**a**) maximal optical density (OD_max_), (**b**) cellular dry weight at OD_max_ and (**c**) maximal linear growth rate (μ_lin_) for *P. inhibens* as a function of the external N:P ratio. The blue line displays the calculated mean (based on 2D LOWESS fit) for the experimental data (black dots). The vertical red line marks the Redfield N:P ratio of 16:1. The blue shaded area delimits the approximate range of N:P supply ratios (∼50–120), at which the mean of the studied growth parameters was maximal.

**Figure 5.**
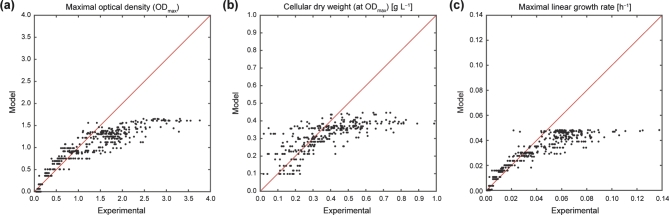
Comparison of growth parameters to Liebig's law of the minimum. Experimental values for *P. inhibens* were compared to modeled ones for (**a**) maximal optical density (OD_max_), (**b**) cellular dry weight at OD_max_ and (**c**) maximal linear growth rate (μ_lin_) across the studied concentration range of NH_4_^+^ and PO_4_^3−^. The diagonal red line represents the exact match of measured values to model prediction for single nutrient-limited growth based on Liebig's law of the minimum. For data points above this line, predicted values were higher than measured, whereas measured values exceeded the model prediction below this line. A more detailed comparison of experimental values with those predicted by Liebig's law of the minimum is shown in Fig. S4 (Supporting Information).

Growth with these widely varying NH_4_^+^ and PO_4_^3−^ concentration pairs (415) was assessed by monitoring OD (Figs 2–5; Fig. S2, Supporting Information). A logistic dose–response (LDR) function (Beckon *et al.*^2008^)

\[
ldr (t) = a + \frac{b}{{1 + {{\left( {\frac{t}{c}} \right)}^d}}}
\]

(*a*: OD directly after inoculation (OD_start_), *b*: difference between OD_max_ and OD_start_, *c*: time when the medium value *a* + 0.5*b* is reached, *d*: controls the shape of the curve) was fitted to experimental OD data, as recently described (Trautwein *et al.*^2016^). Based on this fit, the linear growth rate (μ_lin_) was estimated as the slope of the LDR function at the inflection point (Trautwein *et al.*^2016^). Validity of applying the LDR function to this comprehensive dataset was verified based on high-resolution growth curves determined for five selected NH_4_^+^ and PO_4_^3−^ supply ratios (Table 1; Fig. S3, Supporting Information). For each of the 415 different NH_4_^+^ and PO_4_^3−^ concentration pairs, similarity of biological replicates was confirmed before fitting the LDR function to averaged OD measurements, minimizing the mean square error for all measurements before and at OD_max_ (Fig. S2, Supporting Information). Exponential growth was not included in the comprehensive dataset, as this growth phase was rather short and restricted to early growth, which could not be covered in sufficiently high resolution. Analytical calculations were performed with Maple 18.02 (Maplesoft, Waterloo, Canada); data fitting and numerical calculations were performed with MATLAB R2016a (The MathWorks, Natick, MA, USA).

**Table 1.** Cellular and physiological characteristics of *P. inhibens* during growth with selected supply ratios of NH_4_^+^ and PO_4_^3−^ (for growth curves see Fig. S3, Supporting Information).

Physiological parameters	External N:P supply ratio^a^				
	267 (P-limited)	67 (P-limited)	17 (NP-limited)	4 (N-limited)	1 (N-limited)
OD_max_ (600 nm)	3.24 ± 0.03	3.26 ± 0.04	0.92 ± 0.01	0.61 ± 0.02	0.58 ± 0.04
μ_exp_ [h^−1^]	0.163 ± 0.003	0.148 ± 0.003	0.225 ± 0.006	0.207 ± 0.003	0.250 ± 0.001
μ_lin_ [h^−1^]	0.090 ± 0.002	0.087 ± 0.002	0.040 ± 0.001	0.024 ± 0.001	0.025 ± 0.002
*Y_Glc_* [g dry cells (mol Glc)^−1^]^b^	66.8 ± 1.2	63.3 ± 1.0	46.5 ± 1.2	63.0 ± 0.2	55.1 ± 0.2
${Y_{NH_4^ + }}$ [g dry cells (mol NH^4+^)^−1^]^b^	494 ± 18	493 ± 2	360 ± 12	315 ± 6	296 ± 2
${Y_{PO_4^{3 - }}}$ [g dry cells (mol PO_4_^3−^)^−1^]^b^	23 883^c^	21 185^c^	5393^c^	3178 ± 211	2,763 ± 46
Internal N:P ratio (biomass)^d^	36	43	16	12	13
Internal N:P ratio (biomass)^b^	46	57	19	11	12
Internal C:N ratio (biomass)^d^	18	19	14	11	10
Internal C:N ratio (biomass)^b^	30	26	22	16	17
Internal C:P ratio (biomass)^d^	661	821	233	129	129
Internal C:P ratio (biomass)^b^	1408	1460	420	183	197
TDA (Abs_398nm_) ^b^	0.54 ± 0.01	0.58 ± 0.01	0.04 ± 0.00	0.03 ± 0.00	0.06 ± 0.00
TDA [nM] ^b^	0.19 ± 0.05	0.24 ± 0.09	0	0	0

^a^Selected molar N:P supply ratios with concentrations of NH_4_^+^ and PO_4_^3−^ (in brackets, respectively) in the medium: 267 (8.0 mM, 30 μM), 67 (2.0 mM, 30 μM), 17 (0.5 mM, 30 μM), 4 (0.5 mM, 125 μM) and 1 (0.5 mM, 0.5 mM).

^b^Values at ∼OD_max_ (i.e. at the transition into stationary growth phase). Molar growth yields were calculated according to the following formula:

${Y_{{\rm{nutrient}}}}\ [{\rm{g\ dry\ cells\ }} ( {{\rm{mol\ nutrient}}{)^{-1}}} ] = \frac{{{\rm{CDW\ formed\ }}[ {{\rm{g\ }}{{\rm{L}}^{-1}}} ]}}{{{\rm{Nutrient\ consumed\ }}[ {{\rm{mol\ }}{{\rm{L}}^{-1}}} ]}}$.

^c^Complete consumption of PO_4_^3−^ assumed (initial concentration below limit of quantitation [50 μM]; Ruppersberg *et al.*^2016^).

^d^Values at ∼0.5 OD_max_ during linear growth.

### Data analysis with modeling

For each growth parameter, a 2D locally weighted regression (LOWESS) was computed to compensate for inhomogeneous data density across the studied range of 415 different NH_4_^+^ and PO_4_^3−^ concentration pairs (Cleveland 1979). The resulting surface has been used to calculate the quantiles (25%, 50% and 75%) for each growth parameter across the NH_4_^+^ and PO_4_^3−^ profiles, respectively. The median profile functions ${f_{NH_4^ + }}( N )$ and ${f_{PO_4^{3 - }}}( P )$ (black solid lines in Fig. 3) were combined according to Liebig's law of the minimum, which takes the smaller of the two values for any nutrient combination (*N*, *P*), resulting in a simple predictive model. Model predictions were computed and compared to actual measurements (Fig. 5; Fig. S4, Supporting Information). The LOWESS surface was further used to calculate mean values across the covered range of external N:P ratios (blue line in Fig. 4).

### Chemical analyses

The concentrations of NH_4_^+^ and PO_4_^3−^ in cell-free culture supernatants were determined with photometric assays, employing a microplate reader (MPR) as described in detail by Ruppersberg *et al.* (2016). The colorimetric determination of NH_4_^+^ was based on its reaction with sodium salicylate and sodium hypochlorite. The NH_4_^+^ assays were incubated for 15 min at 37°C in the MPR and then measured at 620 nm. The detection limit for NH_4_^+^ was 14 μM, and the linear range for quantitative determination was 36−200 μM. The colorimetric determination of PO_4_^3−^ was based on its complex formation with ammonium molybdate in the presence of ascorbate and zinc acetate at pH 5. The PO_4_^3−^ assays were incubated for 30 min at 30°C in the MPR and then measured at 620 nm. The detection limit for PO_4_^3−^ was 13 μM and the linear range for quantitative determination was 50 μM to 1 mM.

The concentration of glucose in cell-free culture supernatants was determined by HPLC analysis. The system consisted of an UltiMate 3000 Rapid Separation LC (ThermoFisher Scientific, Germering, Germany) equipped with a Eurokat separation column (8 × 300 mm, 5 μm bead size; Knauer, Berlin, Germany) temperature controlled at 75°C and a refractive index (RI) detector (RI-101; Shodex, Munich, Germany). The eluent was composed of 5 mM H_2_SO_4_ and administered at a flow rate of 1.2 mL min^–1^. The system was controlled by the Chromeleon (version 7.1) software (ThermoFisher Scientific). Calibration was performed with a glucose standard (retention time at 5.6 min) diluted in mineral medium. The linear range for quantitative glucose analysis was from 10 μM to 15 mM.

Cell pellets for analysis of the CDW were washed twice with 50 mM ammonium acetate, then resuspended in 300 μL 50 mM ammonium acetate and transferred into pre-dried and weighed 1.5 mL reaction tubes. The CDW was determined by gravimetric analysis after drying of tubes at 60°C to constant weight. Then, CDW samples were stored at room temperature until used for determination of the elemental composition of cells.

The cellular C, H, N and S content was determined by subjecting CDW samples (1–5 mg) to high temperature oxidation, sequential heat-dependent release from adsorption columns and thermal conductivity detection using a Vario EL cube (Elementar Analysensysteme GmbH, Hanau, Germany) essentially as described before (Zech *et al.*^2013^). The cellular P content was determined according to Schramel (1983) as follows. The samples were properly weighed into quartz vessels and 1 mL HNO_3_ (suprapure, sub-boiling distilled; Merck, Darmstadt, Germany) was added. The vessels were closed and introduced into a pressure digestion system (Seif Aufschlussapparatur; Seif, Unterschleissheim, Germany) for 10 h at 170°C. The resulting clear solution was filled up exactly to 5 mL with ultrapure and filtered H_2_O. An inductively coupled plasma atomic emission spectrometer (ICP-AES Optima 7300 system; Perkin Elmer, Rodgau-Jügesheim, Germany) was used for P and S determination. Samples were introduced into the system by a peristaltic pump (flow rate 0.8 mL min^−1^) connected to a Seaspray nebulizer with a cyclon spray chamber. The measured spectral element line was 177.495 nm for P and 182.034 nm for S. The radio frequency power was set to 1350 W, the plasma gas was supplied at 15 L Ar min^−1^, the auxiliary gas at 0.2 L Ar min^−1^ and the nebulizer gas at 0.6 L Ar min^−1^. Every 10 samples, three blanks and a certified standard (CPI, with Lots 08G043 and G9B079) were measured. Calculations were carried out with a computerized lab-data management system, relating sample measurements to calibration curves, blanks, CPIs and to the initial dry weight of digested samples. The S content of biomass was determined to compare and integrate the data from the two independent methods. Elemental ratios mentioned in this study describe exclusively the molar ratios of C, N or P.

Presence of TDA was estimated spectrophotometrically by the absorbance increase in cell-free culture supernatants at 398 nm (D’Alvise *et al.*^2016^). In addition, TDA was quantified from cell-free culture supernatant (45 mL) as follows: first, the pH of the supernatant was adjusted to 3.0 with 2 M HCl, followed by extraction with 20 mL ethyl acetate, which was repeated two times. Ethyl acetate was removed under vacuum and the precipitate was dissolved in 1 mL acetonitrile, from which 2 μL were injected into an HPLC system equipped with an LTQ XL mass detector operated in ESI-negative mode (both ThermoFisher Scientific). TDA was separated on a Hypersil GOLD C_18_ column (2.1 × 50 mm; ThermoFisher Scientific) using an acetonitrile-water gradient containing 0.25% (v/v) formic acid as the eluent. The gradient started with 5% (v/v) acetonitrile and increased linearly to 95% (v/v) within 4 min and was then held constant for 3 min. TDA concentrations were determined from peak areas following detection by MS/MS with Selected Reaction Monitoring of the *m/z* 166.9 fragment derived from *m/z* 211.1 (TDA). Calibration curves were prepared from purchased TDA (Sigma-Aldrich, St. Louis, USA) dissolved in acetonitrile:water (1:1). The retention time of TDA was 3.9 min, and the linear range for quantitative TDA analysis covered 1 to 25 μg mL^–1^.

## RESULTS

### Highest growth rates and biomass yields at N:P »16

The broad concentration range (NH_4_^+^: 50 μM to 250 mM; PO_4_^3–^: 1 μM to 3 mM) tested in this study comprised 415 different NH_4_^+^ and PO_4_^3–^ concentration pairs (Fig. 2a; Fig. S2, Supporting Information), which represent 207 different N:P supply ratios that ranged from 0.02 to 120 000. For each concentration pair, we recorded OD values in regular intervals (to calculate growth rates) and analyzed biomass yields from determined CDW at OD_max_ (i.e. at the transition of cultures into stationary growth phase).

Across the complete dataset, maximal values for OD_max_ (Fig. 2b) and corresponding CDW (Fig. 2c), as well as for maximal linear growth rates (μ_lin_; Fig. 2d), were observed at N:P supply ratios markedly above Redfield (N:P >16). Average OD_max_ values were 2.9 within a concentration range of ∼1.5−15 mM NH_4_^+^ and ∼30−70 μM PO_4_^3–^, with the maximum (3.8) recorded at 3 mM NH_4_^+^ and 30 μM PO_4_^3–^. Correspondingly, CDW values (average of 774 mg L^–1^) were highest at ∼30 μM PO_4_^3–^ within a similar NH_4_^+^ concentration range (∼1.5–10 mM), with the maximum (988 mg L^–1^) observed at 2.0 mM NH_4_^+^. Biomass yields were considerably lower (average OD of 1.8 and CDW of 452 mg L^–1^) for concentration pairs of 1−60 mM NH_4_^+^ and 15 μM to 1 mM PO_4_^3−^, excluding the here embedded concentration pairs supporting maximal values. Strong growth limitation with an achieved average OD_max_ of 0.42 and CDW of 206 mg L^–1^ prevailed at concentrations <1 mM NH_4_^+^ or <10 μM PO_4_^3−^. At concentrations of >120 mM NH_4_^+^ or >3 mM PO_4_^3−^, growth was not observed. Exponential growth was short and always confined to early growth, not well covered by the experimental data. On average, the exponential growth phase contributed only ∼10% to the total time elapsed until OD_max_ was reached. Since growth was linear during the main active growth phase, μ_lin_ was determined by fitting an LDR function to experimental OD values (Fig. 2d). Values for μ_lin_ were highest (on average 0.100 h^–1^) within a similar concentration range of NH_4_^+^ (2.3−10 mM), but at slightly higher PO_4_^3–^ (50−70 μM) concentrations than observed for OD_max_ and CDW. The maximal value for μ_lin_ (0.138 h^–1^) was observed at 4.0 mM NH_4_^+^ and 70 μM PO_4_^3−^, whereas lowest values (average of 0.012 h^–1^) were again tied to strong resource limitation (<1 mM NH_4_^+^ or <10 μM PO_4_^3–^).

A 2D LOWESS fit was applied to the experimental data shown in Fig. 2, to correct for differences in data density. Within a concentration range of about one order of magnitude, the three growth parameters (Fig. 3) increased as a function of the external NH_4_^+^ or PO_4_^3–^ concentration (up to ∼2–3 mM or ∼20–40 μM, respectively). Above these concentrations, median values were quite stable (except for PO_4_^3–^ in Fig. 3a and b) until they declined with different slopes at the respective higher concentrations tested. The concentration–response profiles thus revealed zones, where growth rate and biomass yields (i) were apparently controlled and limited by the external NH_4_^+^ or PO_4_^3–^ concentration, (ii) became saturated and (iii) were inhibited at higher concentrations (especially in case of PO_4_^3–^). Maximal mean values for growth rate and biomass yields were observed at external N:P supply ratios ranging from ∼50 to 120 (Fig. 4).

Detailed physiological growth experiments were conducted that covered N:P supply ratios from 1 to 267 (Table 1) to assess (i) substrate consumption, (ii) growth efficiency (by molar growth yields) and (iii) the internal elemental stoichiometry of *P. inhibens*. Physiological parameters varied strongly at external N:P ratios ranging from 4 to 67; beyond this range (i.e. at 1 and 267, respectively) they remained essentially unchanged. Molar growth yields were calculated from the formed biomass and associated resource consumption at ∼OD_max_ (Table 1). Obtained values were similar for glucose (*Y_Glc_*), but varied ∼1.7-fold for NH_4_^+^ (${Y_{NH_4^ + }}$) and ∼10-fold for PO_4_^3–^ (${Y_{PO_4^{3 - }}}$) across the studied range of external N:P ratios. Notably, both, ${Y_{NH_4^ + }}$ and ${Y_{PO_4^{3 - }}}$ were maximal at N:P ratios **>**16 (PO_4_^3–^-limited) and then declined with decreasing N:P ratios. The secondary metabolite TDA was produced in detectable amounts only at high NH_4_^+^ concentrations, i.e. at N:P supply ratios of 67 and 267.

### Synergistic interaction of nutrients

Experimental values were plotted against those predicted by Liebig's law of the minimum using the LOWESS fit-derived median profiles for NH_4_^+^ and PO_4_^3−^ (see Fig. 3). The experimental values for the three analyzed growth parameters (Fig. 5; Fig. S4, Supporting Information) diverted markedly above an OD_max_ of ∼1.0, a CDW of ∼0.354 g L^–1^ and a μ_lin_ of ∼0.04 h^–1^ from the diagonal line, which represents the exact match of experimental values with those predicted by Liebig's law of the minimum. Thus, the nutrient limitation model matched the experimental data only at low and strongly growth-limiting concentrations of NH_4_^+^ (<∼1 mM). At higher concentrations, realized growth rates and biomass yields were mostly larger than predicted.

### Flexible internal elemental stoichiometry

**Figure 6.**
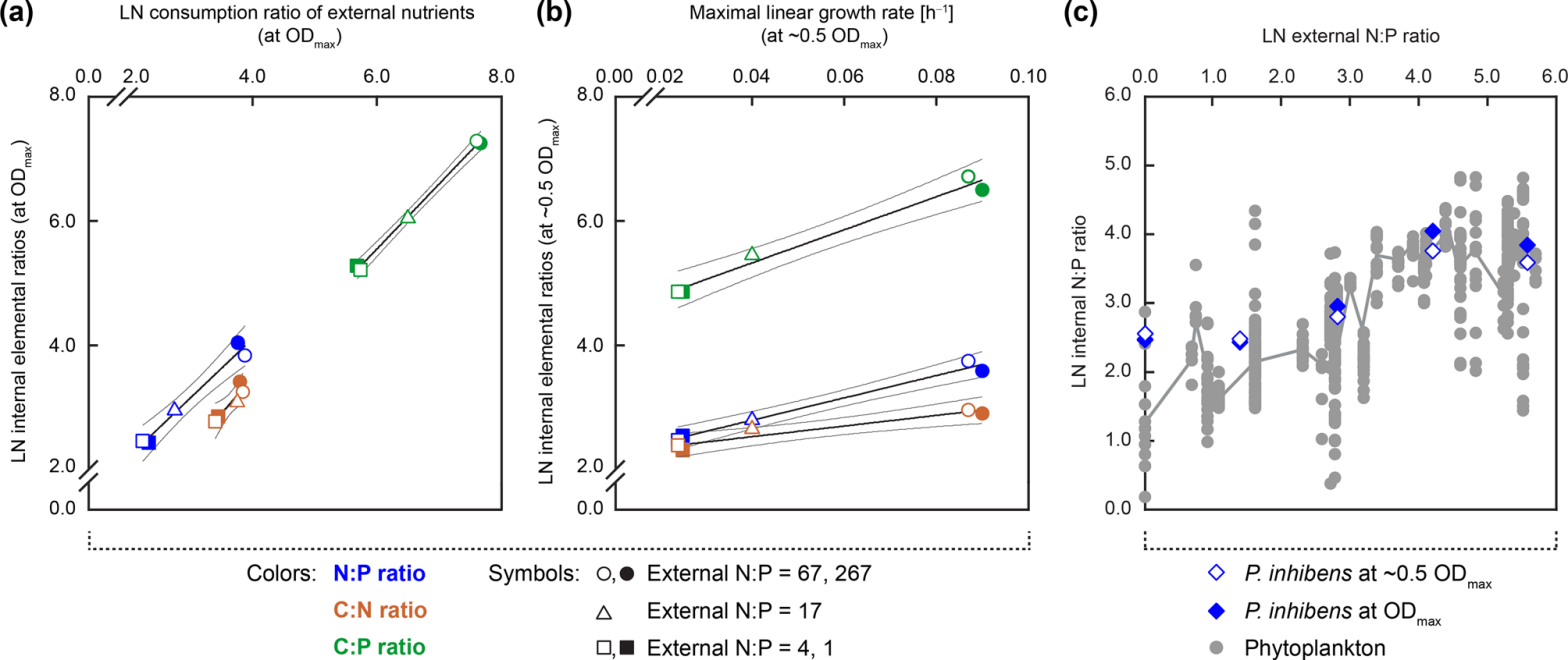
Imprint of external NH_4_^+^ and PO_4_^3−^ supply ratios on internal elemental stoichiometry of *P. inhibens*. (**a**) Internal elemental ratios for C, N, and P as a function of the consumption ratio of glucose, NH_4_^+^ and PO_4_^3−^ (both at OD_max_). (**b**) Relation of maximal linear growth rate (μ_lin_) to internal elemental ratios (both at ∼0.5 OD_max_). Linear growth represented the major active growth phase, whereas exponential growth was mostly very short and confined to early growth. Values for μ_lin_ were largest during P-limitation, whereas the exponential growth rate (μ_exp_; Table 1) was higher under P-excess (as to be expected from the growth rate hypothesis; Elser *et al.*^2000^). (**c**) Internal N:P ratios of *P. inhibens* (blue) and phytoplankton (gray) as a function of external N:P ratios. Phytoplankton data (from marine and freshwater species) were compiled from a meta-analysis of phytoplankton stoichiometry and growth rate (Hillebrand *et al.*^2013^). The gray line displays the median across the phytoplankton data. LN represents the natural logarithm.

Internal elemental ratios (N:P, C:N and C:P) of *P. inhibens* positively correlated with the consumption ratios of glucose, NH_4_^+^ and PO_4_^3–^ (at OD_max_) for external N:P ratios from 4 to 67 (Fig. 6a). Within this range, internal elemental ratios varied ∼5-fold for the N:P, ∼1.6-fold for the C:N and ∼8-fold for the C:P ratio (for values at OD_max_; Table 1), suggesting that the internal C:P stoichiometry is more flexible than C:N. For external N:P ratios of 4 and 67, μ_lin_ positively correlated with internal elemental ratios during linear growth (at ∼0.5 OD_max_) (Fig. 6b). Within this range, values for μ_lin_ were ∼3.6-fold higher during P-limited as compared to N-limited growth (Table 1). In contrast, exponential growth rate (μ_exp_; Table 1) was highest under conditions of P-excess, agreeing with the growth rate hypothesis, which suggests that low internal N:P ratios are characterized by P-rich rRNA and high μ (Elser *et al.*^2000^; Sterner *et al.*^2008^). Here, external N:P ratios apparently only positively affected the exponential growth rate (μ_exp_; Table 1), which was short and confined to early growth. Below (i.e. 1) or above (i.e. 267), the before mentioned range of external N:P ratios, internal elemental composition and growth rates remained unchanged.

The range of supplied external N:P ratios (1 to 267) in this experiment matched the range of 55 phytoplankton studies (both freshwater and marine) compiled in the meta-analysis by Hillebrand *et al.* (2013) (Fig. 6c). Phytoplankton comprised diatoms, dinoflagellates, chlorophytes, prymnesiophytes and cyanobacteria (see Hillebrand *et al.*^2013^ for details on data retrieval). Comparing external to internal N:P ratios revealed a strong similarity between *P. inhibens* and phytoplankton: both are characterized by a flexible internal N:P stoichiometry, i.e. internal N:P ratios positively correlate with the supplied N:P ratio in the growth medium within physiologically determined constraints.

## DISCUSSION

The Redfield ratio originally conceptualized that the internal elemental stoichiometry of marine phytoplankton (C:N:P of 106:16:1) in conjunction with its remineralization determines the oceanic contents of dissolved NO_3_^−^ and PO_4_^3−^ (Redfield 1958). As noted later, this canonical concept of constant elemental ratios did not consider nutrient cycling (including availability of iron) and phytoplankton diversity (Falkowski 2000), nor differences between oceanic provinces and aquatic ecosystems, where nutrient concentrations and N:P ratios can vary over several orders of magnitude (Quan and Falkowski 2009; Weber and Deutsch 2010). Correspondingly, algae and cyanobacteria display largely varying internal N:P ratios from <5 under conditions of P excess to >100, when N was supplied in excess (Geider and La Roche 2002; Hillebrand *et al.*^2013^). In contrast to phytoplankton, however, the quantitative contribution of heterotrophic bacterioplankton to varying elemental ratios in the ocean is not well understood. The large range of external N:P ratios (at constant C) studied here with the marine bacterium *Phaeobacter inhibens* revealed that maximal growth occurred at external N:P ratios far above Redfield (Fig. 4), that synergistic nutrient interactions suspend Liebig's law of the minimum (Fig. 5) and that *P. inhibens* adapts its internal N:P stoichiometry to the external nutrient supply, as known from phytoplankton (Fig. 6c).

The observed growth maximum at N:P ratios >16 (Fig. 4) indicates that N concentration has a strong influence on growth performance of *P. inhibens* (Table 1). The strain was detected in harbors and coastal regions, where it appears to be a competitive colonizer of abiotic and biotic marine surfaces (e.g. Seyedsayamdost *et al.*^2011^; Gram *et al.*^2015^). Coastal and estuarine habitats are often limited primarily in N, corresponding to low N:P ratios (Vitousek and Howarth 1991; Downing 1997; Elser *et al.*^2007^). The assumption that *P. inhibens* is well adapted to changing availability of reduced inorganic nitrogen is supported by the recently observed rapid consumption of supplied NH_4_^+^ during early growth, accompanied by transitory intracellular storage of N in the form of proteins, RNA and DNA (Trautwein, Rabus *et al.*, unpublished). Furthermore, high external NH_4_^+^ concentrations fueled the intracellular synthesis of community-shared secreted N-rich potential RTX toxins and of antibiotic TDA (this study; Trautwein, Rabus *et al.*, unpublished). Considering that viral-induced cell lysis of algae results in liberation of vacuole-stored NH_4_^+^ (e.g. Dortch *et al.*^1984^), bacterial colonizers of dying algae should benefit from optimization of their cellular metabolism to a transitory high N availability. Alternatively, substantial loss of NH_4_^+^ (up to ∼50% of fixed N_2_) from the colony-forming cyanobacterium *Aphanizomenon* spp. (Adam *et al.*^2016^) may create microenvironments with very high N:P ratios. The growth maximum at external N:P ratios >16 (N excess) thus underpins the importance of N (as compared to P) for niche adaptation in *P. inhibens*.

Realized growth of *P. inhibens* in parts followed a Liebig-type limitation, but also exceeded the prediction over a wide range of NH_4_^+^ and PO_4_^3–^ concentrations (Fig. 5; Fig. S4, Supporting Information). The latter indicates synergistic co-limitation of interacting nutrients, apparently resulting in superadditive growth responses at elevated NH_4_^+^ and PO_4_^3–^ concentrations. If realized growth would have been lower than predicted, this would have pinpointed to a sequential Liebig-type limitation by other essential elements or to inhibitory effects at higher NH_4_^+^ and PO_4_^3–^ concentrations (see Harpole *et al.*^2011^ for a detailed discussion). The results obtained here for *P. inhibens* thus add further evidence that multiple resource limitation occurs regularly, as observed for autotrophs globally (Elser *et al.*^2007^; Harpole *et al.*^2011^). Considering that we used a pure culture, the mechanism underlying the observed non-Liebig-type limitation suggests a biochemical dependence (Saito, Goepfert and Ritt 2008), i.e. increasing the concentration of one resource enhances the efficiency to sequester and utilize a second one for growth (including, but not necessarily N or P).

Nutrient consumption ratios tightly coupled with internal elemental composition of *P. inhibens*, and affected exponential (μ_exp_) and linear (μ_lin_) growth rates diametrically at N:P supply ratios from 4 to 67 (Fig. 6a and b; Table 1). This external N:P ratio range apparently defines the physiological limits, outside of which homeostasis in *P. inhibens* attenuates and growth rate becomes independent of nutrient supply. Both are likely the result of constrained biochemical and stoichiometric variability in cellular components and their rates of biosynthesis. Thus, *P. inhibens* obviously represents a ‘conformer’ (Meunier, Malzahn and Boersma 2014) that has its internal elemental stoichiometry determined by the external nutrient supply ratio within the organism's specific range. In case of the markedly varying internal C:P ratio in *P. inhibens* (up to ∼11-fold), maximal values of 1460 obtained under P-limitation may reflect pronounced C storage in the form of poly-(3-hydroxyalkanoates) (Trautwein, Rabus *et al.*, unpublished) at high (2 and 8 mM) external NH_4_^+^ concentrations. Highly flexible internal C:P ratios were also reported for other heterotrophic aquatic bacteria (Tezuka 1990; Godwin and Cotner 2015a,b) and for phytoplankton (Sterner *et al.*^1998^).

Overall, the marine bacterium *P. inhibens* closely resembles phytoplankton in its flexibility and physiological range to alter internal N:P stoichiometry (Fig. 6c; Klausmeier *et al.*^2004^; Franz *et al.*^2012^; Hillebrand *et al.*^2013^). Marine bacterioplankton isolates grown in batch cultures under N- or P-limitation also revealed variations in their internal N:P, C:N and C:P ratios (Vrede *et al.*^2002^). At a constant growth rate and under similar limiting conditions, the marine *Roseobacter* group member *Ruegeria pomeroyi* DSS-3 modulated its internal elemental stoichiometry (Chan *et al.*^2012^), as was also reported for aquatic heterotrophic bacteria from lakes (Godwin and Cotner 2015a,b). In contrast, the high growth rates achieved by *Escherichia coli* K-12 were suggested to determine its primarily homeostatic internal elemental composition (Makino *et al.*^2003^). Also soil microbial biomass appears to be constrained to rather constant elemental stoichiometries of C:N:P of 60:7:1, irrespective of external elemental ratios in the soil (Cleveland and Liptzin 2007). However, also here microbial internal N:P ratios ranged from 1 to >50 across different soils. Non-homeostasis in heterotrophic bacteria should rely on transitory storage of surplus nutrients, which appears advantageous especially in environments characterized by highly variable and fluctuating nutrient inputs (Persson *et al.*^2010^; Meunier, Malzahn and Boersma 2014). The here reported findings for *P. inhibens* (Figs 2–6) suggest that this bacterium is indeed well adapted to cope with such dynamic changes in N and P availability, expected to occur in its natural environment (e.g. colonized algae or higher eukaryotes, coastal areas).

Taken together, flexible internal stoichiometry is observed across organismic and trophic levels (Persson *et al.*^2010^), giving rise to complex controls of the elemental cycling and sequestration in marine and other aquatic systems alike. The flexibility of bacterial stoichiometry requires acknowledgement in the analysis of large-scale biogeochemical processes, given the central role of heterotrophic bacteria in matter and energy flows (Azam and Malfatti 2007). Bacterioplankton are not only central for carbon flux and remineralization of N and P (Cho and Azam 1988; Kirchman 1994), but also for iron (Tortell, Maldonado and Price 1996) and silicate (Bidle and Azam 1999). A flexible nutrient content in bacterioplankton potentially alters the ability of bacteria to sequester limiting nutrients and to compete with phytoplankton under inorganic nutrient limitation. At the same time, stoichiometric flexibility within strains, as shown here, is only one mechanism increasing the flexibility of bacterial effects on organic matter cycling and element fluxes. In complex natural communities, compositional shifts of strains within taxa and/or of different taxa should increase the overall stoichiometric flexibility of the bacterial component. This becomes even more striking given the fact that organic matter cycling and the relative importance of bacterioplankton respond interactively to nutrient supply and temperature (Wohlers-Zöllner *et al.*^2011^).

## Supplementary Material

Supplemental material Supplementary data are available at *FEMSEC* online.
