# Supplementary material for: Non-Redfield, nutrient synergy and flexible internal elemental stoichiometry in a marine bacterium
Source: FEMS Microbiol Ecol. 2017 May 9;93(5):fix059. doi: 10.1093/femsec/fix059 (PMC5458051; doi:10.1093/femsec/fix059)
Supplement: Supplemental material — Supplementary data are available at FEMSEC online. [file fix059_supp.pdf]

Supporting information

Non-Redfield, nutrient synergy, and flexible internal elemental stoichiometry in a marine bacterium

Trautwein *et al.*

CONTENTS

Figure S1 Cultivation workflow in physiological experiments……………………………………2

Figure S2 Compiled growth curves with fitted LDR function for 415 different NH_4^+ and PO_4^{3-} concentration pairs……………..…………………………………………..……..…3

Figure S3 High-resolution growth curves with fitted LDR function for five selected NH_4^+ and PO_4^{3-} concentration pairs.……………………………………………………….………31

Figure S4 Comparison of experimental data with model prediction based on Liebig's law of the minimum…..…………………………………………………………………….32

Figure S5 Experimental data with fitted Monod function………………………………………..33

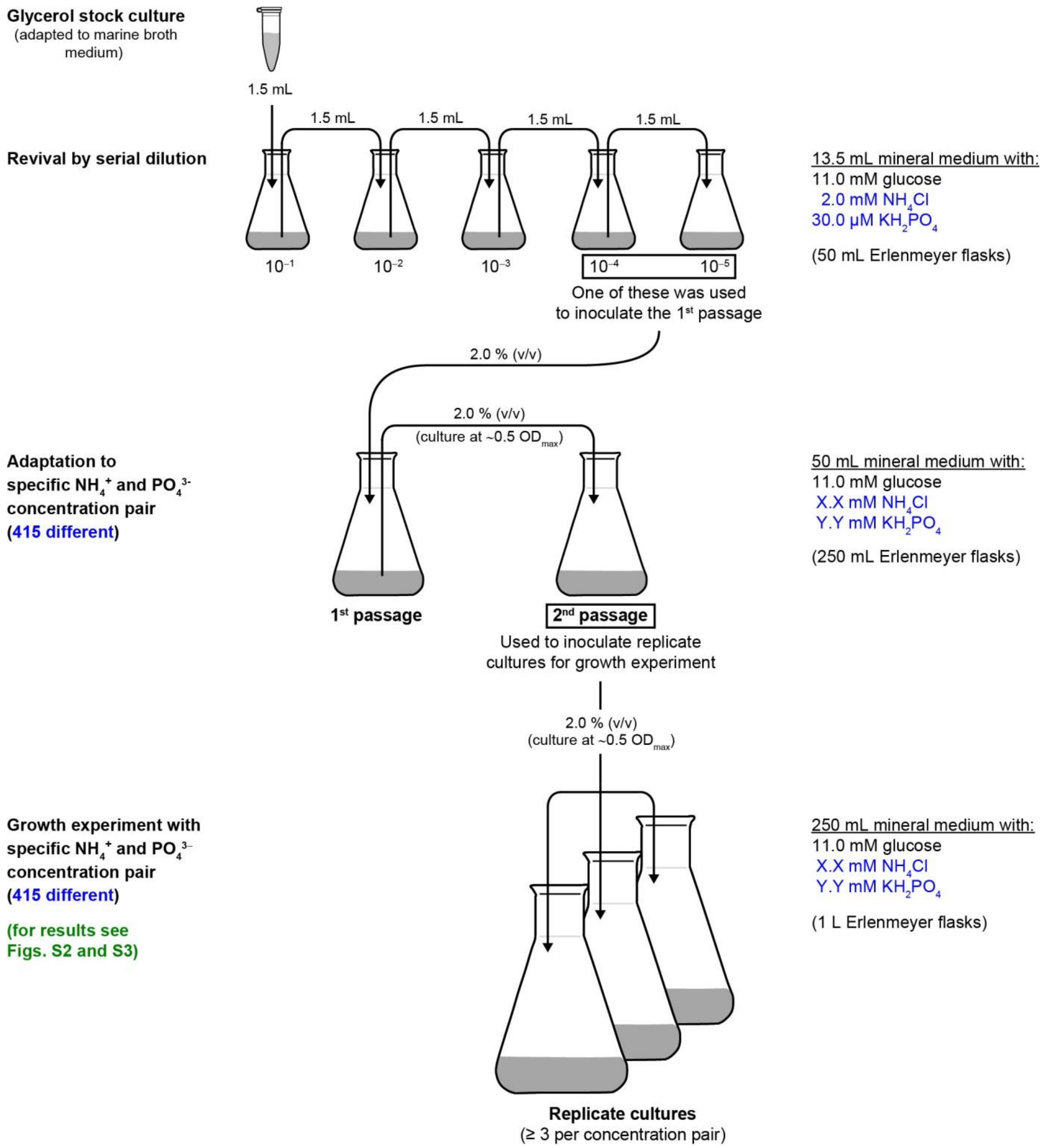

Figure S1 Cultivation workflow in physiological experiments. Each growth experiment covering one of the 415 different NH_4^+ and PO_4^{3-} concentration pairs followed a stringent cultivation procedure, starting from an individual glycerol stock culture. Glassware was specifically cleansed with HCl at PO_4^{3-} concentrations <50 µM.

.

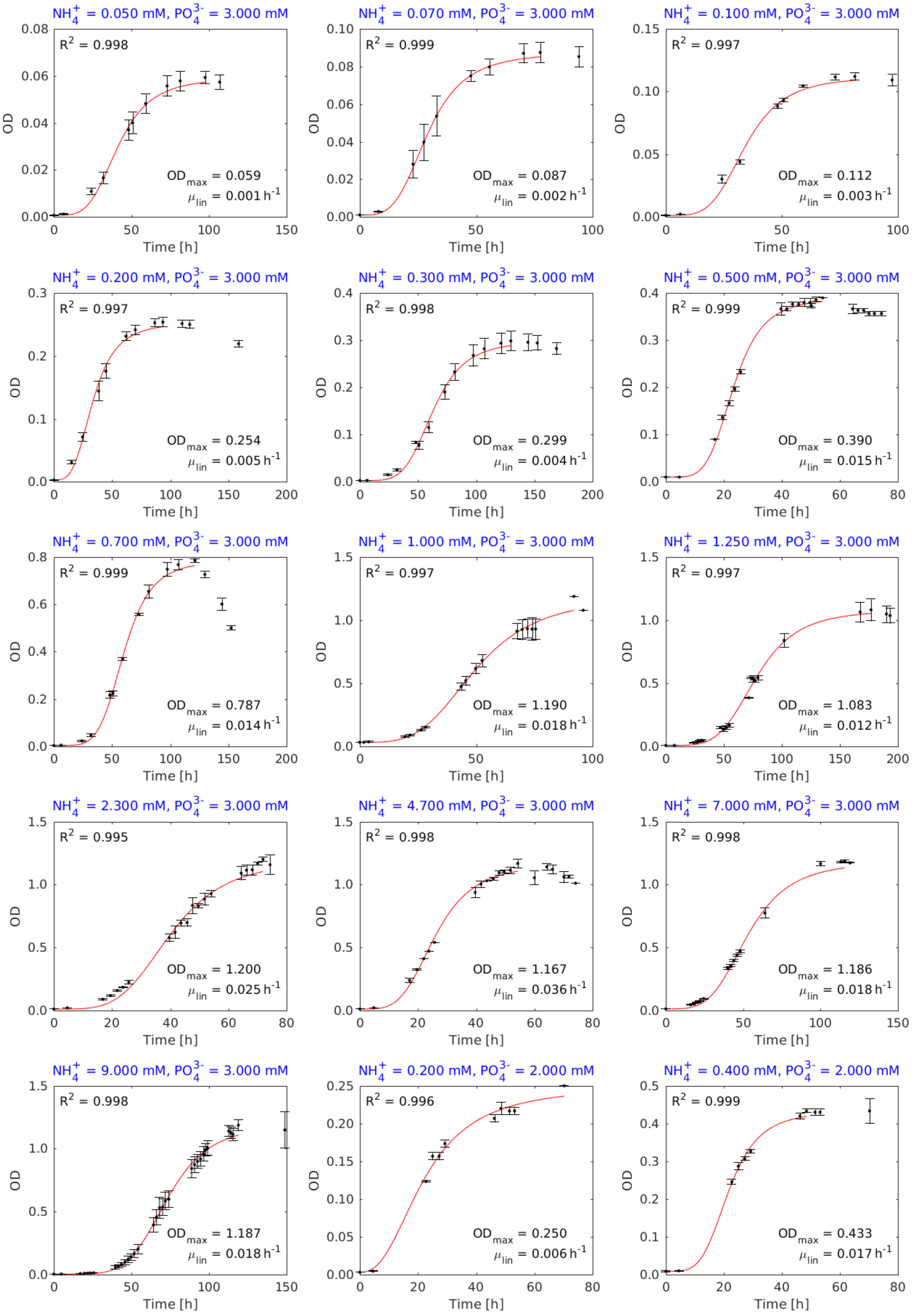

NH4+ = 0.050 mM, PO43- = 3.000 mM
R2 = 0.998
ODmax = 0.059
μlin = 0.001 h-1
NH4+ = 0.070 mM, PO43- = 3.000 mM
R2 = 0.999
ODmax = 0.087
μlin = 0.002 h-1
NH4+ = 0.100 mM, PO43- = 3.000 mM
R2 = 0.997
ODmax = 0.112
μlin = 0.003 h-1
NH4+ = 0.200 mM, PO43- = 3.000 mM
R2 = 0.997
ODmax = 0.254
μlin = 0.005 h-1
NH4+ = 0.300 mM, PO43- = 3.000 mM
R2 = 0.998
ODmax = 0.299
μlin = 0.004 h-1
NH4+ = 0.500 mM, PO43- = 3.000 mM
R2 = 0.999
ODmax = 0.390
μlin = 0.015 h-1
NH4+ = 0.700 mM, PO43- = 3.000 mM
R2 = 0.999
ODmax = 0.787
μlin = 0.014 h-1
NH4+ = 1.000 mM, PO43- = 3.000 mM
R2 = 0.997
ODmax = 1.190
μlin = 0.018 h-1
NH4+ = 1.250 mM, PO43- = 3.000 mM
R2 = 0.997
ODmax = 1.083
μlin = 0.012 h-1
NH4+ = 2.300 mM, PO43- = 3.000 mM
R2 = 0.995
ODmax = 1.200
μlin = 0.025 h-1
NH4+ = 4.700 mM, PO43- = 3.000 mM
R2 = 0.998
ODmax = 1.167
μlin = 0.036 h-1
NH4+ = 7.000 mM, PO43- = 3.000 mM
R2 = 0.998
ODmax = 1.186
μlin = 0.018 h-1
NH4+ = 9.000 mM, PO43- = 3.000 mM
R2 = 0.998
ODmax = 1.187
μlin = 0.018 h-1
NH4+ = 0.200 mM, PO43- = 2.000 mM
R2 = 0.996
ODmax = 0.250
μlin = 0.006 h-1
NH4+ = 0.400 mM, PO43- = 2.000 mM
R2 = 0.999
ODmax = 0.433
μlin = 0.017 h-1
OD
Time [h]

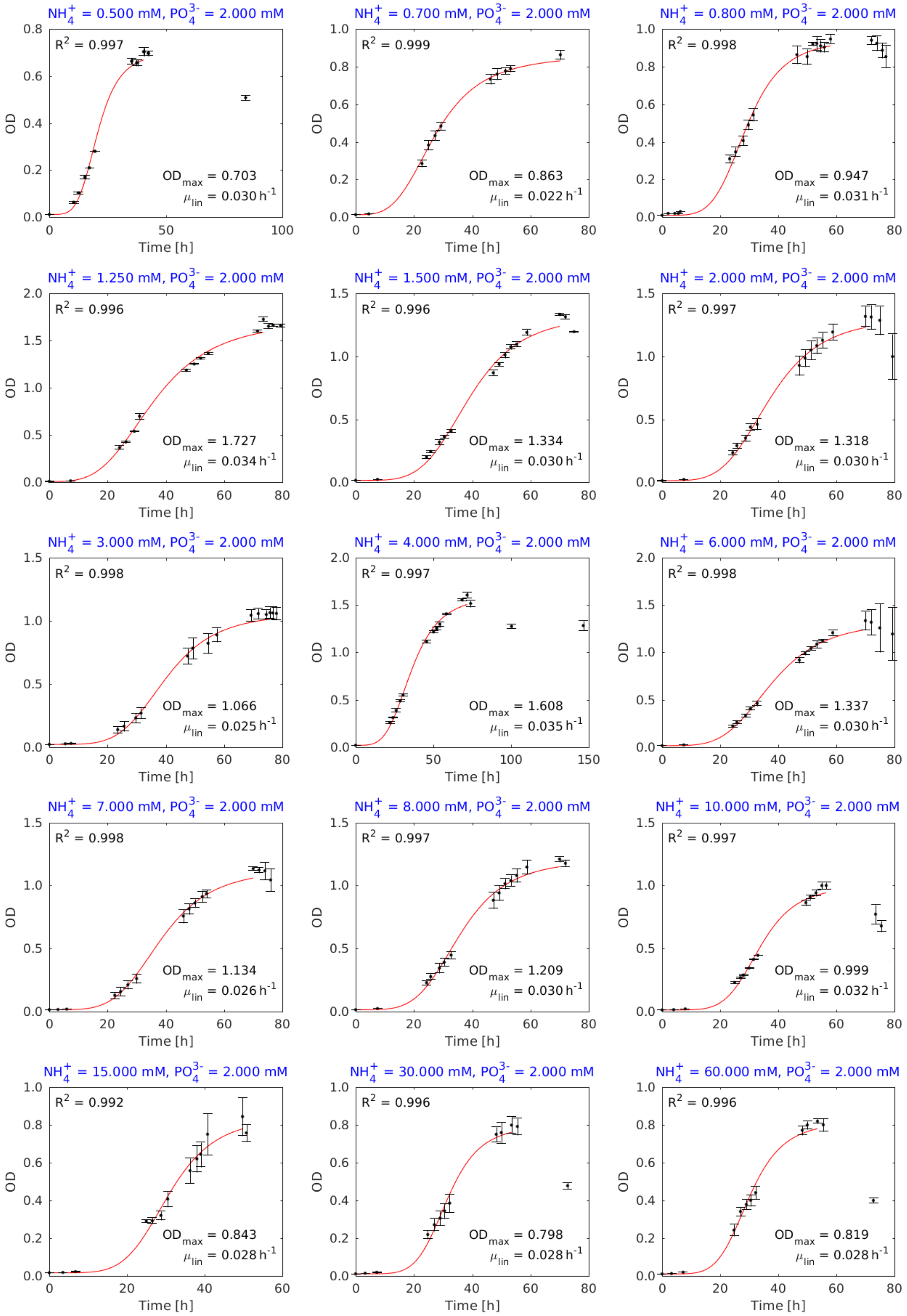

NH4+ = 0.500 mM, PO43- = 2.000 mM
R2 = 0.997
ODmax = 0.703
μlin = 0.030 h-1
NH4+ = 0.700 mM, PO43- = 2.000 mM
R2 = 0.999
ODmax = 0.863
μlin = 0.022 h-1
NH4+ = 0.800 mM, PO43- = 2.000 mM
R2 = 0.998
ODmax = 0.947
μlin = 0.031 h-1
NH4+ = 1.250 mM, PO43- = 2.000 mM
R2 = 0.996
ODmax = 1.727
μlin = 0.034 h-1
NH4+ = 1.500 mM, PO43- = 2.000 mM
R2 = 0.996
ODmax = 1.334
μlin = 0.030 h-1
NH4+ = 2.000 mM, PO43- = 2.000 mM
R2 = 0.997
ODmax = 1.318
μlin = 0.030 h-1
NH4+ = 3.000 mM, PO43- = 2.000 mM
R2 = 0.998
ODmax = 1.066
μlin = 0.025 h-1
NH4+ = 4.000 mM, PO43- = 2.000 mM
R2 = 0.997
ODmax = 1.608
μlin = 0.035 h-1
NH4+ = 6.000 mM, PO43- = 2.000 mM
R2 = 0.998
ODmax = 1.337
μlin = 0.030 h-1
NH4+ = 7.000 mM, PO43- = 2.000 mM
R2 = 0.998
ODmax = 1.134
μlin = 0.026 h-1
NH4+ = 8.000 mM, PO43- = 2.000 mM
R2 = 0.997
ODmax = 1.209
μlin = 0.030 h-1
NH4+ = 10.000 mM, PO43- = 2.000 mM
R2 = 0.997
ODmax = 0.999
μlin = 0.032 h-1
NH4+ = 15.000 mM, PO43- = 2.000 mM
R2 = 0.992
ODmax = 0.843
μlin = 0.028 h-1
NH4+ = 30.000 mM, PO43- = 2.000 mM
R2 = 0.996
ODmax = 0.798
μlin = 0.028 h-1
NH4+ = 60.000 mM, PO43- = 2.000 mM
R2 = 0.996
ODmax = 0.819
μlin = 0.028 h-1
OD
Time [h]

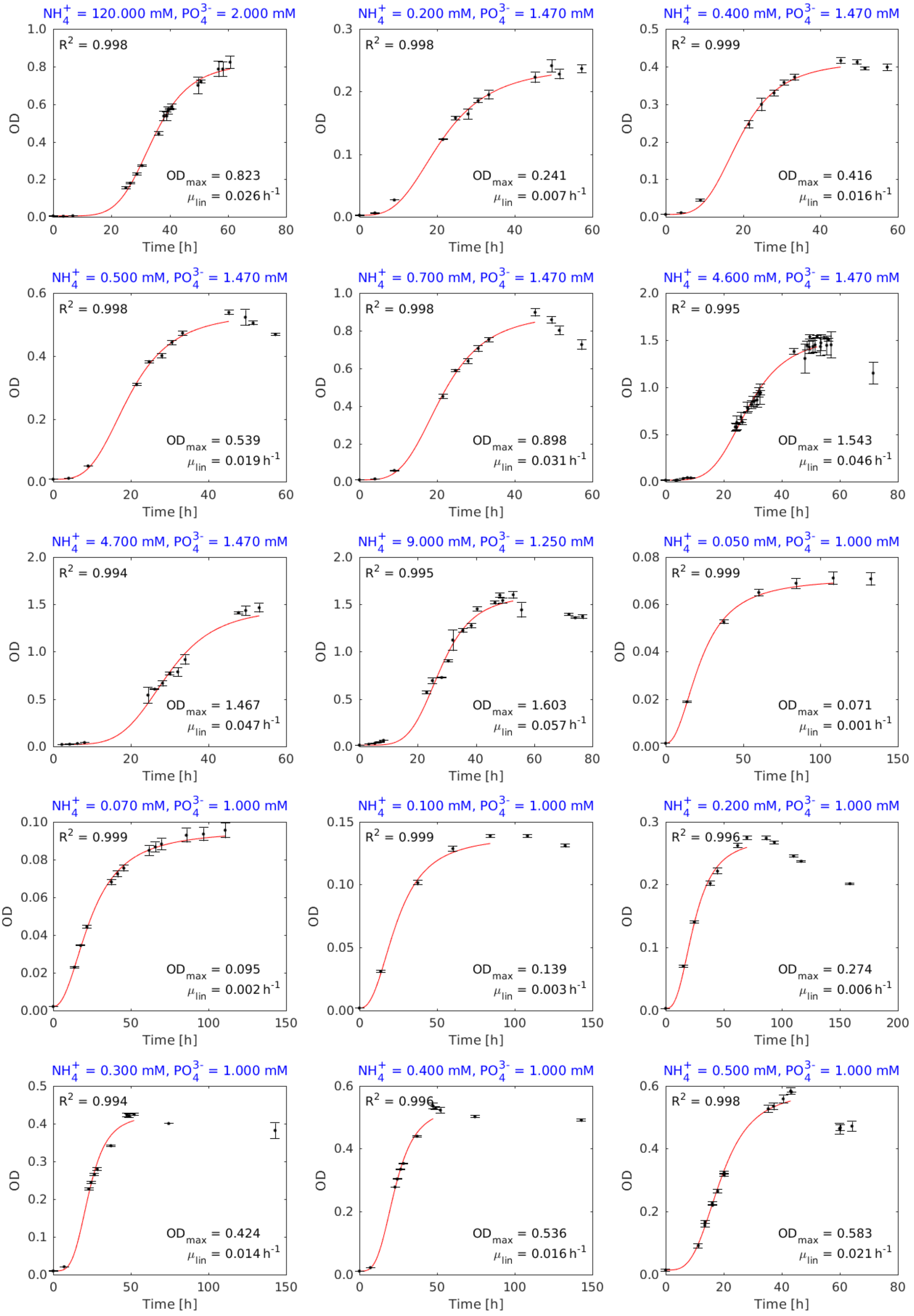

NH_4^+ = 120.000 mM, PO_4^{3-} = 2.000 mM
R^2 = 0.998
OD_{max} = 0.823
μ_{lin} = 0.026 h^{-1}
NH_4^+ = 0.200 mM, PO_4^{3-} = 1.470 mM
R^2 = 0.998
OD_{max} = 0.241
μ_{lin} = 0.007 h^{-1}
NH_4^+ = 0.400 mM, PO_4^{3-} = 1.470 mM
R^2 = 0.999
OD_{max} = 0.416
μ_{lin} = 0.016 h^{-1}
NH_4^+ = 0.500 mM, PO_4^{3-} = 1.470 mM
R^2 = 0.998
OD_{max} = 0.539
μ_{lin} = 0.019 h^{-1}
NH_4^+ = 0.700 mM, PO_4^{3-} = 1.470 mM
R^2 = 0.998
OD_{max} = 0.898
μ_{lin} = 0.031 h^{-1}
NH_4^+ = 4.600 mM, PO_4^{3-} = 1.470 mM
R^2 = 0.995
OD_{max} = 1.543
μ_{lin} = 0.046 h^{-1}
NH_4^+ = 4.700 mM, PO_4^{3-} = 1.470 mM
R^2 = 0.994
OD_{max} = 1.467
μ_{lin} = 0.047 h^{-1}
NH_4^+ = 9.000 mM, PO_4^{3-} = 1.250 mM
R^2 = 0.995
OD_{max} = 1.603
μ_{lin} = 0.057 h^{-1}
NH_4^+ = 0.050 mM, PO_4^{3-} = 1.000 mM
R^2 = 0.999
OD_{max} = 0.071
μ_{lin} = 0.001 h^{-1}
NH_4^+ = 0.070 mM, PO_4^{3-} = 1.000 mM
R^2 = 0.999
OD_{max} = 0.095
μ_{lin} = 0.002 h^{-1}
NH_4^+ = 0.100 mM, PO_4^{3-} = 1.000 mM
R^2 = 0.999
OD_{max} = 0.139
μ_{lin} = 0.003 h^{-1}
NH_4^+ = 0.200 mM, PO_4^{3-} = 1.000 mM
R^2 = 0.996
OD_{max} = 0.274
μ_{lin} = 0.006 h^{-1}
NH_4^+ = 0.300 mM, PO_4^{3-} = 1.000 mM
R^2 = 0.994
OD_{max} = 0.424
μ_{lin} = 0.014 h^{-1}
NH_4^+ = 0.400 mM, PO_4^{3-} = 1.000 mM
R^2 = 0.996
OD_{max} = 0.536
μ_{lin} = 0.016 h^{-1}
NH_4^+ = 0.500 mM, PO_4^{3-} = 1.000 mM
R^2 = 0.998
OD_{max} = 0.583
μ_{lin} = 0.021 h^{-1}
OD
Time [h]

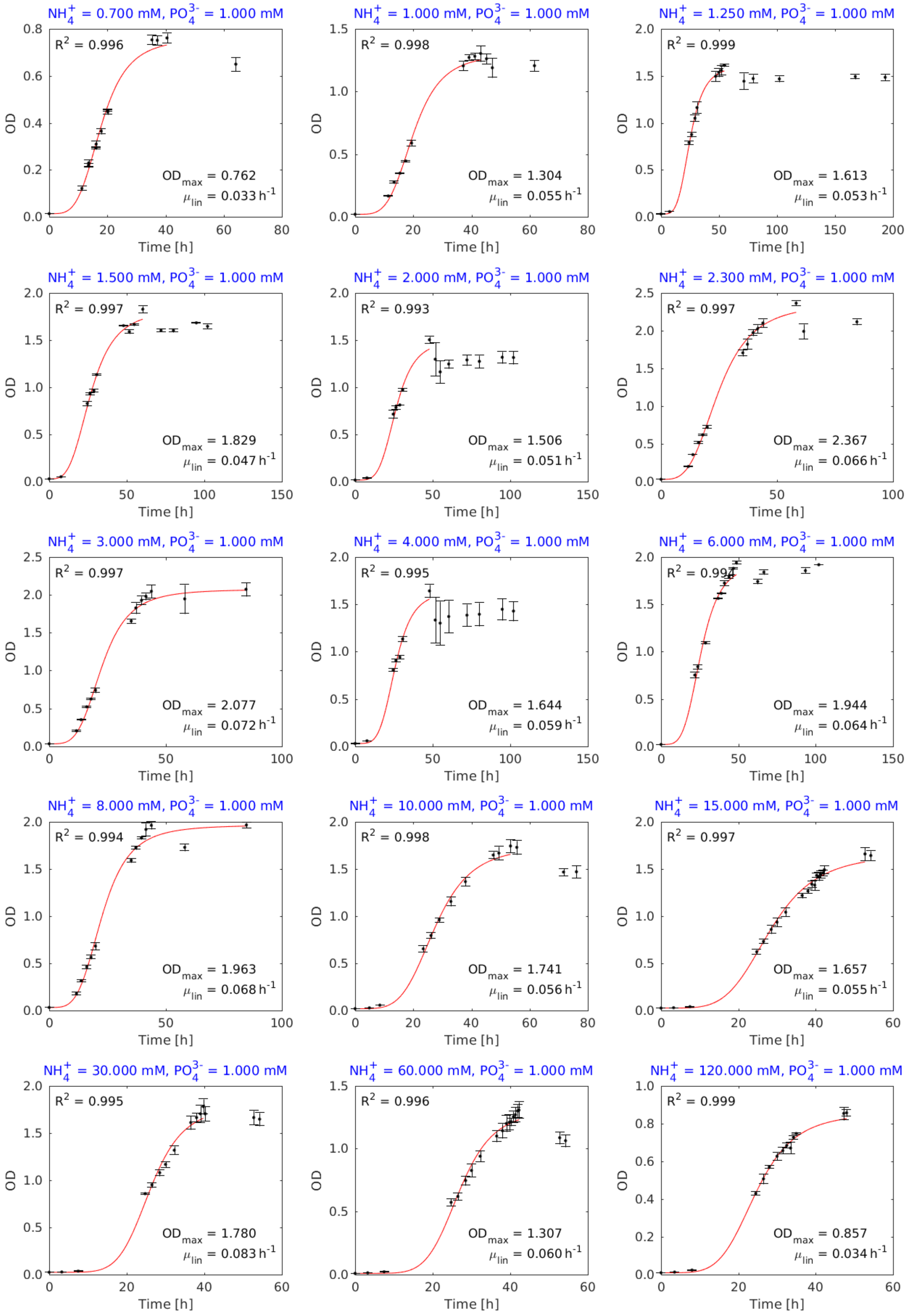

NH_4^+ = 0.700 mM, PO_4^{3-} = 1.000 mM
R^2 = 0.996
OD_{max} = 0.762
μ_{lin} = 0.033 h^{-1}
NH_4^+ = 1.000 mM, PO_4^{3-} = 1.000 mM
R^2 = 0.998
OD_{max} = 1.304
μ_{lin} = 0.055 h^{-1}
NH_4^+ = 1.250 mM, PO_4^{3-} = 1.000 mM
R^2 = 0.999
OD_{max} = 1.613
μ_{lin} = 0.053 h^{-1}
NH_4^+ = 1.500 mM, PO_4^{3-} = 1.000 mM
R^2 = 0.997
OD_{max} = 1.829
μ_{lin} = 0.047 h^{-1}
NH_4^+ = 2.000 mM, PO_4^{3-} = 1.000 mM
R^2 = 0.993
OD_{max} = 1.506
μ_{lin} = 0.051 h^{-1}
NH_4^+ = 2.300 mM, PO_4^{3-} = 1.000 mM
R^2 = 0.997
OD_{max} = 2.367
μ_{lin} = 0.066 h^{-1}
NH_4^+ = 3.000 mM, PO_4^{3-} = 1.000 mM
R^2 = 0.997
OD_{max} = 2.077
μ_{lin} = 0.072 h^{-1}
NH_4^+ = 4.000 mM, PO_4^{3-} = 1.000 mM
R^2 = 0.995
OD_{max} = 1.644
μ_{lin} = 0.059 h^{-1}
NH_4^+ = 6.000 mM, PO_4^{3-} = 1.000 mM
R^2 = 0.99
OD_{max} = 1.944
μ_{lin} = 0.064 h^{-1}
NH_4^+ = 8.000 mM, PO_4^{3-} = 1.000 mM
R^2 = 0.994
OD_{max} = 1.963
μ_{lin} = 0.068 h^{-1}
NH_4^+ = 10.000 mM, PO_4^{3-} = 1.000 mM
R^2 = 0.998
OD_{max} = 1.741
μ_{lin} = 0.056 h^{-1}
NH_4^+ = 15.000 mM, PO_4^{3-} = 1.000 mM
R^2 = 0.997
OD_{max} = 1.657
μ_{lin} = 0.055 h^{-1}
NH_4^+ = 30.000 mM, PO_4^{3-} = 1.000 mM
R^2 = 0.995
OD_{max} = 1.780
μ_{lin} = 0.083 h^{-1}
NH_4^+ = 60.000 mM, PO_4^{3-} = 1.000 mM
R^2 = 0.996
OD_{max} = 1.307
μ_{lin} = 0.060 h^{-1}
NH_4^+ = 120.000 mM, PO_4^{3-} = 1.000 mM
R^2 = 0.999
OD_{max} = 0.857
μ_{lin} = 0.034 h^{-1}
OD
Time [h]

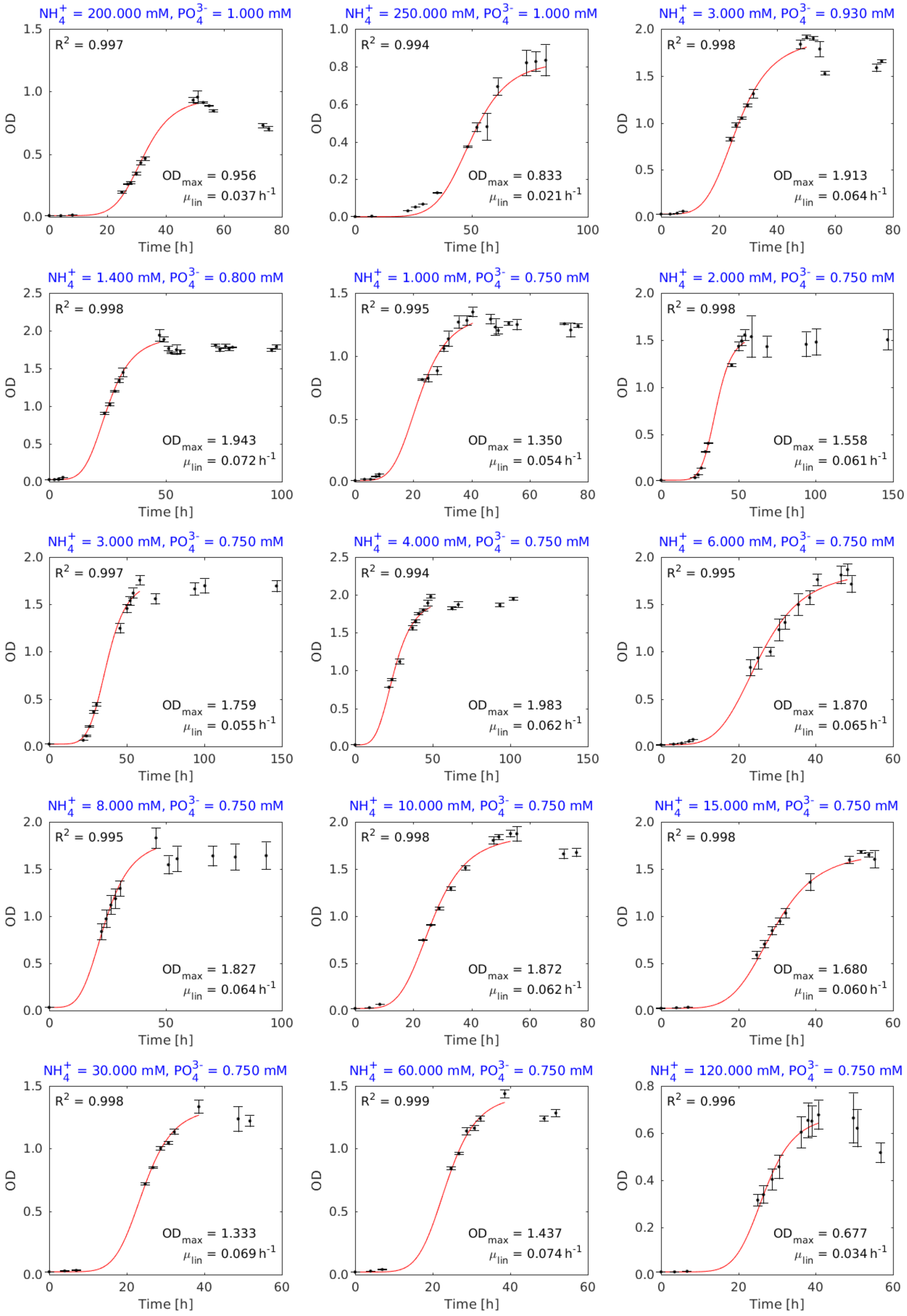
NH4+ = 200.000 mM, PO43- = 1.000 mM
R2 = 0.997
ODmax = 0.956
μlin = 0.037 h-1
NH4+ = 250.000 mM, PO43- = 1.000 mM
R2 = 0.994
ODmax = 0.833
μlin = 0.021 h-1
NH4+ = 3.000 mM, PO43- = 0.930 mM
R2 = 0.998
ODmax = 1.913
μlin = 0.064 h-1
NH4+ = 1.400 mM, PO43- = 0.800 mM
R2 = 0.998
ODmax = 1.943
μlin = 0.072 h-1
NH4+ = 1.000 mM, PO43- = 0.750 mM
R2 = 0.995
ODmax = 1.350
μlin = 0.054 h-1
NH4+ = 2.000 mM, PO43- = 0.750 mM
R2 = 0.998
ODmax = 1.558
μlin = 0.061 h-1
NH4+ = 3.000 mM, PO43- = 0.750 mM
R2 = 0.997
ODmax = 1.759
μlin = 0.055 h-1
NH4+ = 4.000 mM, PO43- = 0.750 mM
R2 = 0.994
ODmax = 1.983
μlin = 0.062 h-1
NH4+ = 6.000 mM, PO43- = 0.750 mM
R2 = 0.995
ODmax = 1.870
μlin = 0.065 h-1
NH4+ = 8.000 mM, PO43- = 0.750 mM
R2 = 0.995
ODmax = 1.827
μlin = 0.064 h-1
NH4+ = 10.000 mM, PO43- = 0.750 mM
R2 = 0.998
ODmax = 1.872
μlin = 0.062 h-1
NH4+ = 15.000 mM, PO43- = 0.750 mM
R2 = 0.998
ODmax = 1.680
μlin = 0.060 h-1
NH4+ = 30.000 mM, PO43- = 0.750 mM
R2 = 0.998
ODmax = 1.333
μlin = 0.069 h-1
NH4+ = 60.000 mM, PO43- = 0.750 mM
R2 = 0.999
ODmax = 1.437
μlin = 0.074 h-1
NH4+ = 120.000 mM, PO43- = 0.750 mM
R2 = 0.996
ODmax = 0.677
μlin = 0.034 h-1
OD
Time [h]

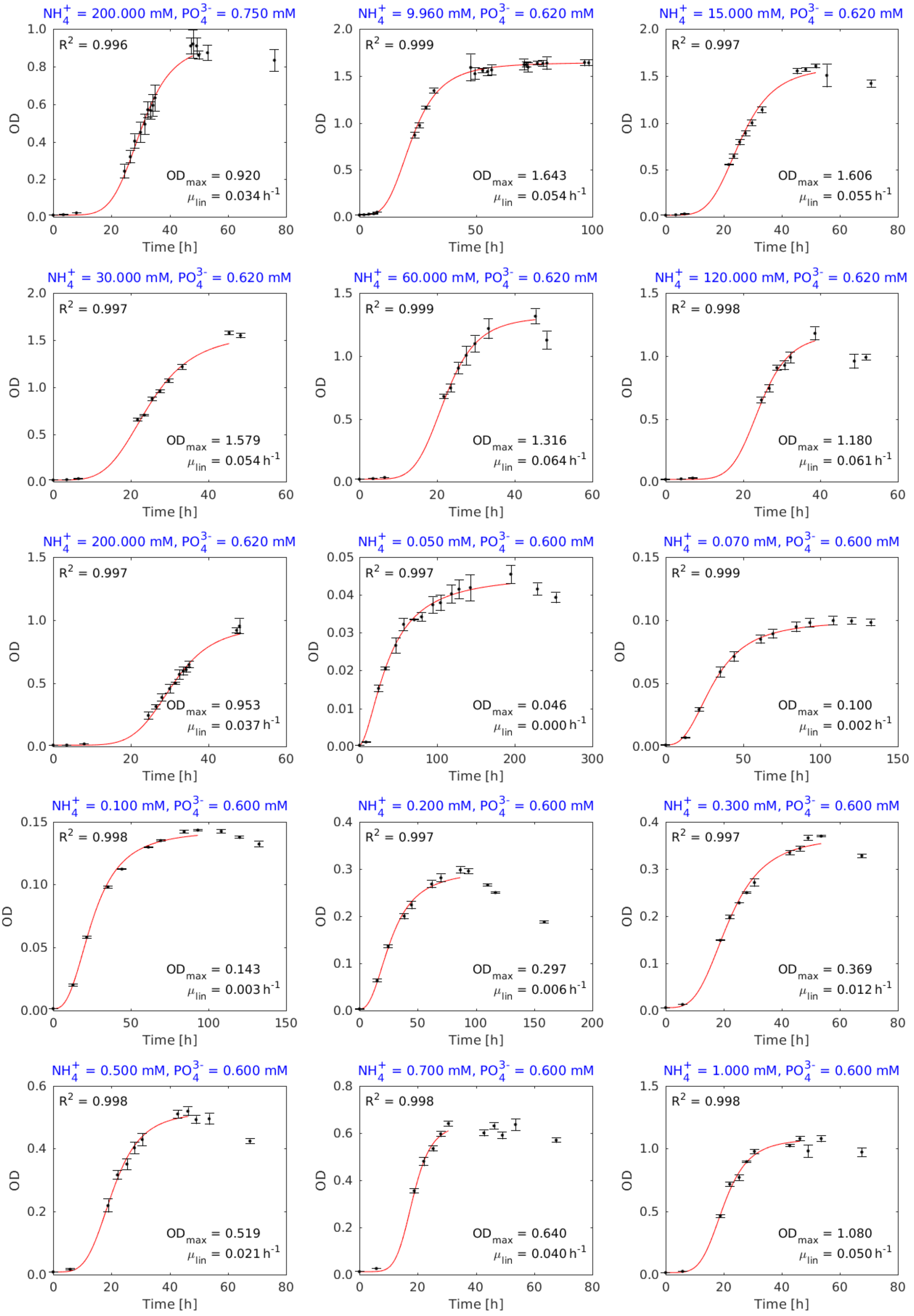
NH$_4^+$ = 200.000 mM, PO$_4^{3-}$ = 0.750 mM
R^2 = 0.996
OD$_{max}$ = 0.920
μ_{lin} = 0.034 h^{-1}
NH$_4^+$ = 9.960 mM, PO$_4^{3-}$ = 0.620 mM
R^2 = 0.999
OD$_{max}$ = 1.643
μ_{lin} = 0.054 h^{-1}
NH$_4^+$ = 15.000 mM, PO$_4^{3-}$ = 0.620 mM
R^2 = 0.997
OD$_{max}$ = 1.606
μ_{lin} = 0.055 h^{-1}
NH$_4^+$ = 30.000 mM, PO$_4^{3-}$ = 0.620 mM
R^2 = 0.997
OD$_{max}$ = 1.579
μ_{lin} = 0.054 h^{-1}
NH$_4^+$ = 60.000 mM, PO$_4^{3-}$ = 0.620 mM
R^2 = 0.999
OD$_{max}$ = 1.316
μ_{lin} = 0.064 h^{-1}
NH$_4^+$ = 120.000 mM, PO$_4^{3-}$ = 0.620 mM
R^2 = 0.998
OD$_{max}$ = 1.180
μ_{lin} = 0.061 h^{-1}
NH$_4^+$ = 200.000 mM, PO$_4^{3-}$ = 0.620 mM
R^2 = 0.997
OD$_{max}$ = 0.953
μ_{lin} = 0.037 h^{-1}
NH$_4^+$ = 0.050 mM, PO$_4^{3-}$ = 0.600 mM
R^2 = 0.997
OD$_{max}$ = 0.046
μ_{lin} = 0.000 h^{-1}
NH$_4^+$ = 0.070 mM, PO$_4^{3-}$ = 0.600 mM
R^2 = 0.999
OD$_{max}$ = 0.100
μ_{lin} = 0.002 h^{-1}
NH$_4^+$ = 0.100 mM, PO$_4^{3-}$ = 0.600 mM
R^2 = 0.998
OD$_{max}$ = 0.143
μ_{lin} = 0.003 h^{-1}
NH$_4^+$ = 0.200 mM, PO$_4^{3-}$ = 0.600 mM
R^2 = 0.997
OD$_{max}$ = 0.297
μ_{lin} = 0.006 h^{-1}
NH$_4^+$ = 0.300 mM, PO$_4^{3-}$ = 0.600 mM
R^2 = 0.997
OD$_{max}$ = 0.369
μ_{lin} = 0.012 h^{-1}
NH$_4^+$ = 0.500 mM, PO$_4^{3-}$ = 0.600 mM
R^2 = 0.998
OD$_{max}$ = 0.519
μ_{lin} = 0.021 h^{-1}
NH$_4^+$ = 0.700 mM, PO$_4^{3-}$ = 0.600 mM
R^2 = 0.998
OD$_{max}$ = 0.640
μ_{lin} = 0.040 h^{-1}
NH$_4^+$ = 1.000 mM, PO$_4^{3-}$ = 0.600 mM
R^2 = 0.998
OD$_{max}$ = 1.080
μ_{lin} = 0.050 h^{-1}
OD
Time [h]

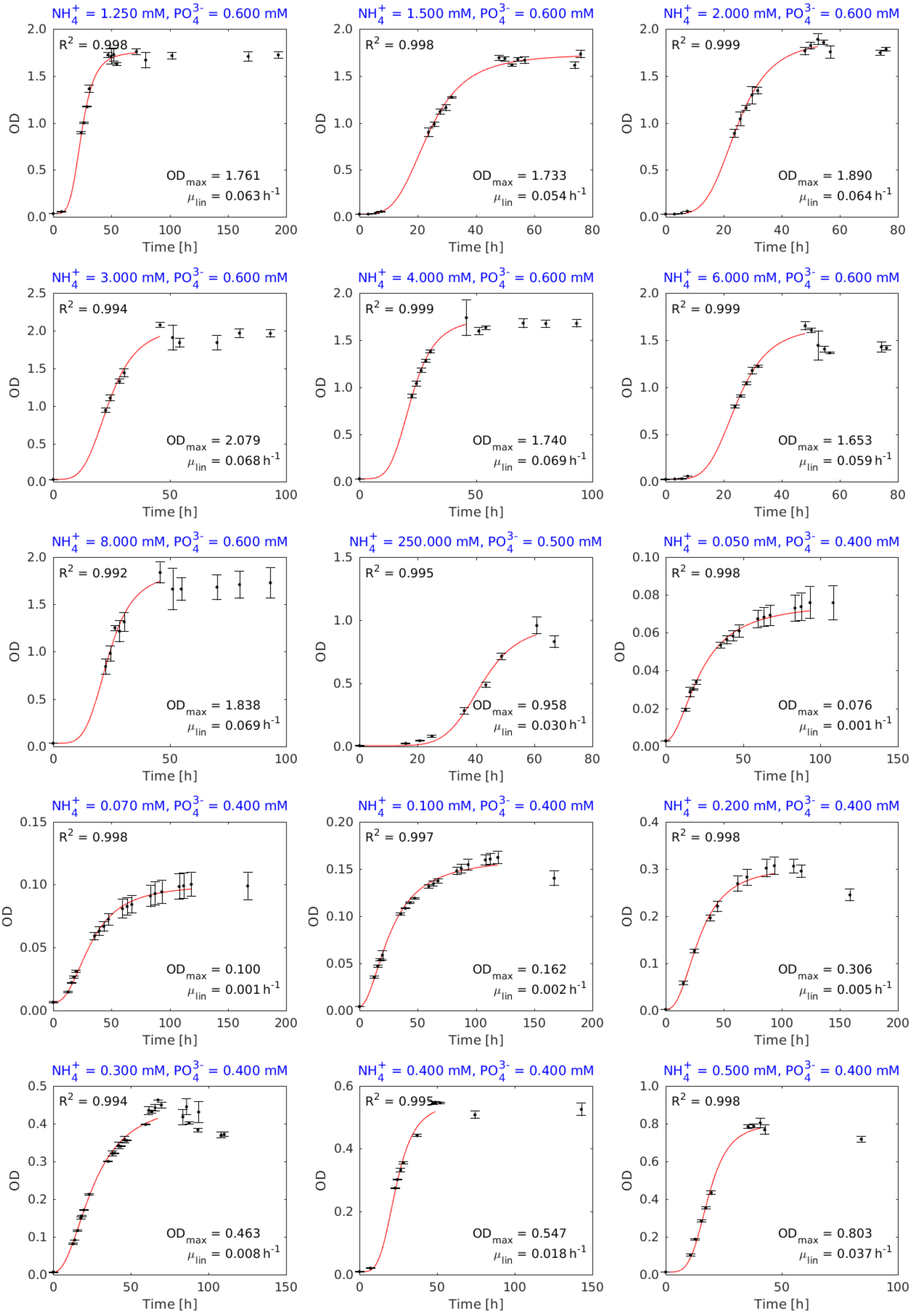

NH$_4^+$ = 1.250 mM, PO$_4^{3-}$ = 0.600 mM
R^2 = 0.998
OD_{max} = 1.761
μ_{lin} = 0.063 h^{-1}
NH$_4^+$ = 1.500 mM, PO$_4^{3-}$ = 0.600 mM
R^2 = 0.998
OD_{max} = 1.733
μ_{lin} = 0.054 h^{-1}
NH$_4^+$ = 2.000 mM, PO$_4^{3-}$ = 0.600 mM
R^2 = 0.999
OD_{max} = 1.890
μ_{lin} = 0.064 h^{-1}
NH$_4^+$ = 3.000 mM, PO$_4^{3-}$ = 0.600 mM
R^2 = 0.994
OD_{max} = 2.079
μ_{lin} = 0.068 h^{-1}
NH$_4^+$ = 4.000 mM, PO$_4^{3-}$ = 0.600 mM
R^2 = 0.999
OD_{max} = 1.740
μ_{lin} = 0.069 h^{-1}
NH$_4^+$ = 6.000 mM, PO$_4^{3-}$ = 0.600 mM
R^2 = 0.999
OD_{max} = 1.653
μ_{lin} = 0.059 h^{-1}
NH$_4^+$ = 8.000 mM, PO$_4^{3-}$ = 0.600 mM
R^2 = 0.992
OD_{max} = 1.838
μ_{lin} = 0.069 h^{-1}
NH$_4^+$ = 250.000 mM, PO$_4^{3-}$ = 0.500 mM
R^2 = 0.995
OD_{max} = 0.958
μ_{lin} = 0.030 h^{-1}
NH$_4^+$ = 0.050 mM, PO$_4^{3-}$ = 0.400 mM
R^2 = 0.998
OD_{max} = 0.076
μ_{lin} = 0.001 h^{-1}
NH$_4^+$ = 0.070 mM, PO$_4^{3-}$ = 0.400 mM
R^2 = 0.998
OD_{max} = 0.100
μ_{lin} = 0.001 h^{-1}
NH$_4^+$ = 0.100 mM, PO$_4^{3-}$ = 0.400 mM
R^2 = 0.997
OD_{max} = 0.162
μ_{lin} = 0.002 h^{-1}
NH$_4^+$ = 0.200 mM, PO$_4^{3-}$ = 0.400 mM
R^2 = 0.998
OD_{max} = 0.306
μ_{lin} = 0.005 h^{-1}
NH$_4^+$ = 0.300 mM, PO$_4^{3-}$ = 0.400 mM
R^2 = 0.994
OD_{max} = 0.463
μ_{lin} = 0.008 h^{-1}
NH$_4^+$ = 0.400 mM, PO$_4^{3-}$ = 0.400 mM
R^2 = 0.995
OD_{max} = 0.547
μ_{lin} = 0.018 h^{-1}
NH$_4^+$ = 0.500 mM, PO$_4^{3-}$ = 0.400 mM
R^2 = 0.998
OD_{max} = 0.803
μ_{lin} = 0.037 h^{-1}
OD
Time [h]

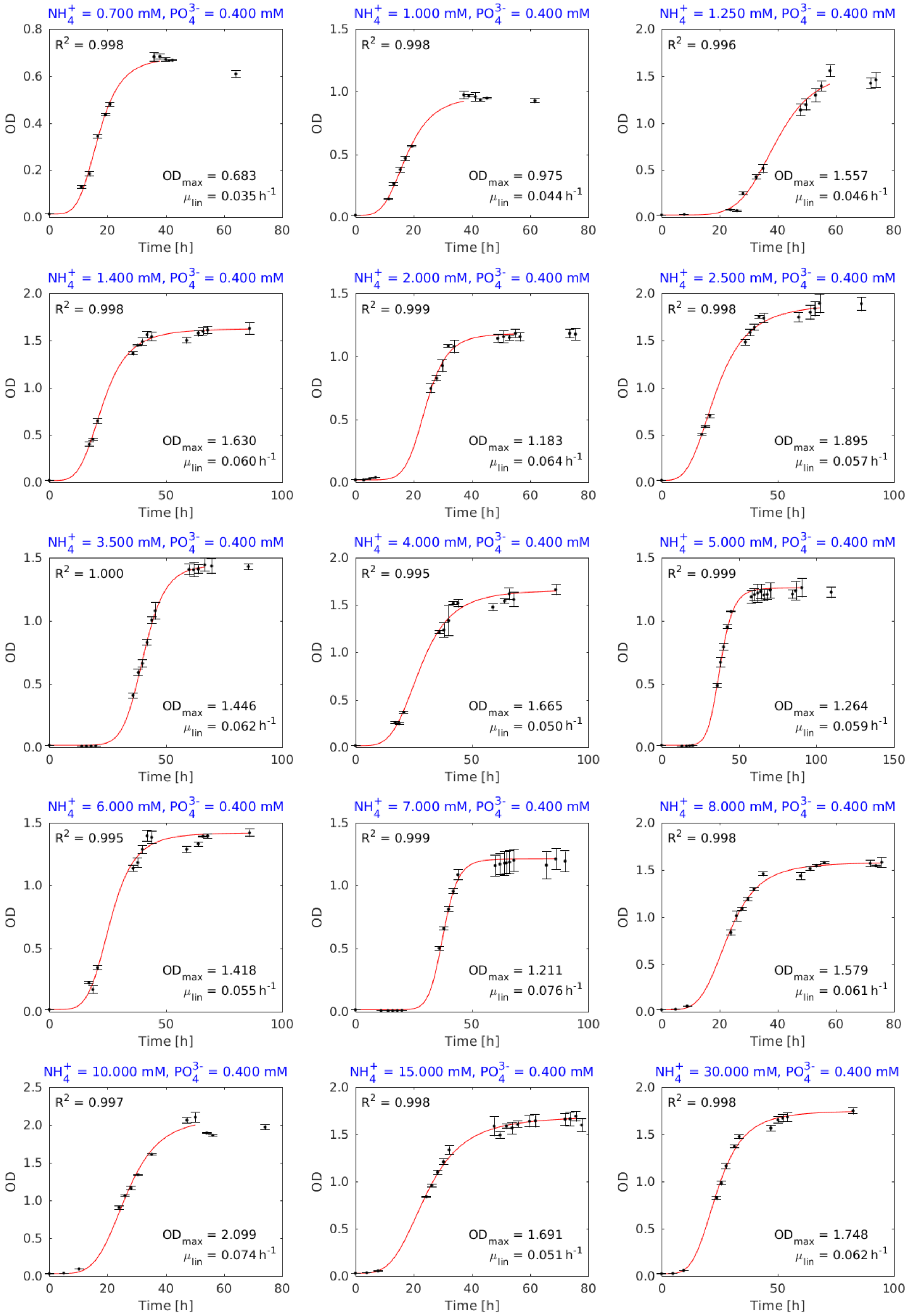
NH4+ = 0.700 mM, PO43- = 0.400 mM
R2 = 0.998
ODmax = 0.683
μlin = 0.035 h-1
NH4+ = 1.000 mM, PO43- = 0.400 mM
R2 = 0.998
ODmax = 0.975
μlin = 0.044 h-1
NH4+ = 1.250 mM, PO43- = 0.400 mM
R2 = 0.996
ODmax = 1.557
μlin = 0.046 h-1
NH4+ = 1.400 mM, PO43- = 0.400 mM
R2 = 0.998
ODmax = 1.630
μlin = 0.060 h-1
NH4+ = 2.000 mM, PO43- = 0.400 mM
R2 = 0.999
ODmax = 1.183
μlin = 0.064 h-1
NH4+ = 2.500 mM, PO43- = 0.400 mM
R2 = 0.998
ODmax = 1.895
μlin = 0.057 h-1
NH4+ = 3.500 mM, PO43- = 0.400 mM
R2 = 1.000
ODmax = 1.446
μlin = 0.062 h-1
NH4+ = 4.000 mM, PO43- = 0.400 mM
R2 = 0.995
ODmax = 1.665
μlin = 0.050 h-1
NH4+ = 5.000 mM, PO43- = 0.400 mM
R2 = 0.999
ODmax = 1.264
μlin = 0.059 h-1
NH4+ = 6.000 mM, PO43- = 0.400 mM
R2 = 0.995
ODmax = 1.418
μlin = 0.055 h-1
NH4+ = 7.000 mM, PO43- = 0.400 mM
R2 = 0.999
ODmax = 1.211
μlin = 0.076 h-1
NH4+ = 8.000 mM, PO43- = 0.400 mM
R2 = 0.998
ODmax = 1.579
μlin = 0.061 h-1
NH4+ = 10.000 mM, PO43- = 0.400 mM
R2 = 0.997
ODmax = 2.099
μlin = 0.074 h-1
NH4+ = 15.000 mM, PO43- = 0.400 mM
R2 = 0.998
ODmax = 1.691
μlin = 0.051 h-1
NH4+ = 30.000 mM, PO43- = 0.400 mM
R2 = 0.998
ODmax = 1.748
μlin = 0.062 h-1
OD
Time [h]

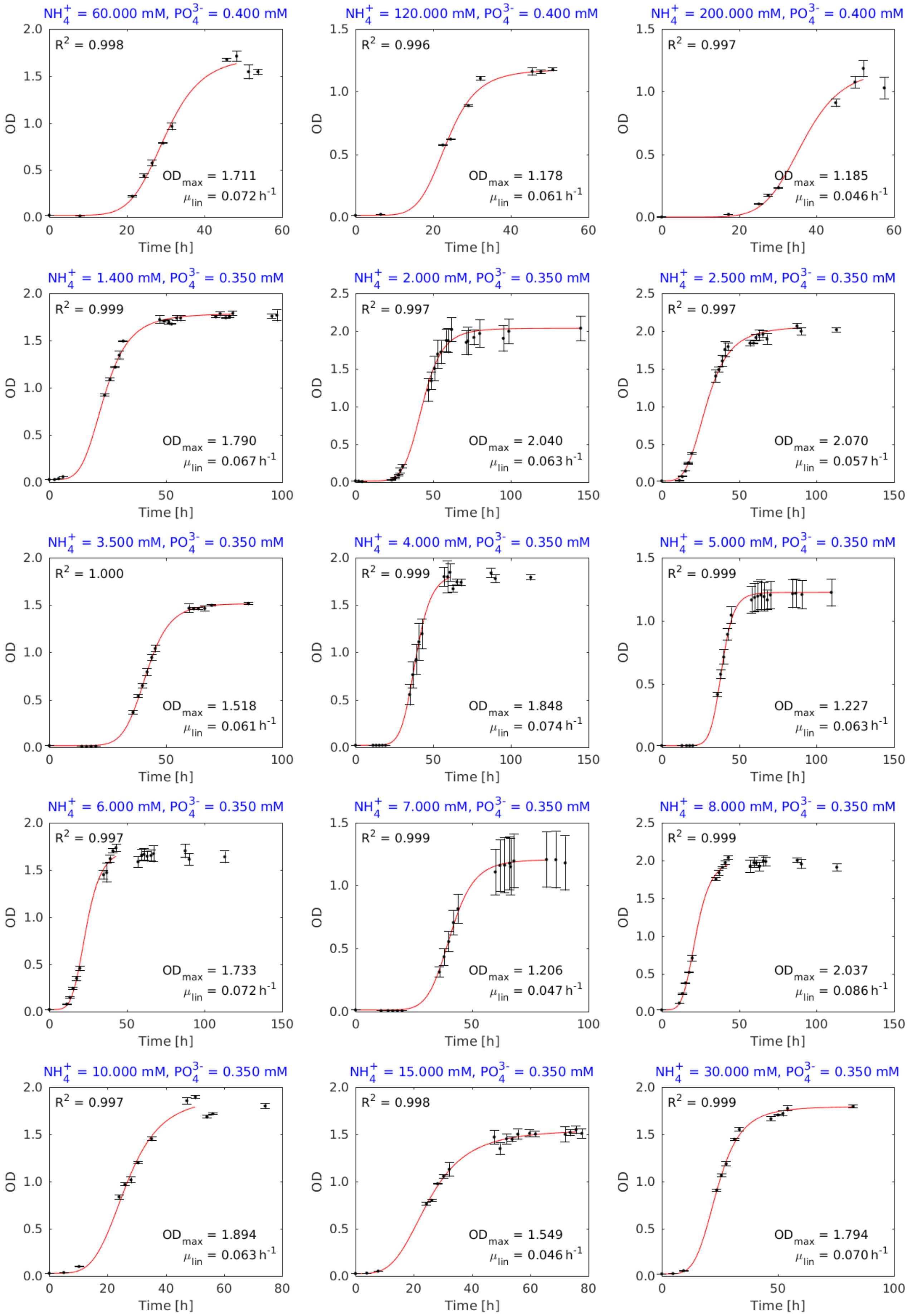
NH_4^+ = 60.000 mM, PO_4^{3-} = 0.400 mM
R^2 = 0.998
OD_{max} = 1.711
μ_{lin} = 0.072 h^{-1}
NH_4^+ = 120.000 mM, PO_4^{3-} = 0.400 mM
R^2 = 0.996
OD_{max} = 1.178
μ_{lin} = 0.061 h^{-1}
NH_4^+ = 200.000 mM, PO_4^{3-} = 0.400 mM
R^2 = 0.997
OD_{max} = 1.185
μ_{lin} = 0.046 h^{-1}
NH_4^+ = 1.400 mM, PO_4^{3-} = 0.350 mM
R^2 = 0.999
OD_{max} = 1.790
μ_{lin} = 0.067 h^{-1}
NH_4^+ = 2.000 mM, PO_4^{3-} = 0.350 mM
R^2 = 0.997
OD_{max} = 2.040
μ_{lin} = 0.063 h^{-1}
NH_4^+ = 2.500 mM, PO_4^{3-} = 0.350 mM
R^2 = 0.997
OD_{max} = 2.070
μ_{lin} = 0.057 h^{-1}
NH_4^+ = 3.500 mM, PO_4^{3-} = 0.350 mM
R^2 = 1.000
OD_{max} = 1.518
μ_{lin} = 0.061 h^{-1}
NH_4^+ = 4.000 mM, PO_4^{3-} = 0.350 mM
R^2 = 0.999
OD_{max} = 1.848
μ_{lin} = 0.074 h^{-1}
NH_4^+ = 5.000 mM, PO_4^{3-} = 0.350 mM
R^2 = 0.999
OD_{max} = 1.227
μ_{lin} = 0.063 h^{-1}
NH_4^+ = 6.000 mM, PO_4^{3-} = 0.350 mM
R^2 = 0.997
OD_{max} = 1.733
μ_{lin} = 0.072 h^{-1}
NH_4^+ = 7.000 mM, PO_4^{3-} = 0.350 mM
R^2 = 0.999
OD_{max} = 1.206
μ_{lin} = 0.047 h^{-1}
NH_4^+ = 8.000 mM, PO_4^{3-} = 0.350 mM
R^2 = 0.999
OD_{max} = 2.037
μ_{lin} = 0.086 h^{-1}
NH_4^+ = 10.000 mM, PO_4^{3-} = 0.350 mM
R^2 = 0.997
OD_{max} = 1.894
μ_{lin} = 0.063 h^{-1}
NH_4^+ = 15.000 mM, PO_4^{3-} = 0.350 mM
R^2 = 0.998
OD_{max} = 1.549
μ_{lin} = 0.046 h^{-1}
NH_4^+ = 30.000 mM, PO_4^{3-} = 0.350 mM
R^2 = 0.999
OD_{max} = 1.794
μ_{lin} = 0.070 h^{-1}
OD
Time [h]

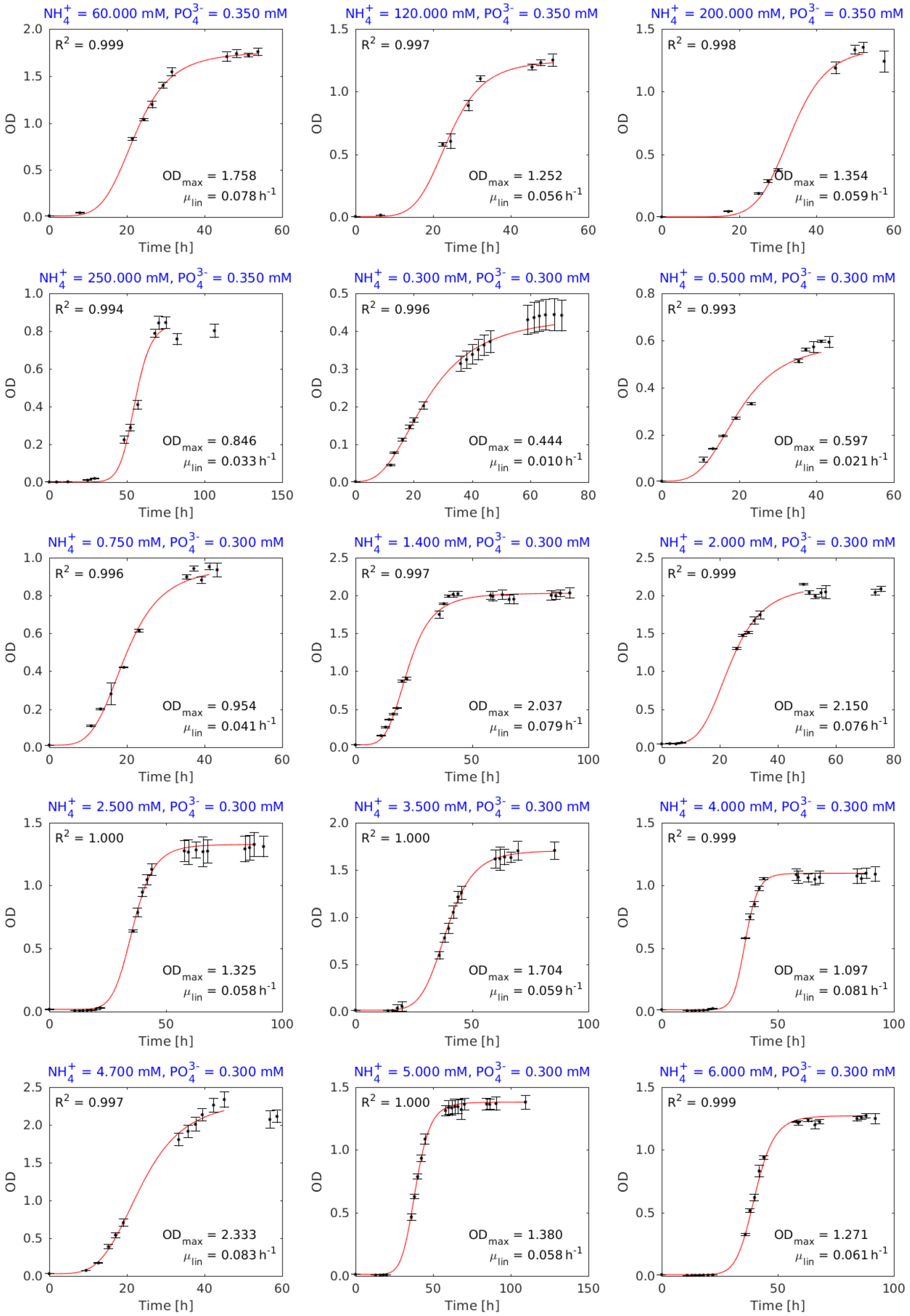
NH4+ = 60.000 mM, PO4 3- = 0.350 mM
R² = 0.999
OD max = 1.758
μ lin = 0.078 h-1
NH4+ = 120.000 mM, PO4 3- = 0.350 mM
R² = 0.997
OD max = 1.252
μ lin = 0.056 h-1
NH4+ = 200.000 mM, PO4 3- = 0.350 mM
R² = 0.998
OD max = 1.354
μ lin = 0.059 h-1
NH4+ = 250.000 mM, PO4 3- = 0.350 mM
R² = 0.994
OD max = 0.846
μ lin = 0.033 h-1
NH4+ = 0.300 mM, PO4 3- = 0.300 mM
R² = 0.996
OD max = 0.444
μ lin = 0.010 h-1
NH4+ = 0.500 mM, PO4 3- = 0.300 mM
R² = 0.993
OD max = 0.597
μ lin = 0.021 h-1
NH4+ = 0.750 mM, PO4 3- = 0.300 mM
R² = 0.996
OD max = 0.954
μ lin = 0.041 h-1
NH4+ = 1.400 mM, PO4 3- = 0.300 mM
R² = 0.997
OD max = 2.037
μ lin = 0.079 h-1
NH4+ = 2.000 mM, PO4 3- = 0.300 mM
R² = 0.999
OD max = 2.150
μ lin = 0.076 h-1
NH4+ = 2.500 mM, PO4 3- = 0.300 mM
R² = 1.000
OD max = 1.325
μ lin = 0.058 h-1
NH4+ = 3.500 mM, PO4 3- = 0.300 mM
R² = 1.000
OD max = 1.704
μ lin = 0.059 h-1
NH4+ = 4.000 mM, PO4 3- = 0.300 mM
R² = 0.999
OD max = 1.097
μ lin = 0.081 h-1
NH4+ = 4.700 mM, PO4 3- = 0.300 mM
R² = 0.997
OD max = 2.333
μ lin = 0.083 h-1
NH4+ = 5.000 mM, PO4 3- = 0.300 mM
R² = 1.000
OD max = 1.380
μ lin = 0.058 h-1
NH4+ = 6.000 mM, PO4 3- = 0.300 mM
R² = 0.999
OD max = 1.271
μ lin = 0.061 h-1
OD
Time [h]

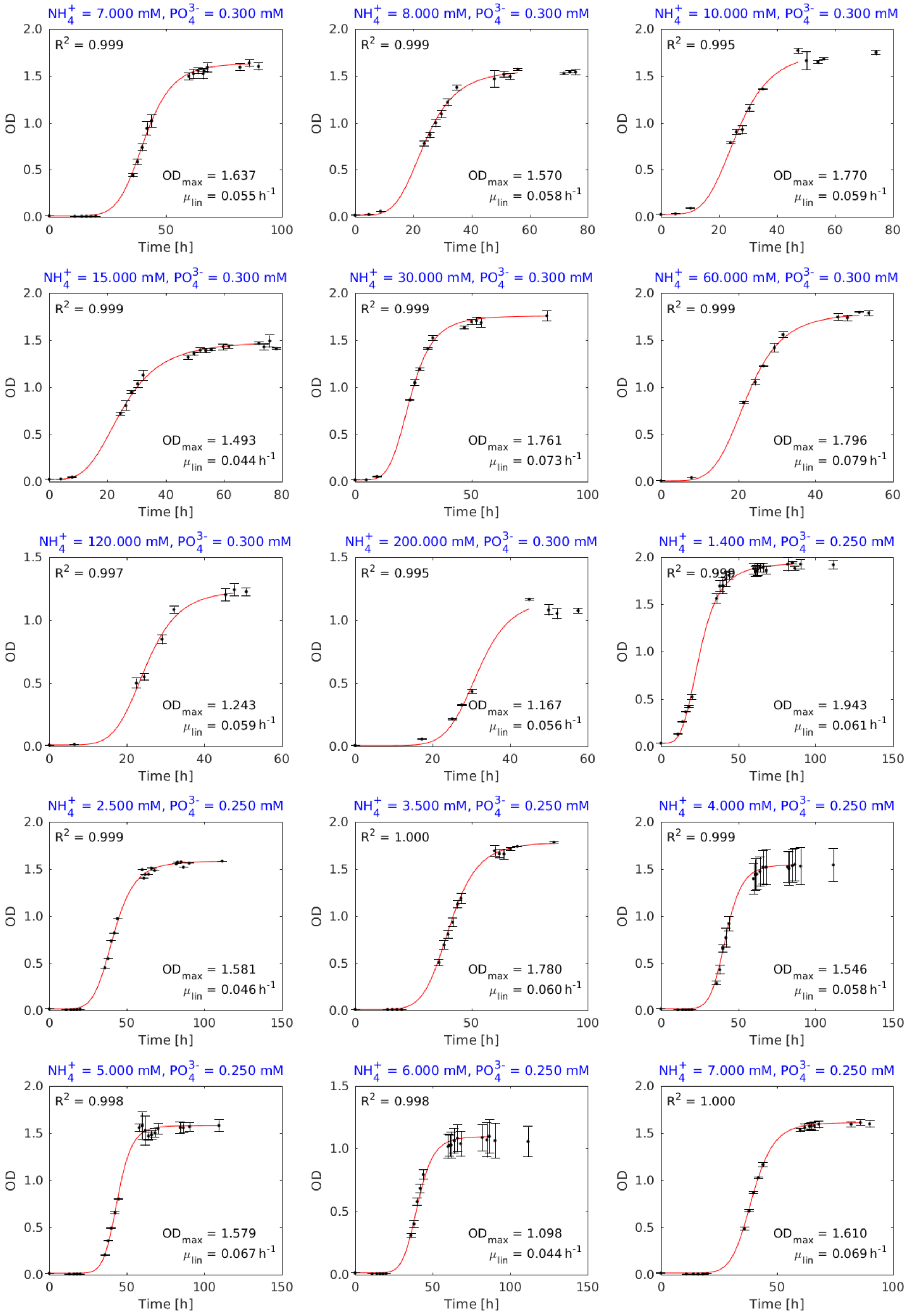
NH4+ = 7.000 mM, PO43- = 0.300 mM
R2 = 0.999
ODmax = 1.637
μlin = 0.055 h-1
NH4+ = 8.000 mM, PO43- = 0.300 mM
R2 = 0.999
ODmax = 1.570
μlin = 0.058 h-1
NH4+ = 10.000 mM, PO43- = 0.300 mM
R2 = 0.995
ODmax = 1.770
μlin = 0.059 h-1
NH4+ = 15.000 mM, PO43- = 0.300 mM
R2 = 0.999
ODmax = 1.493
μlin = 0.044 h-1
NH4+ = 30.000 mM, PO43- = 0.300 mM
R2 = 0.999
ODmax = 1.761
μlin = 0.073 h-1
NH4+ = 60.000 mM, PO43- = 0.300 mM
R2 = 0.999
ODmax = 1.796
μlin = 0.079 h-1
NH4+ = 120.000 mM, PO43- = 0.300 mM
R2 = 0.997
ODmax = 1.243
μlin = 0.059 h-1
NH4+ = 200.000 mM, PO43- = 0.300 mM
R2 = 0.995
ODmax = 1.167
μlin = 0.056 h-1
NH4+ = 1.400 mM, PO43- = 0.250 mM
ODmax = 1.943
μlin = 0.061 h-1
NH4+ = 2.500 mM, PO43- = 0.250 mM
R2 = 0.999
ODmax = 1.581
μlin = 0.046 h-1
NH4+ = 3.500 mM, PO43- = 0.250 mM
R2 = 1.000
ODmax = 1.780
μlin = 0.060 h-1
NH4+ = 4.000 mM, PO43- = 0.250 mM
R2 = 0.999
ODmax = 1.546
μlin = 0.058 h-1
NH4+ = 5.000 mM, PO43- = 0.250 mM
R2 = 0.998
ODmax = 1.579
μlin = 0.067 h-1
NH4+ = 6.000 mM, PO43- = 0.250 mM
R2 = 0.998
ODmax = 1.098
μlin = 0.044 h-1
NH4+ = 7.000 mM, PO43- = 0.250 mM
R2 = 1.000
ODmax = 1.610
μlin = 0.069 h-1
OD
Time [h]

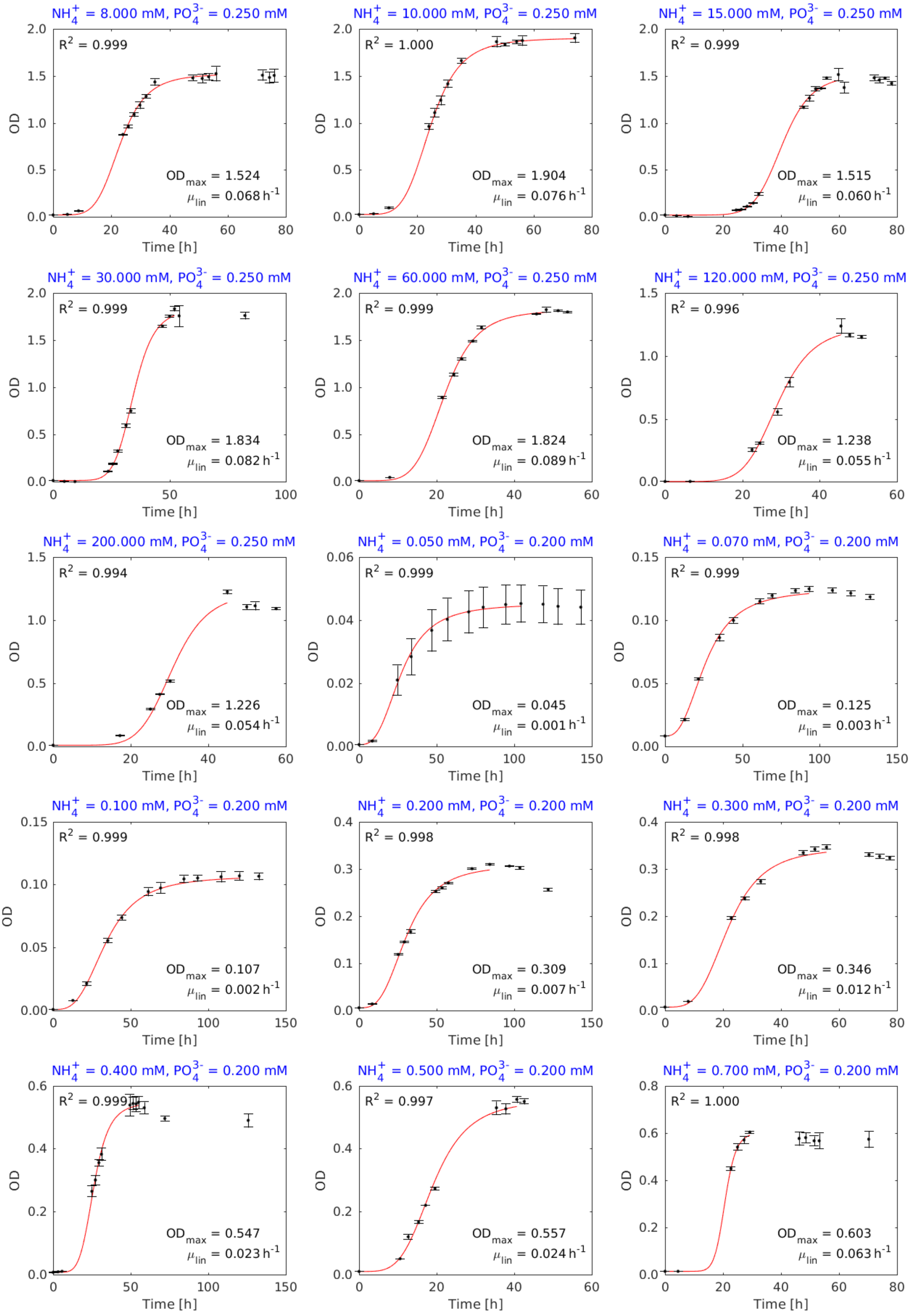
NH4+ = 8.000 mM, PO43- = 0.250 mM
R2 = 0.999
OD max = 1.524
μlin = 0.068 h-1
NH4+ = 10.000 mM, PO43- = 0.250 mM
R2 = 1.000
OD max = 1.904
μlin = 0.076 h-1
NH4+ = 15.000 mM, PO43- = 0.250 mM
R2 = 0.999
OD max = 1.515
μlin = 0.060 h-1
NH4+ = 30.000 mM, PO43- = 0.250 mM
R2 = 0.999
OD max = 1.834
μlin = 0.082 h-1
NH4+ = 60.000 mM, PO43- = 0.250 mM
R2 = 0.999
OD max = 1.824
μlin = 0.089 h-1
NH4+ = 120.000 mM, PO43- = 0.250 mM
R2 = 0.996
OD max = 1.238
μlin = 0.055 h-1
NH4+ = 200.000 mM, PO43- = 0.250 mM
R2 = 0.994
OD max = 1.226
μlin = 0.054 h-1
NH4+ = 0.050 mM, PO43- = 0.200 mM
R2 = 0.999
OD max = 0.045
μlin = 0.001 h-1
NH4+ = 0.070 mM, PO43- = 0.200 mM
R2 = 0.999
OD max = 0.125
μlin = 0.003 h-1
NH4+ = 0.100 mM, PO43- = 0.200 mM
R2 = 0.999
OD max = 0.107
μlin = 0.002 h-1
NH4+ = 0.200 mM, PO43- = 0.200 mM
R2 = 0.998
OD max = 0.309
μlin = 0.007 h-1
NH4+ = 0.300 mM, PO43- = 0.200 mM
R2 = 0.998
OD max = 0.346
μlin = 0.012 h-1
NH4+ = 0.400 mM, PO43- = 0.200 mM
R2 = 0.999
OD max = 0.547
μlin = 0.023 h-1
NH4+ = 0.500 mM, PO43- = 0.200 mM
R2 = 0.997
OD max = 0.557
μlin = 0.024 h-1
NH4+ = 0.700 mM, PO43- = 0.200 mM
R2 = 1.000
OD max = 0.603
μlin = 0.063 h-1
OD
Time [h]

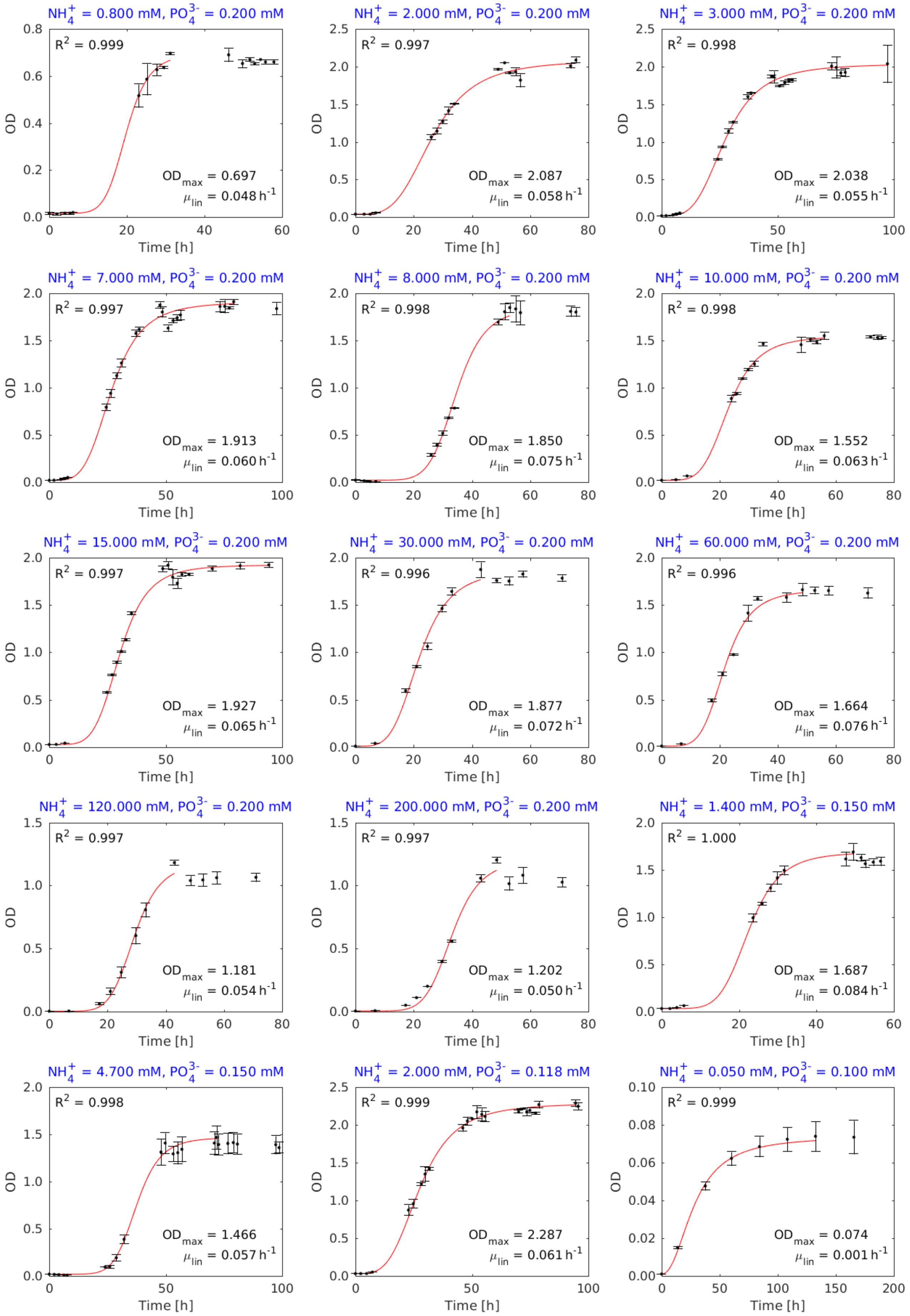
NH4+ = 0.800 mM, PO43- = 0.200 mM
R2 = 0.999
ODmax = 0.697
μlin = 0.048 h-1
NH4+ = 2.000 mM, PO43- = 0.200 mM
R2 = 0.997
ODmax = 2.087
μlin = 0.058 h-1
NH4+ = 3.000 mM, PO43- = 0.200 mM
R2 = 0.998
ODmax = 2.038
μlin = 0.055 h-1
NH4+ = 7.000 mM, PO43- = 0.200 mM
R2 = 0.997
ODmax = 1.913
μlin = 0.060 h-1
NH4+ = 8.000 mM, PO43- = 0.200 mM
R2 = 0.998
ODmax = 1.850
μlin = 0.075 h-1
NH4+ = 10.000 mM, PO43- = 0.200 mM
R2 = 0.998
ODmax = 1.552
μlin = 0.063 h-1
NH4+ = 15.000 mM, PO43- = 0.200 mM
R2 = 0.997
ODmax = 1.927
μlin = 0.065 h-1
NH4+ = 30.000 mM, PO43- = 0.200 mM
R2 = 0.996
ODmax = 1.877
μlin = 0.072 h-1
NH4+ = 60.000 mM, PO43- = 0.200 mM
R2 = 0.996
ODmax = 1.664
μlin = 0.076 h-1
NH4+ = 120.000 mM, PO43- = 0.200 mM
R2 = 0.997
ODmax = 1.181
μlin = 0.054 h-1
NH4+ = 200.000 mM, PO43- = 0.200 mM
R2 = 0.997
ODmax = 1.202
μlin = 0.050 h-1
NH4+ = 1.400 mM, PO43- = 0.150 mM
R2 = 1.000
ODmax = 1.687
μlin = 0.084 h-1
NH4+ = 4.700 mM, PO43- = 0.150 mM
R2 = 0.998
ODmax = 1.466
μlin = 0.057 h-1
NH4+ = 2.000 mM, PO43- = 0.118 mM
R2 = 0.999
ODmax = 2.287
μlin = 0.061 h-1
NH4+ = 0.050 mM, PO43- = 0.100 mM
R2 = 0.999
ODmax = 0.074
μlin = 0.001 h-1
OD
Time [h]

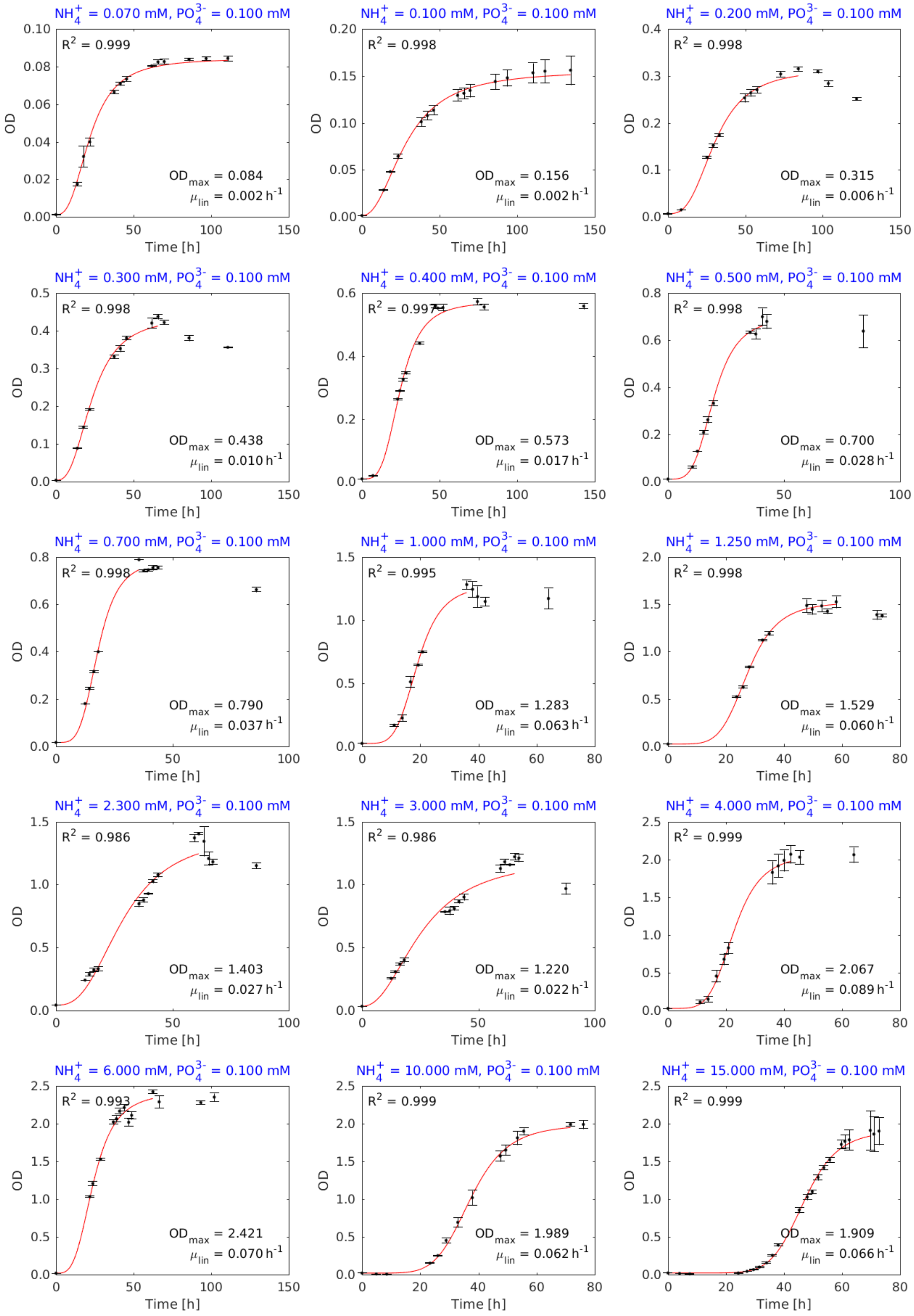
NH4+ = 0.070 mM, PO4 3- = 0.100 mM
R2 = 0.999
ODmax = 0.084
μlin = 0.002 h-1
NH4+ = 0.100 mM, PO4 3- = 0.100 mM
R2 = 0.998
ODmax = 0.156
μlin = 0.002 h-1
NH4+ = 0.200 mM, PO4 3- = 0.100 mM
R2 = 0.998
ODmax = 0.315
μlin = 0.006 h-1
NH4+ = 0.300 mM, PO4 3- = 0.100 mM
R2 = 0.998
ODmax = 0.438
μlin = 0.010 h-1
NH4+ = 0.400 mM, PO4 3- = 0.100 mM
R2 = 0.997
ODmax = 0.573
μlin = 0.017 h-1
NH4+ = 0.500 mM, PO4 3- = 0.100 mM
R2 = 0.998
ODmax = 0.700
μlin = 0.028 h-1
NH4+ = 0.700 mM, PO4 3- = 0.100 mM
R2 = 0.998
ODmax = 0.790
μlin = 0.037 h-1
NH4+ = 1.000 mM, PO4 3- = 0.100 mM
R2 = 0.995
ODmax = 1.283
μlin = 0.063 h-1
NH4+ = 1.250 mM, PO4 3- = 0.100 mM
R2 = 0.998
ODmax = 1.529
μlin = 0.060 h-1
NH4+ = 2.300 mM, PO4 3- = 0.100 mM
R2 = 0.986
ODmax = 1.403
μlin = 0.027 h-1
NH4+ = 3.000 mM, PO4 3- = 0.100 mM
R2 = 0.986
ODmax = 1.220
μlin = 0.022 h-1
NH4+ = 4.000 mM, PO4 3- = 0.100 mM
R2 = 0.999
ODmax = 2.067
μlin = 0.089 h-1
NH4+ = 6.000 mM, PO4 3- = 0.100 mM
R2 = 0.993
ODmax = 2.421
μlin = 0.070 h-1
NH4+ = 10.000 mM, PO4 3- = 0.100 mM
R2 = 0.999
ODmax = 1.989
μlin = 0.062 h-1
NH4+ = 15.000 mM, PO4 3- = 0.100 mM
R2 = 0.999
ODmax = 1.909
μlin = 0.066 h-1
OD
Time [h]

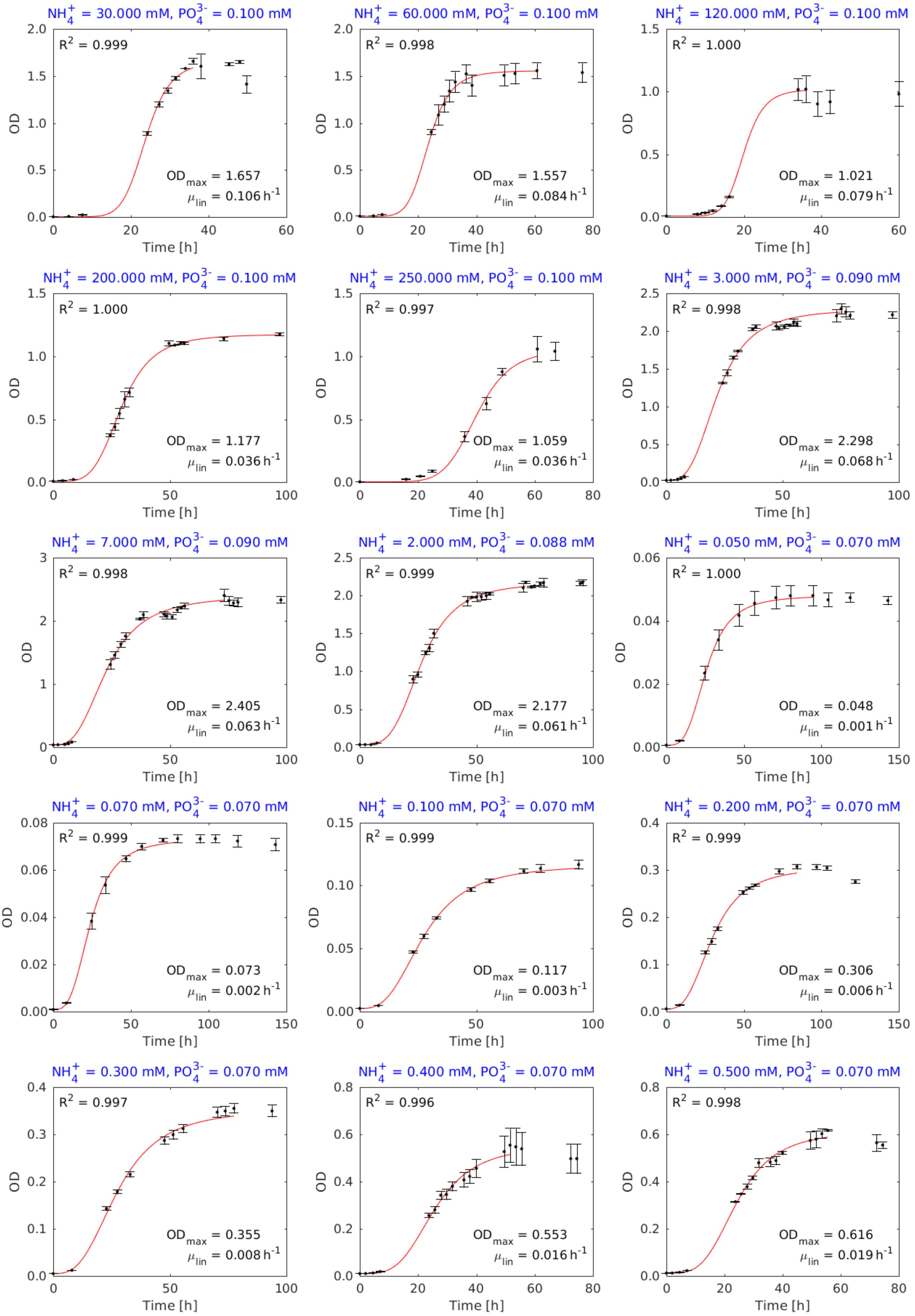

NH_4^+ = 30.000 mM, PO_4^{3-} = 0.100 mM
R^2 = 0.999
OD_{max} = 1.657
μ_{lin} = 0.106 h^{-1}
NH_4^+ = 60.000 mM, PO_4^{3-} = 0.100 mM
R^2 = 0.998
OD_{max} = 1.557
μ_{lin} = 0.084 h^{-1}
NH_4^+ = 120.000 mM, PO_4^{3-} = 0.100 mM
R^2 = 1.000
OD_{max} = 1.021
μ_{lin} = 0.079 h^{-1}
NH_4^+ = 200.000 mM, PO_4^{3-} = 0.100 mM
R^2 = 1.000
OD_{max} = 1.177
μ_{lin} = 0.036 h^{-1}
NH_4^+ = 250.000 mM, PO_4^{3-} = 0.100 mM
R^2 = 0.997
OD_{max} = 1.059
μ_{lin} = 0.036 h^{-1}
NH_4^+ = 3.000 mM, PO_4^{3-} = 0.090 mM
R^2 = 0.998
OD_{max} = 2.298
μ_{lin} = 0.068 h^{-1}
NH_4^+ = 7.000 mM, PO_4^{3-} = 0.090 mM
R^2 = 0.998
OD_{max} = 2.405
μ_{lin} = 0.063 h^{-1}
NH_4^+ = 2.000 mM, PO_4^{3-} = 0.088 mM
R^2 = 0.999
OD_{max} = 2.177
μ_{lin} = 0.061 h^{-1}
NH_4^+ = 0.050 mM, PO_4^{3-} = 0.070 mM
R^2 = 1.000
OD_{max} = 0.048
μ_{lin} = 0.001 h^{-1}
NH_4^+ = 0.070 mM, PO_4^{3-} = 0.070 mM
R^2 = 0.999
OD_{max} = 0.073
μ_{lin} = 0.002 h^{-1}
NH_4^+ = 0.100 mM, PO_4^{3-} = 0.070 mM
R^2 = 0.999
OD_{max} = 0.117
μ_{lin} = 0.003 h^{-1}
NH_4^+ = 0.200 mM, PO_4^{3-} = 0.070 mM
R^2 = 0.999
OD_{max} = 0.306
μ_{lin} = 0.006 h^{-1}
NH_4^+ = 0.300 mM, PO_4^{3-} = 0.070 mM
R^2 = 0.997
OD_{max} = 0.355
μ_{lin} = 0.008 h^{-1}
NH_4^+ = 0.400 mM, PO_4^{3-} = 0.070 mM
R^2 = 0.996
OD_{max} = 0.553
μ_{lin} = 0.016 h^{-1}
NH_4^+ = 0.500 mM, PO_4^{3-} = 0.070 mM
R^2 = 0.998
OD_{max} = 0.616
μ_{lin} = 0.019 h^{-1}
OD
Time [h]

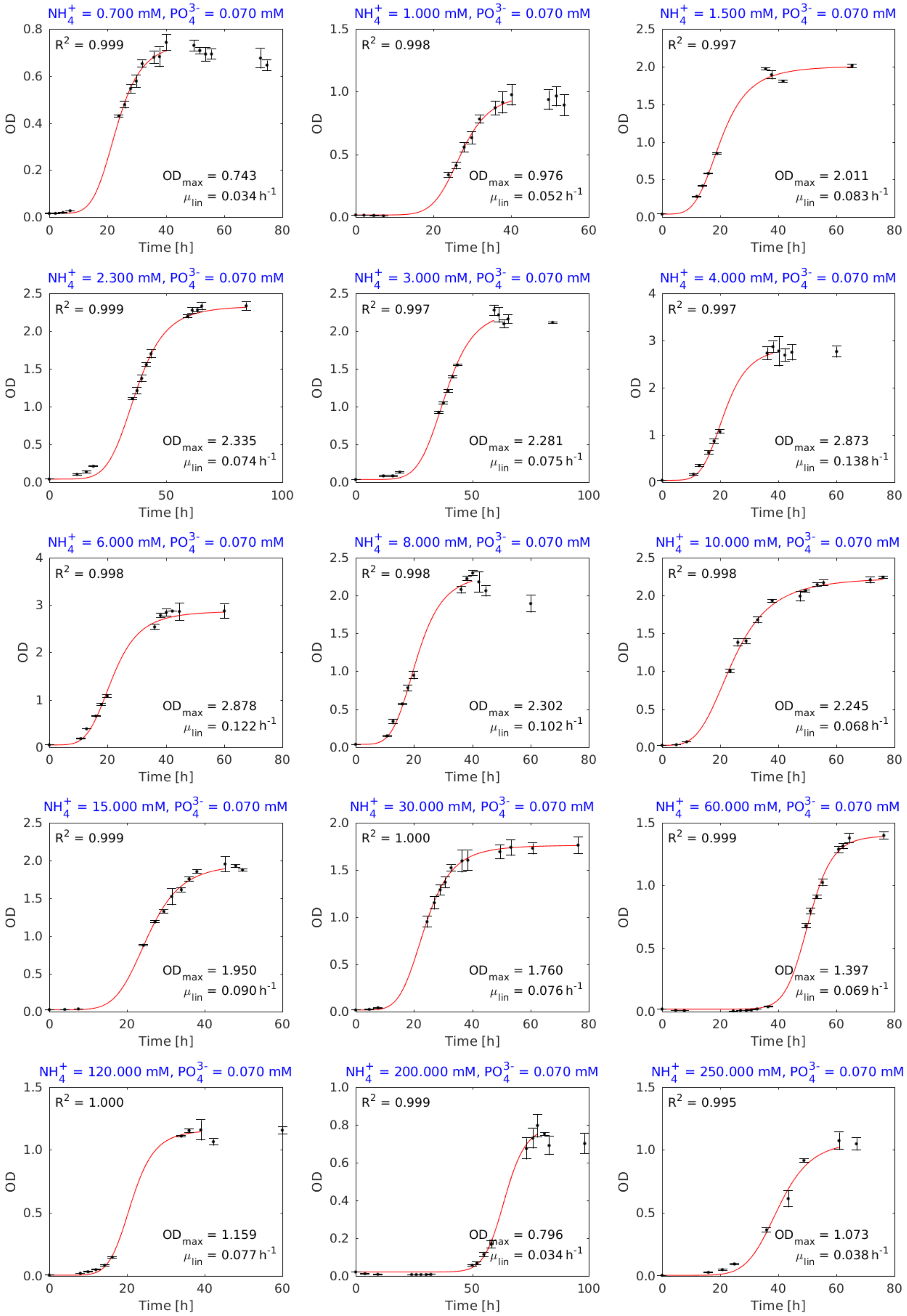

NH_4^+ = 0.700 mM, PO_4^{3-} = 0.070 mM
R^2 = 0.999
OD_{max} = 0.743
μ_{lin} = 0.034 h^{-1}
NH_4^+ = 1.000 mM, PO_4^{3-} = 0.070 mM
R^2 = 0.998
OD_{max} = 0.976
μ_{lin} = 0.052 h^{-1}
NH_4^+ = 1.500 mM, PO_4^{3-} = 0.070 mM
R^2 = 0.997
OD_{max} = 2.011
μ_{lin} = 0.083 h^{-1}
NH_4^+ = 2.300 mM, PO_4^{3-} = 0.070 mM
R^2 = 0.999
OD_{max} = 2.335
μ_{lin} = 0.074 h^{-1}
NH_4^+ = 3.000 mM, PO_4^{3-} = 0.070 mM
R^2 = 0.997
OD_{max} = 2.281
μ_{lin} = 0.075 h^{-1}
NH_4^+ = 4.000 mM, PO_4^{3-} = 0.070 mM
R^2 = 0.997
OD_{max} = 2.873
μ_{lin} = 0.138 h^{-1}
NH_4^+ = 6.000 mM, PO_4^{3-} = 0.070 mM
R^2 = 0.998
OD_{max} = 2.878
μ_{lin} = 0.122 h^{-1}
NH_4^+ = 8.000 mM, PO_4^{3-} = 0.070 mM
R^2 = 0.998
OD_{max} = 2.302
μ_{lin} = 0.102 h^{-1}
NH_4^+ = 10.000 mM, PO_4^{3-} = 0.070 mM
R^2 = 0.998
OD_{max} = 2.245
μ_{lin} = 0.068 h^{-1}
NH_4^+ = 15.000 mM, PO_4^{3-} = 0.070 mM
R^2 = 0.999
OD_{max} = 1.950
μ_{lin} = 0.090 h^{-1}
NH_4^+ = 30.000 mM, PO_4^{3-} = 0.070 mM
R^2 = 1.000
OD_{max} = 1.760
μ_{lin} = 0.076 h^{-1}
NH_4^+ = 60.000 mM, PO_4^{3-} = 0.070 mM
R^2 = 0.999
OD_{max} = 1.397
μ_{lin} = 0.069 h^{-1}
NH_4^+ = 120.000 mM, PO_4^{3-} = 0.070 mM
R^2 = 1.000
OD_{max} = 1.159
μ_{lin} = 0.077 h^{-1}
NH_4^+ = 200.000 mM, PO_4^{3-} = 0.070 mM
R^2 = 0.999
OD_{max} = 0.796
μ_{lin} = 0.034 h^{-1}
NH_4^+ = 250.000 mM, PO_4^{3-} = 0.070 mM
R^2 = 0.995
OD_{max} = 1.073
μ_{lin} = 0.038 h^{-1}
OD
Time [h]

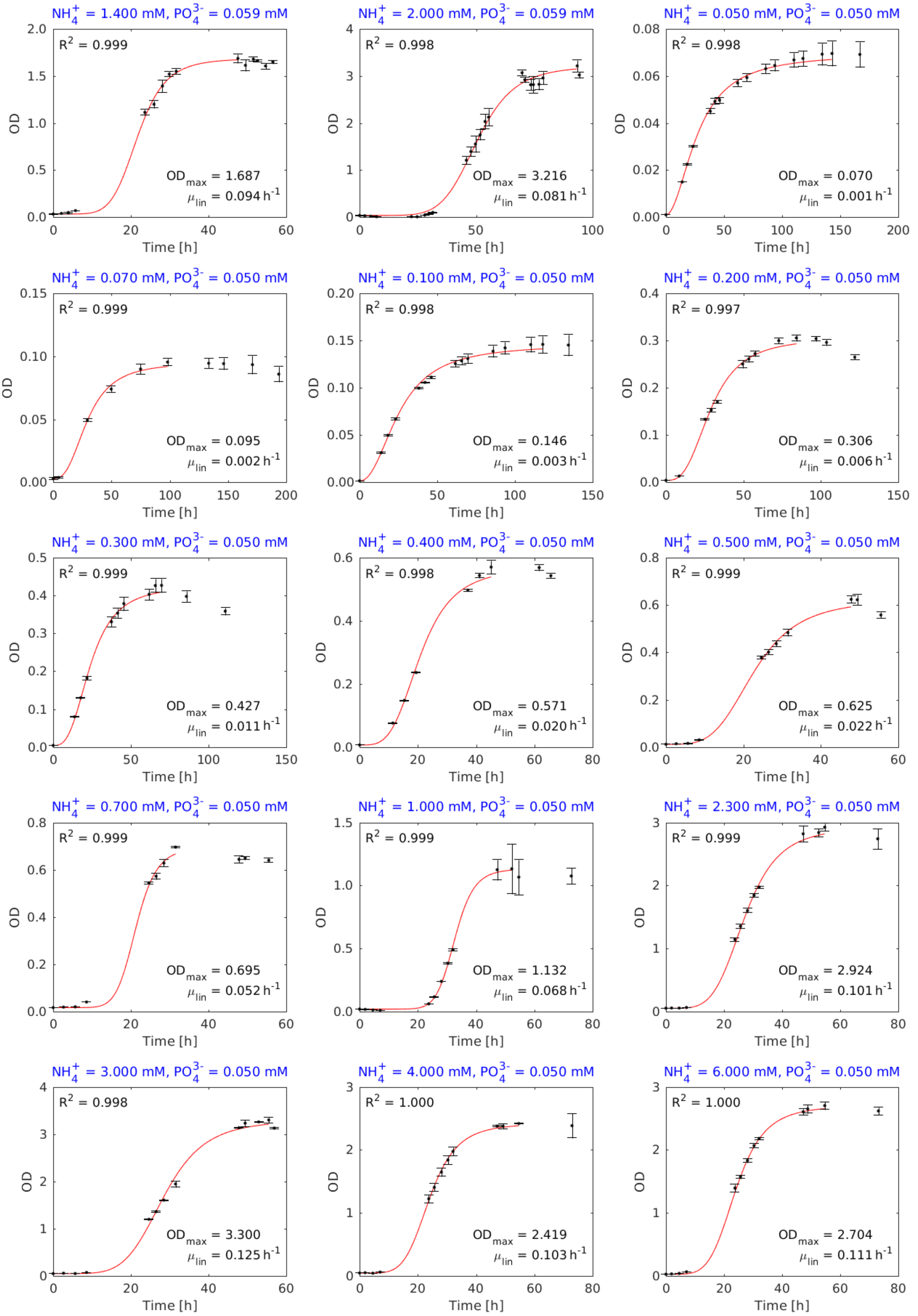

NH4+ = 1.400 mM, PO43- = 0.059 mM
R2 = 0.999
ODmax = 1.687
μlin = 0.094 h-1
NH4+ = 2.000 mM, PO43- = 0.059 mM
R2 = 0.998
ODmax = 3.216
μlin = 0.081 h-1
NH4+ = 0.050 mM, PO43- = 0.050 mM
R2 = 0.998
ODmax = 0.070
μlin = 0.001 h-1
NH4+ = 0.070 mM, PO43- = 0.050 mM
R2 = 0.999
ODmax = 0.095
μlin = 0.002 h-1
NH4+ = 0.100 mM, PO43- = 0.050 mM
R2 = 0.998
ODmax = 0.146
μlin = 0.003 h-1
NH4+ = 0.200 mM, PO43- = 0.050 mM
R2 = 0.997
ODmax = 0.306
μlin = 0.006 h-1
NH4+ = 0.300 mM, PO43- = 0.050 mM
R2 = 0.999
ODmax = 0.427
μlin = 0.011 h-1
NH4+ = 0.400 mM, PO43- = 0.050 mM
R2 = 0.998
ODmax = 0.571
μlin = 0.020 h-1
NH4+ = 0.500 mM, PO43- = 0.050 mM
R2 = 0.999
ODmax = 0.625
μlin = 0.022 h-1
NH4+ = 0.700 mM, PO43- = 0.050 mM
R2 = 0.999
ODmax = 0.695
μlin = 0.052 h-1
NH4+ = 1.000 mM, PO43- = 0.050 mM
R2 = 0.999
ODmax = 1.132
μlin = 0.068 h-1
NH4+ = 2.300 mM, PO43- = 0.050 mM
R2 = 0.999
ODmax = 2.924
μlin = 0.101 h-1
NH4+ = 3.000 mM, PO43- = 0.050 mM
R2 = 0.998
ODmax = 3.300
μlin = 0.125 h-1
NH4+ = 4.000 mM, PO43- = 0.050 mM
R2 = 1.000
ODmax = 2.419
μlin = 0.103 h-1
NH4+ = 6.000 mM, PO43- = 0.050 mM
R2 = 1.000
ODmax = 2.704
μlin = 0.111 h-1
OD
Time [h]

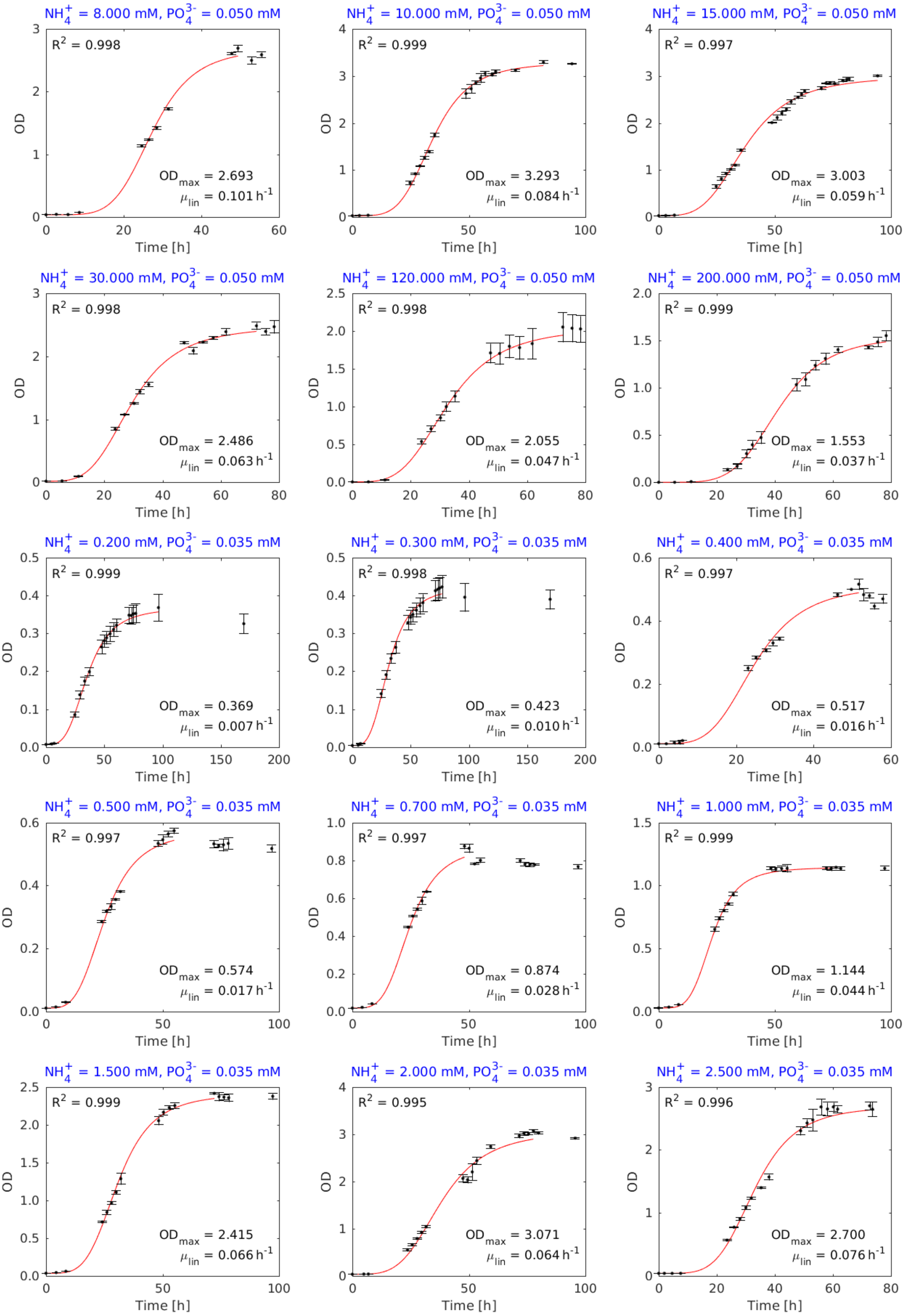

NH4+ = 8.000 mM, PO43- = 0.050 mM
R2 = 0.998
ODmax = 2.693
μlin = 0.101 h-1
NH4+ = 10.000 mM, PO43- = 0.050 mM
R2 = 0.999
ODmax = 3.293
μlin = 0.084 h-1
NH4+ = 15.000 mM, PO43- = 0.050 mM
R2 = 0.997
ODmax = 3.003
μlin = 0.059 h-1
NH4+ = 30.000 mM, PO43- = 0.050 mM
R2 = 0.998
ODmax = 2.486
μlin = 0.063 h-1
NH4+ = 120.000 mM, PO43- = 0.050 mM
R2 = 0.998
ODmax = 2.055
μlin = 0.047 h-1
NH4+ = 200.000 mM, PO43- = 0.050 mM
R2 = 0.999
ODmax = 1.553
μlin = 0.037 h-1
NH4+ = 0.200 mM, PO43- = 0.035 mM
R2 = 0.999
ODmax = 0.369
μlin = 0.007 h-1
NH4+ = 0.300 mM, PO43- = 0.035 mM
R2 = 0.998
ODmax = 0.423
μlin = 0.010 h-1
NH4+ = 0.400 mM, PO43- = 0.035 mM
R2 = 0.997
ODmax = 0.517
μlin = 0.016 h-1
NH4+ = 0.500 mM, PO43- = 0.035 mM
R2 = 0.997
ODmax = 0.574
μlin = 0.017 h-1
NH4+ = 0.700 mM, PO43- = 0.035 mM
R2 = 0.997
ODmax = 0.874
μlin = 0.028 h-1
NH4+ = 1.000 mM, PO43- = 0.035 mM
R2 = 0.999
ODmax = 1.144
μlin = 0.044 h-1
NH4+ = 1.500 mM, PO43- = 0.035 mM
R2 = 0.999
ODmax = 2.415
μlin = 0.066 h-1
NH4+ = 2.000 mM, PO43- = 0.035 mM
R2 = 0.995
ODmax = 3.071
μlin = 0.064 h-1
NH4+ = 2.500 mM, PO43- = 0.035 mM
R2 = 0.996
ODmax = 2.700
μlin = 0.076 h-1
OD
Time [h]

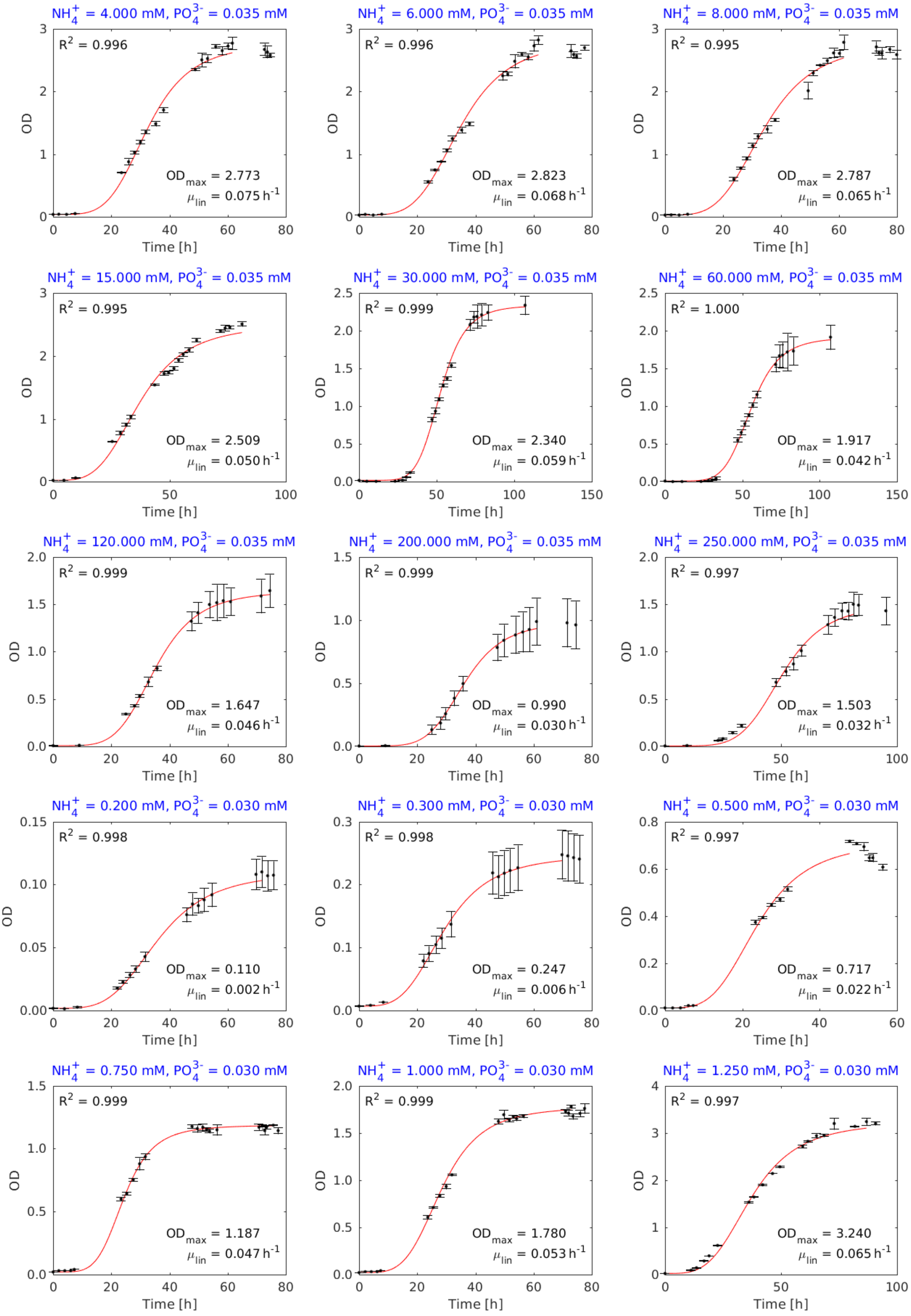
NH4+ = 4.000 mM, PO43- = 0.035 mM
R2 = 0.996
ODmax = 2.773
μlin = 0.075 h-1
NH4+ = 6.000 mM, PO43- = 0.035 mM
R2 = 0.996
ODmax = 2.823
μlin = 0.068 h-1
NH4+ = 8.000 mM, PO43- = 0.035 mM
R2 = 0.995
ODmax = 2.787
μlin = 0.065 h-1
NH4+ = 15.000 mM, PO43- = 0.035 mM
R2 = 0.995
ODmax = 2.509
μlin = 0.050 h-1
NH4+ = 30.000 mM, PO43- = 0.035 mM
R2 = 0.999
ODmax = 2.340
μlin = 0.059 h-1
NH4+ = 60.000 mM, PO43- = 0.035 mM
R2 = 1.000
ODmax = 1.917
μlin = 0.042 h-1
NH4+ = 120.000 mM, PO43- = 0.035 mM
R2 = 0.999
ODmax = 1.647
μlin = 0.046 h-1
NH4+ = 200.000 mM, PO43- = 0.035 mM
R2 = 0.999
ODmax = 0.990
μlin = 0.030 h-1
NH4+ = 250.000 mM, PO43- = 0.035 mM
R2 = 0.997
ODmax = 1.503
μlin = 0.032 h-1
NH4+ = 0.200 mM, PO43- = 0.030 mM
R2 = 0.998
ODmax = 0.110
μlin = 0.002 h-1
NH4+ = 0.300 mM, PO43- = 0.030 mM
R2 = 0.998
ODmax = 0.247
μlin = 0.006 h-1
NH4+ = 0.500 mM, PO43- = 0.030 mM
R2 = 0.997
ODmax = 0.717
μlin = 0.022 h-1
NH4+ = 0.750 mM, PO43- = 0.030 mM
R2 = 0.999
ODmax = 1.187
μlin = 0.047 h-1
NH4+ = 1.000 mM, PO43- = 0.030 mM
R2 = 0.999
ODmax = 1.780
μlin = 0.053 h-1
NH4+ = 1.250 mM, PO43- = 0.030 mM
R2 = 0.997
ODmax = 3.240
μlin = 0.065 h-1
OD
Time [h]

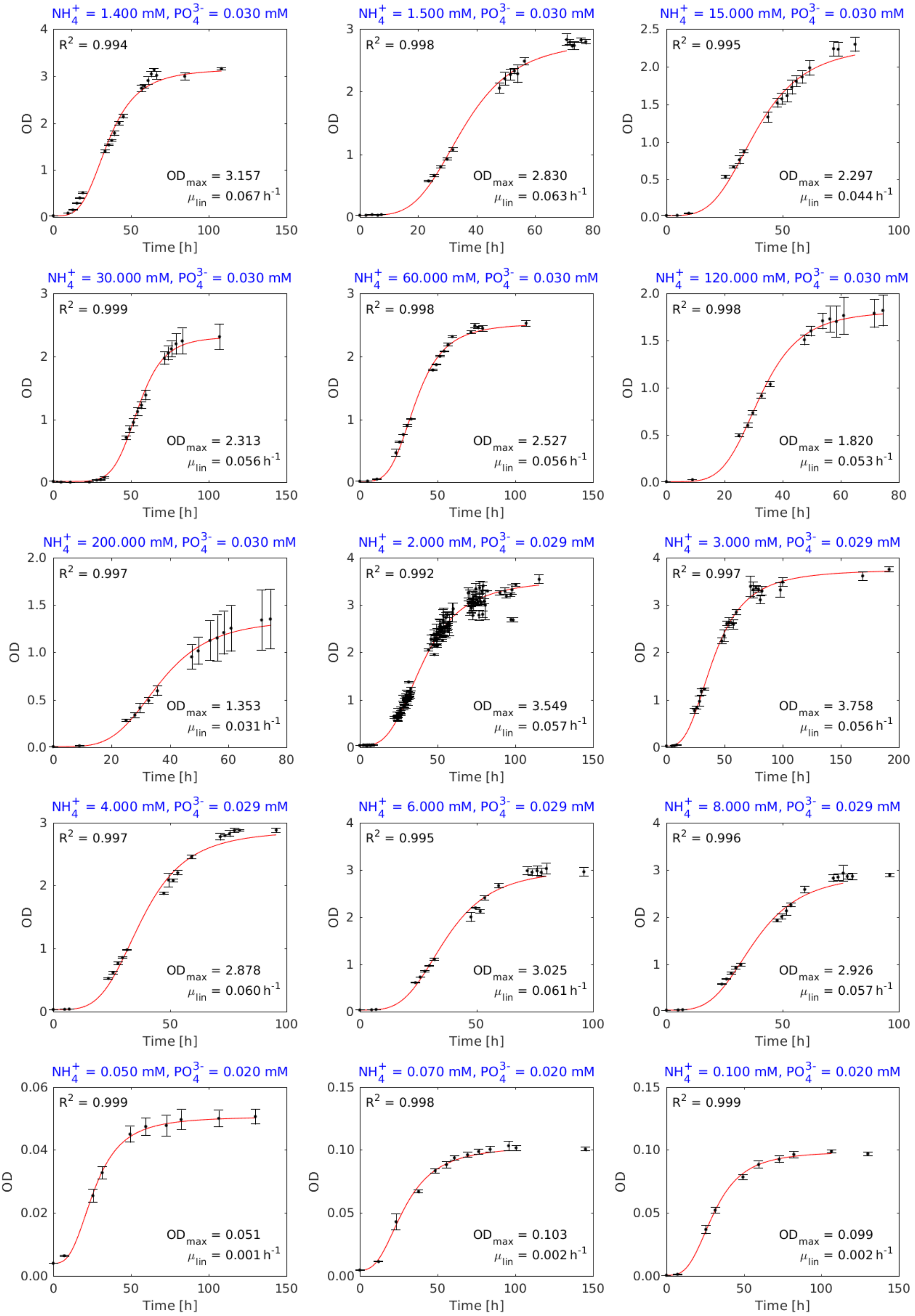

NH4+ = 1.400 mM, PO43- = 0.030 mM
R2 = 0.994
ODmax = 3.157
μlin = 0.067 h-1
NH4+ = 1.500 mM, PO43- = 0.030 mM
R2 = 0.998
ODmax = 2.830
μlin = 0.063 h-1
NH4+ = 15.000 mM, PO43- = 0.030 mM
R2 = 0.995
ODmax = 2.297
μlin = 0.044 h-1
NH4+ = 30.000 mM, PO43- = 0.030 mM
R2 = 0.999
ODmax = 2.313
μlin = 0.056 h-1
NH4+ = 60.000 mM, PO43- = 0.030 mM
R2 = 0.998
ODmax = 2.527
μlin = 0.056 h-1
NH4+ = 120.000 mM, PO43- = 0.030 mM
R2 = 0.998
ODmax = 1.820
μlin = 0.053 h-1
NH4+ = 200.000 mM, PO43- = 0.030 mM
R2 = 0.997
ODmax = 1.353
μlin = 0.031 h-1
NH4+ = 2.000 mM, PO43- = 0.029 mM
R2 = 0.992
ODmax = 3.549
μlin = 0.057 h-1
NH4+ = 3.000 mM, PO43- = 0.029 mM
R2 = 0.997
ODmax = 3.758
μlin = 0.056 h-1
NH4+ = 4.000 mM, PO43- = 0.029 mM
R2 = 0.997
ODmax = 2.878
μlin = 0.060 h-1
NH4+ = 6.000 mM, PO43- = 0.029 mM
R2 = 0.995
ODmax = 3.025
μlin = 0.061 h-1
NH4+ = 8.000 mM, PO43- = 0.029 mM
R2 = 0.996
ODmax = 2.926
μlin = 0.057 h-1
NH4+ = 0.050 mM, PO43- = 0.020 mM
R2 = 0.999
ODmax = 0.051
μlin = 0.001 h-1
NH4+ = 0.070 mM, PO43- = 0.020 mM
R2 = 0.998
ODmax = 0.103
μlin = 0.002 h-1
NH4+ = 0.100 mM, PO43- = 0.020 mM
R2 = 0.999
ODmax = 0.099
μlin = 0.002 h-1
OD
Time [h]

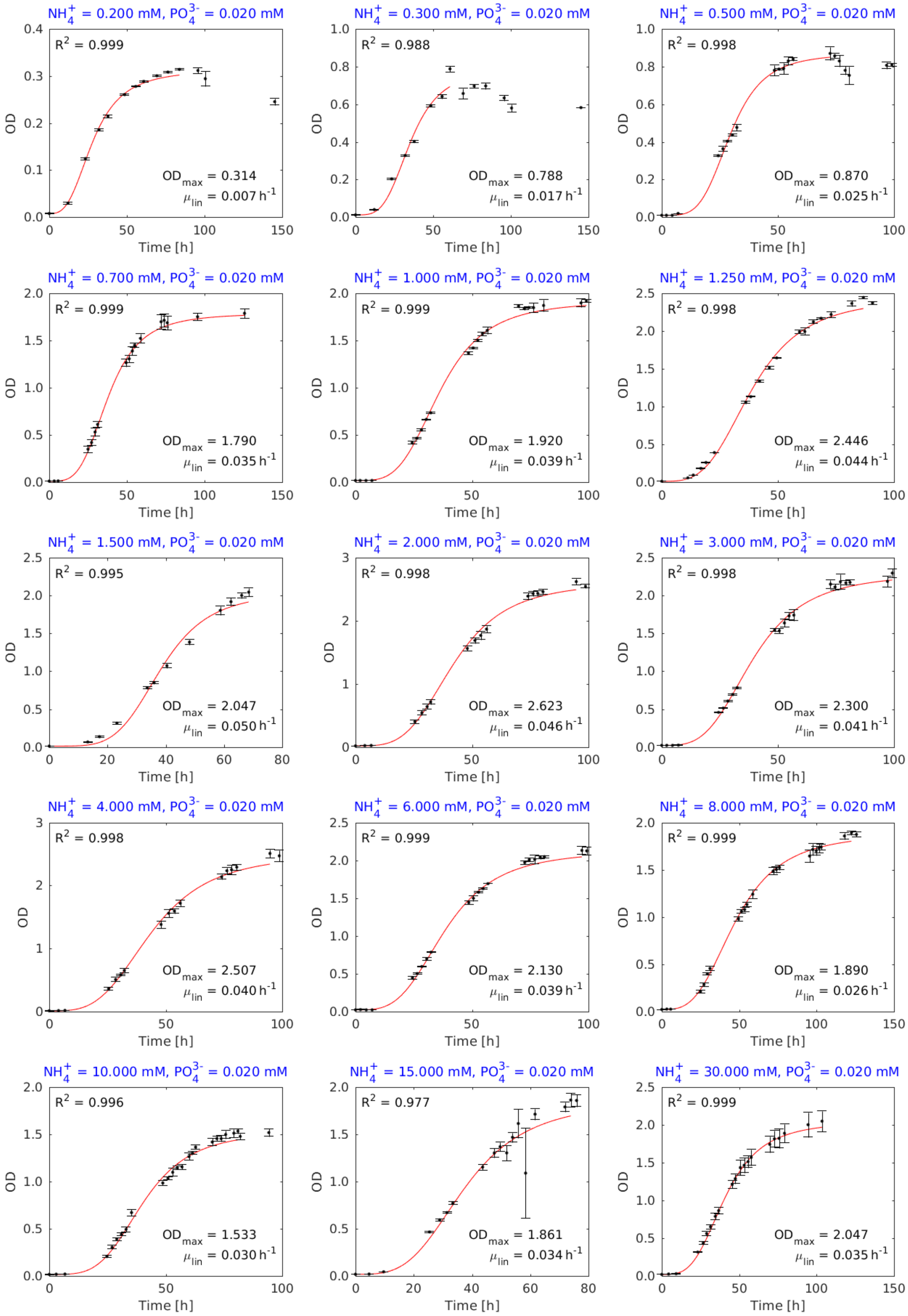
NH4+ = 0.200 mM, PO43- = 0.020 mM
R2 = 0.999
ODmax = 0.314
μlin = 0.007 h-1
NH4+ = 0.300 mM, PO43- = 0.020 mM
R2 = 0.988
ODmax = 0.788
μlin = 0.017 h-1
NH4+ = 0.500 mM, PO43- = 0.020 mM
R2 = 0.998
ODmax = 0.870
μlin = 0.025 h-1
NH4+ = 0.700 mM, PO43- = 0.020 mM
R2 = 0.999
ODmax = 1.790
μlin = 0.035 h-1
NH4+ = 1.000 mM, PO43- = 0.020 mM
R2 = 0.999
ODmax = 1.920
μlin = 0.039 h-1
NH4+ = 1.250 mM, PO43- = 0.020 mM
R2 = 0.998
ODmax = 2.446
μlin = 0.044 h-1
NH4+ = 1.500 mM, PO43- = 0.020 mM
R2 = 0.995
ODmax = 2.047
μlin = 0.050 h-1
NH4+ = 2.000 mM, PO43- = 0.020 mM
R2 = 0.998
ODmax = 2.623
μlin = 0.046 h-1
NH4+ = 3.000 mM, PO43- = 0.020 mM
R2 = 0.998
ODmax = 2.300
μlin = 0.041 h-1
NH4+ = 4.000 mM, PO43- = 0.020 mM
R2 = 0.998
ODmax = 2.507
μlin = 0.040 h-1
NH4+ = 6.000 mM, PO43- = 0.020 mM
R2 = 0.999
ODmax = 2.130
μlin = 0.039 h-1
NH4+ = 8.000 mM, PO43- = 0.020 mM
R2 = 0.999
ODmax = 1.890
μlin = 0.026 h-1
NH4+ = 10.000 mM, PO43- = 0.020 mM
R2 = 0.996
ODmax = 1.533
μlin = 0.030 h-1
NH4+ = 15.000 mM, PO43- = 0.020 mM
R2 = 0.977
ODmax = 1.861
μlin = 0.034 h-1
NH4+ = 30.000 mM, PO43- = 0.020 mM
R2 = 0.999
ODmax = 2.047
μlin = 0.035 h-1
OD
Time [h]

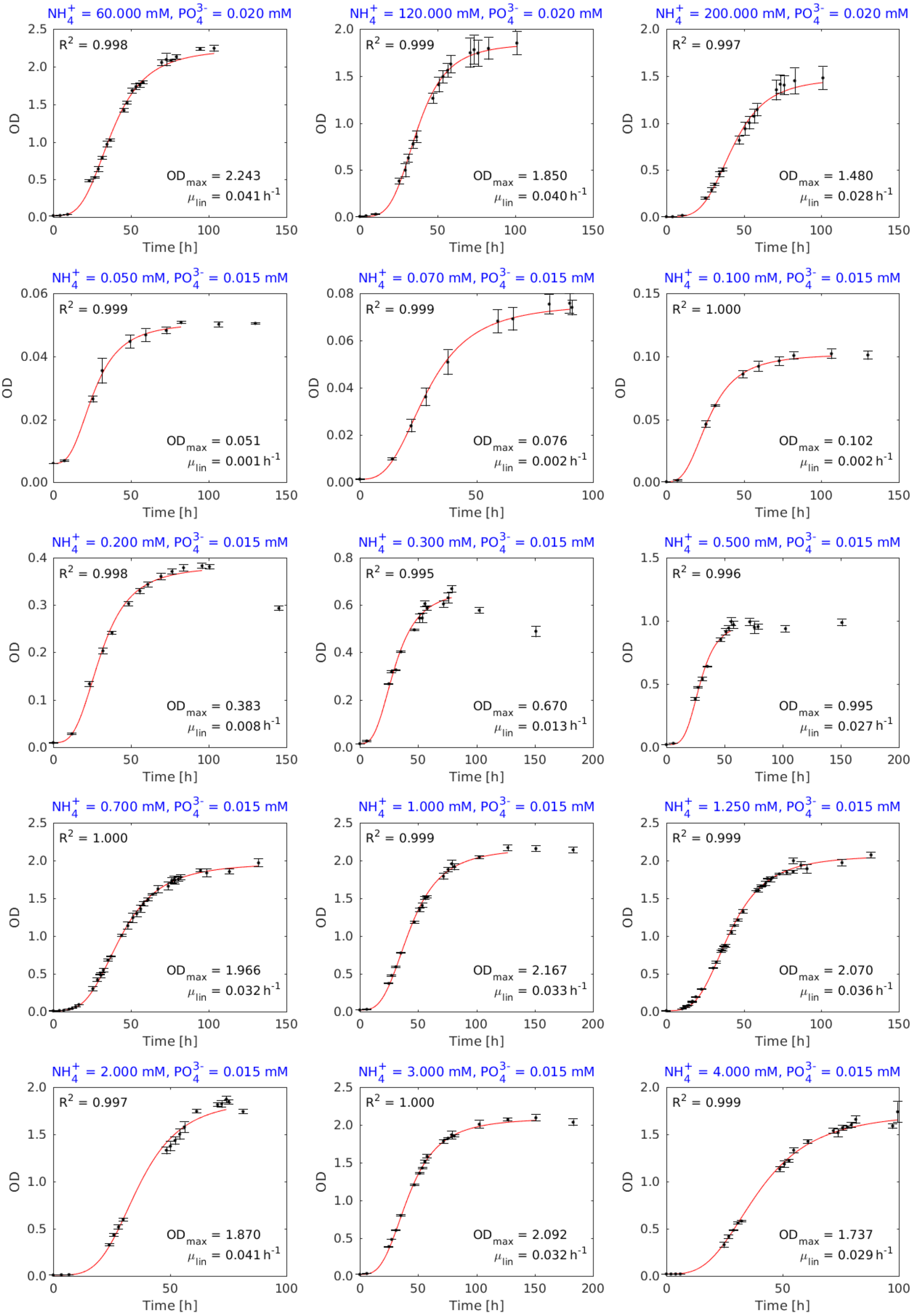
NH4+ = 60.000 mM, PO43- = 0.020 mM
R2 = 0.998
ODmax = 2.243
μlin = 0.041 h-1
NH4+ = 120.000 mM, PO43- = 0.020 mM
R2 = 0.999
ODmax = 1.850
μlin = 0.040 h-1
NH4+ = 200.000 mM, PO43- = 0.020 mM
R2 = 0.997
ODmax = 1.480
μlin = 0.028 h-1
NH4+ = 0.050 mM, PO43- = 0.015 mM
R2 = 0.999
ODmax = 0.051
μlin = 0.001 h-1
NH4+ = 0.070 mM, PO43- = 0.015 mM
R2 = 0.999
ODmax = 0.076
μlin = 0.002 h-1
NH4+ = 0.100 mM, PO43- = 0.015 mM
R2 = 1.000
ODmax = 0.102
μlin = 0.002 h-1
NH4+ = 0.200 mM, PO43- = 0.015 mM
R2 = 0.998
ODmax = 0.383
μlin = 0.008 h-1
NH4+ = 0.300 mM, PO43- = 0.015 mM
R2 = 0.995
ODmax = 0.670
μlin = 0.013 h-1
NH4+ = 0.500 mM, PO43- = 0.015 mM
R2 = 0.996
ODmax = 0.995
μlin = 0.027 h-1
NH4+ = 0.700 mM, PO43- = 0.015 mM
R2 = 1.000
ODmax = 1.966
μlin = 0.032 h-1
NH4+ = 1.000 mM, PO43- = 0.015 mM
R2 = 0.999
ODmax = 2.167
μlin = 0.033 h-1
NH4+ = 1.250 mM, PO43- = 0.015 mM
R2 = 0.999
ODmax = 2.070
μlin = 0.036 h-1
NH4+ = 2.000 mM, PO43- = 0.015 mM
R2 = 0.997
ODmax = 1.870
μlin = 0.041 h-1
NH4+ = 3.000 mM, PO43- = 0.015 mM
R2 = 1.000
ODmax = 2.092
μlin = 0.032 h-1
NH4+ = 4.000 mM, PO43- = 0.015 mM
R2 = 0.999
ODmax = 1.737
μlin = 0.029 h-1
OD
Time [h]

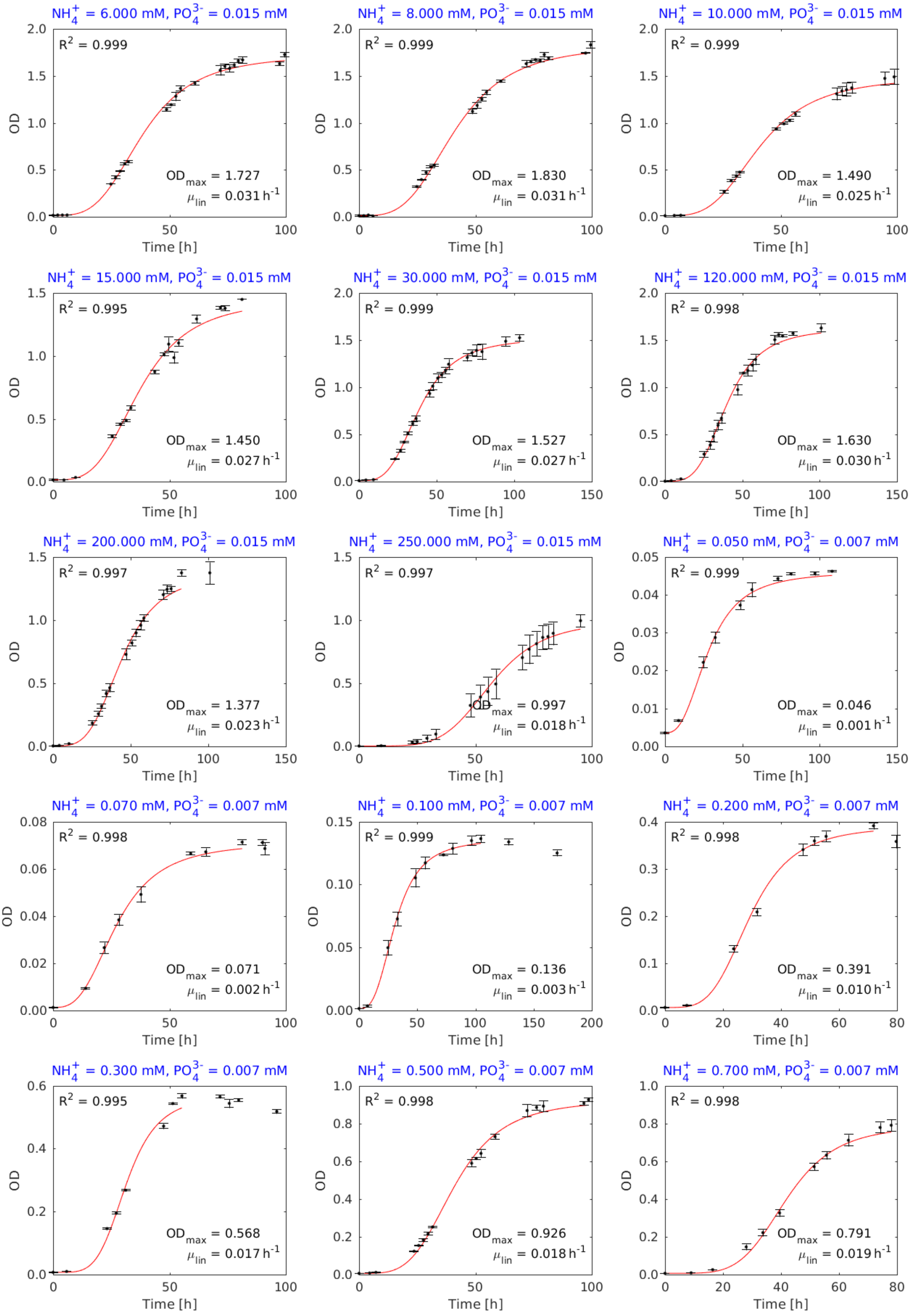
NH4+ = 6.000 mM, PO43- = 0.015 mM
R2 = 0.999
ODmax = 1.727
μlin = 0.031 h-1
NH4+ = 8.000 mM, PO43- = 0.015 mM
R2 = 0.999
ODmax = 1.830
μlin = 0.031 h-1
NH4+ = 10.000 mM, PO43- = 0.015 mM
R2 = 0.999
ODmax = 1.490
μlin = 0.025 h-1
NH4+ = 15.000 mM, PO43- = 0.015 mM
R2 = 0.995
ODmax = 1.450
μlin = 0.027 h-1
NH4+ = 30.000 mM, PO43- = 0.015 mM
R2 = 0.999
ODmax = 1.527
μlin = 0.027 h-1
NH4+ = 120.000 mM, PO43- = 0.015 mM
R2 = 0.998
ODmax = 1.630
μlin = 0.030 h-1
NH4+ = 200.000 mM, PO43- = 0.015 mM
R2 = 0.997
ODmax = 1.377
μlin = 0.023 h-1
NH4+ = 250.000 mM, PO43- = 0.015 mM
R2 = 0.997
ODmax = 0.997
μlin = 0.018 h-1
NH4+ = 0.050 mM, PO43- = 0.007 mM
R2 = 0.999
ODmax = 0.046
μlin = 0.001 h-1
NH4+ = 0.070 mM, PO43- = 0.007 mM
R2 = 0.998
ODmax = 0.071
μlin = 0.002 h-1
NH4+ = 0.100 mM, PO43- = 0.007 mM
R2 = 0.999
ODmax = 0.136
μlin = 0.003 h-1
NH4+ = 0.200 mM, PO43- = 0.007 mM
R2 = 0.998
ODmax = 0.391
μlin = 0.010 h-1
NH4+ = 0.300 mM, PO43- = 0.007 mM
R2 = 0.995
ODmax = 0.568
μlin = 0.017 h-1
NH4+ = 0.500 mM, PO43- = 0.007 mM
R2 = 0.998
ODmax = 0.926
μlin = 0.018 h-1
NH4+ = 0.700 mM, PO43- = 0.007 mM
R2 = 0.998
ODmax = 0.791
μlin = 0.019 h-1
OD
Time [h]

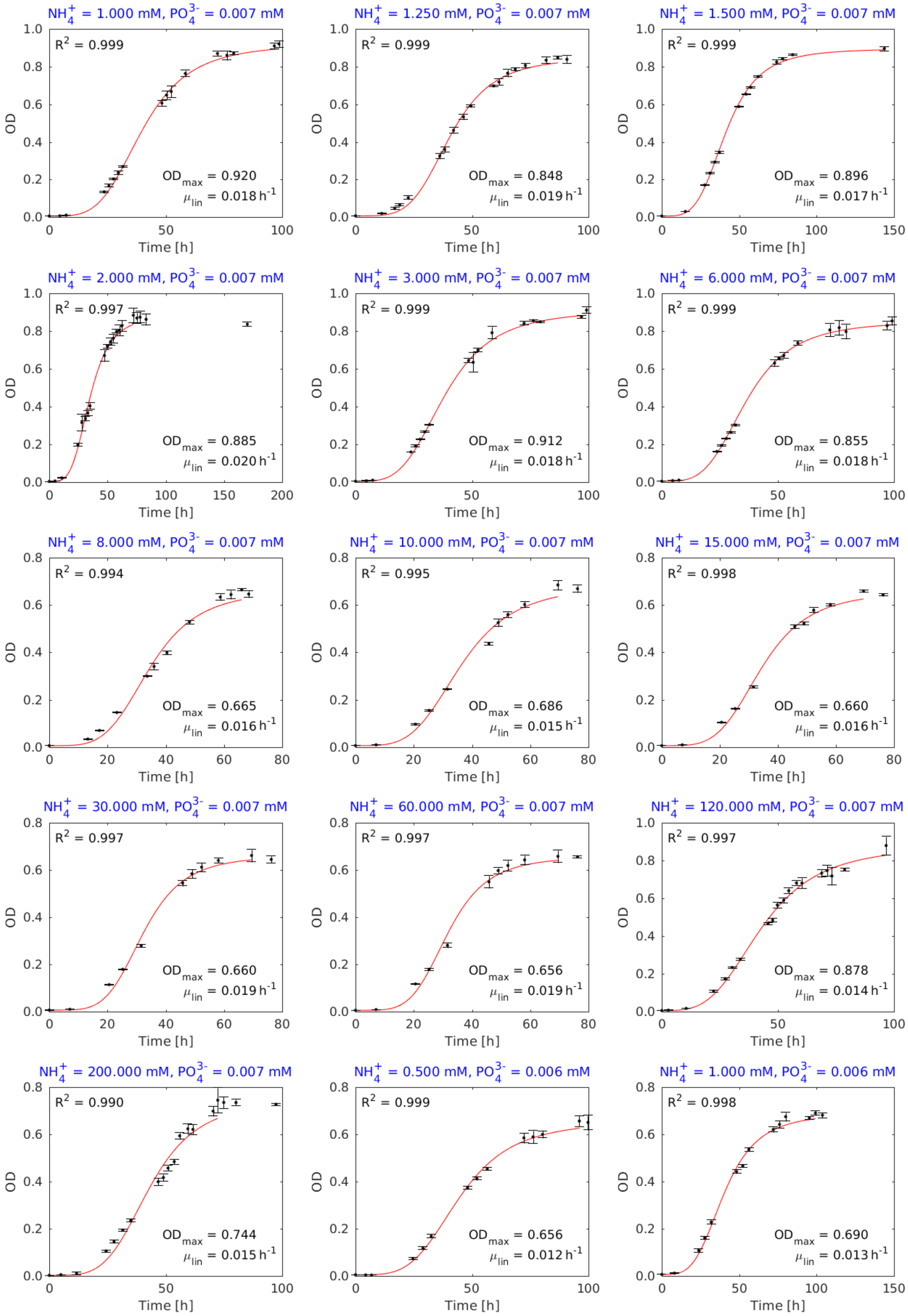
NH4+ = 1.000 mM, PO4 3- = 0.007 mM
R2 = 0.999
ODmax = 0.920
μlin = 0.018 h-1
NH4+ = 1.250 mM, PO4 3- = 0.007 mM
R2 = 0.999
ODmax = 0.848
μlin = 0.019 h-1
NH4+ = 1.500 mM, PO4 3- = 0.007 mM
R2 = 0.999
ODmax = 0.896
μlin = 0.017 h-1
NH4+ = 2.000 mM, PO4 3- = 0.007 mM
R2 = 0.997
ODmax = 0.885
μlin = 0.020 h-1
NH4+ = 3.000 mM, PO4 3- = 0.007 mM
R2 = 0.999
ODmax = 0.912
μlin = 0.018 h-1
NH4+ = 6.000 mM, PO4 3- = 0.007 mM
R2 = 0.999
ODmax = 0.855
μlin = 0.018 h-1
NH4+ = 8.000 mM, PO4 3- = 0.007 mM
R2 = 0.994
ODmax = 0.665
μlin = 0.016 h-1
NH4+ = 10.000 mM, PO4 3- = 0.007 mM
R2 = 0.995
ODmax = 0.686
μlin = 0.015 h-1
NH4+ = 15.000 mM, PO4 3- = 0.007 mM
R2 = 0.998
ODmax = 0.660
μlin = 0.016 h-1
NH4+ = 30.000 mM, PO4 3- = 0.007 mM
R2 = 0.997
ODmax = 0.660
μlin = 0.019 h-1
NH4+ = 60.000 mM, PO4 3- = 0.007 mM
R2 = 0.997
ODmax = 0.656
μlin = 0.019 h-1
NH4+ = 120.000 mM, PO4 3- = 0.007 mM
R2 = 0.997
ODmax = 0.878
μlin = 0.014 h-1
NH4+ = 200.000 mM, PO4 3- = 0.007 mM
R2 = 0.990
ODmax = 0.744
μlin = 0.015 h-1
NH4+ = 0.500 mM, PO4 3- = 0.006 mM
R2 = 0.999
ODmax = 0.656
μlin = 0.012 h-1
NH4+ = 1.000 mM, PO4 3- = 0.006 mM
R2 = 0.998
ODmax = 0.690
μlin = 0.013 h-1
OD
Time [h]

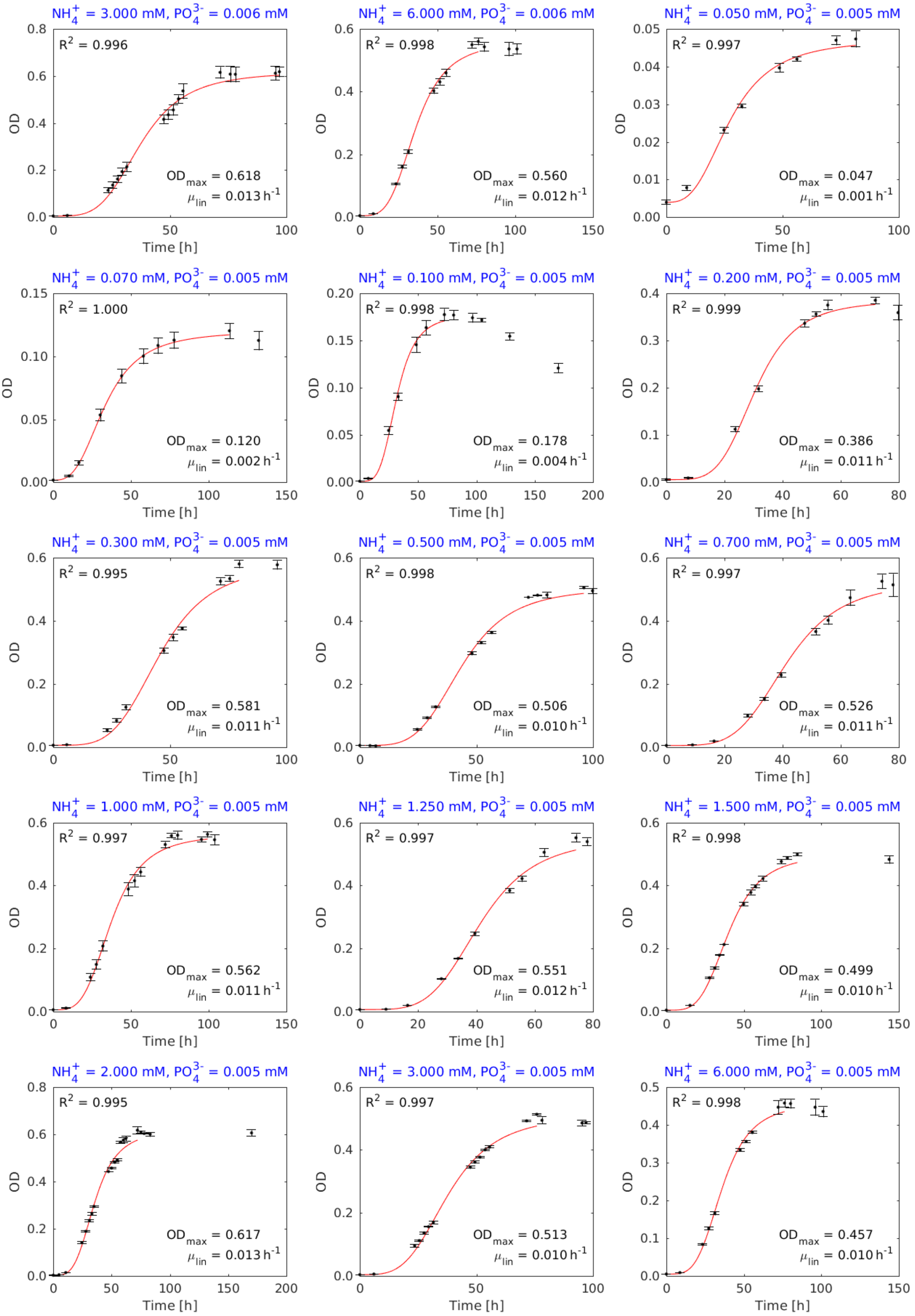

NH4+ = 3.000 mM, PO4 3- = 0.006 mM
R2 = 0.996
OD max = 0.618
μ lin = 0.013 h-1
NH4+ = 6.000 mM, PO4 3- = 0.006 mM
R2 = 0.998
OD max = 0.560
μ lin = 0.012 h-1
NH4+ = 0.050 mM, PO4 3- = 0.005 mM
R2 = 0.997
OD max = 0.047
μ lin = 0.001 h-1
NH4+ = 0.070 mM, PO4 3- = 0.005 mM
R2 = 1.000
OD max = 0.120
μ lin = 0.002 h-1
NH4+ = 0.100 mM, PO4 3- = 0.005 mM
R2 = 0.998
OD max = 0.178
μ lin = 0.004 h-1
NH4+ = 0.200 mM, PO4 3- = 0.005 mM
R2 = 0.999
OD max = 0.386
μ lin = 0.011 h-1
NH4+ = 0.300 mM, PO4 3- = 0.005 mM
R2 = 0.995
OD max = 0.581
μ lin = 0.011 h-1
NH4+ = 0.500 mM, PO4 3- = 0.005 mM
R2 = 0.998
OD max = 0.506
μ lin = 0.010 h-1
NH4+ = 0.700 mM, PO4 3- = 0.005 mM
R2 = 0.997
OD max = 0.526
μ lin = 0.011 h-1
NH4+ = 1.000 mM, PO4 3- = 0.005 mM
R2 = 0.997
OD max = 0.562
μ lin = 0.011 h-1
NH4+ = 1.250 mM, PO4 3- = 0.005 mM
R2 = 0.997
OD max = 0.551
μ lin = 0.012 h-1
NH4+ = 1.500 mM, PO4 3- = 0.005 mM
R2 = 0.998
OD max = 0.499
μ lin = 0.010 h-1
NH4+ = 2.000 mM, PO4 3- = 0.005 mM
R2 = 0.995
OD max = 0.617
μ lin = 0.013 h-1
NH4+ = 3.000 mM, PO4 3- = 0.005 mM
R2 = 0.997
OD max = 0.513
μ lin = 0.010 h-1
NH4+ = 6.000 mM, PO4 3- = 0.005 mM
R2 = 0.998
OD max = 0.457
μ lin = 0.010 h-1
OD
Time [h]

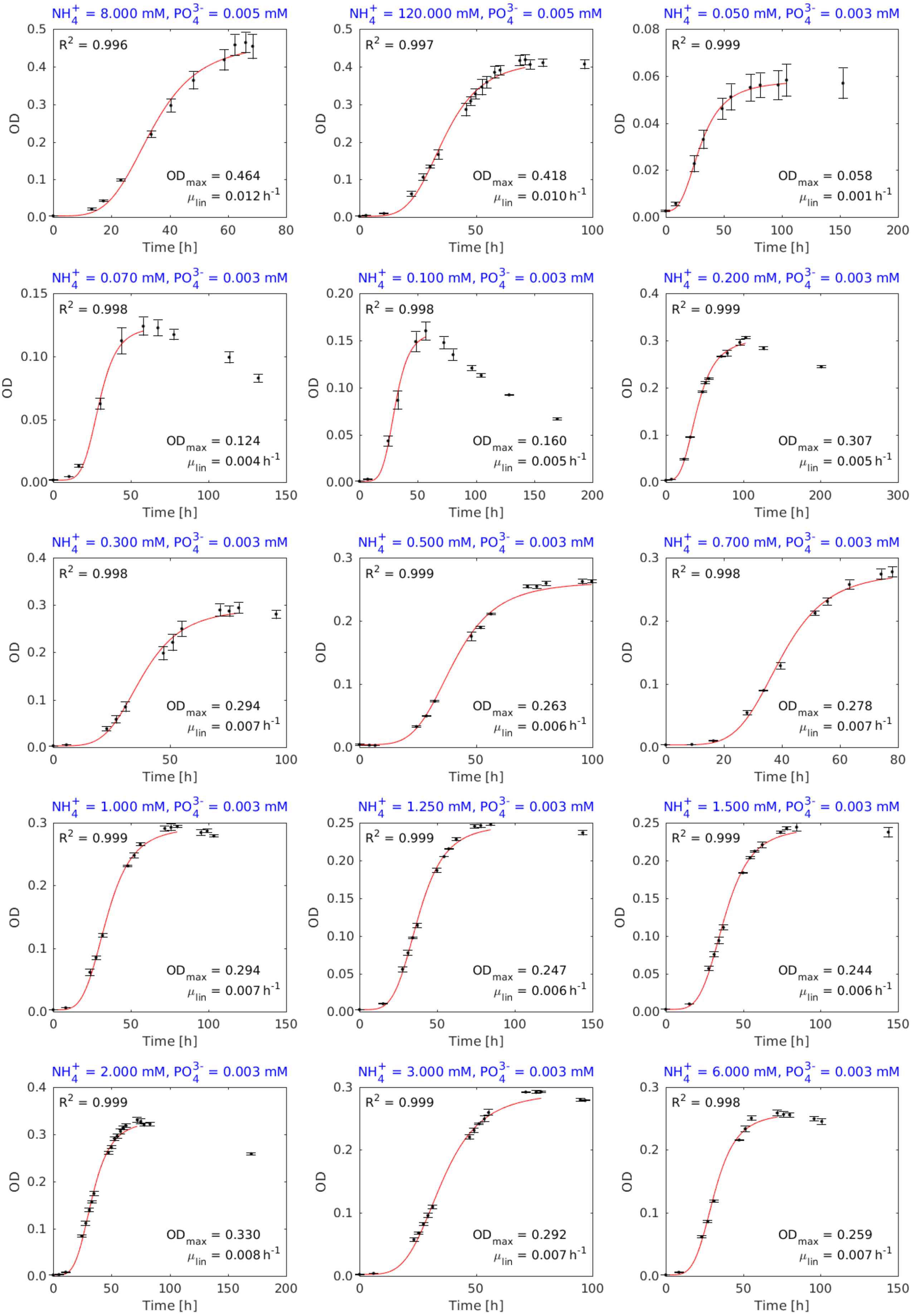

NH4+ = 8.000 mM, PO43- = 0.005 mM
R2 = 0.996
ODmax = 0.464
μlin = 0.012 h-1
NH4+ = 120.000 mM, PO43- = 0.005 mM
R2 = 0.997
ODmax = 0.418
μlin = 0.010 h-1
NH4+ = 0.050 mM, PO43- = 0.003 mM
R2 = 0.999
ODmax = 0.058
μlin = 0.001 h-1
NH4+ = 0.070 mM, PO43- = 0.003 mM
R2 = 0.998
ODmax = 0.124
μlin = 0.004 h-1
NH4+ = 0.100 mM, PO43- = 0.003 mM
R2 = 0.998
ODmax = 0.160
μlin = 0.005 h-1
NH4+ = 0.200 mM, PO43- = 0.003 mM
R2 = 0.999
ODmax = 0.307
μlin = 0.005 h-1
NH4+ = 0.300 mM, PO43- = 0.003 mM
R2 = 0.998
ODmax = 0.294
μlin = 0.007 h-1
NH4+ = 0.500 mM, PO43- = 0.003 mM
R2 = 0.999
ODmax = 0.263
μlin = 0.006 h-1
NH4+ = 0.700 mM, PO43- = 0.003 mM
R2 = 0.998
ODmax = 0.278
μlin = 0.007 h-1
NH4+ = 1.000 mM, PO43- = 0.003 mM
R2 = 0.999
ODmax = 0.294
μlin = 0.007 h-1
NH4+ = 1.250 mM, PO43- = 0.003 mM
R2 = 0.999
ODmax = 0.247
μlin = 0.006 h-1
NH4+ = 1.500 mM, PO43- = 0.003 mM
R2 = 0.999
ODmax = 0.244
μlin = 0.006 h-1
NH4+ = 2.000 mM, PO43- = 0.003 mM
R2 = 0.999
ODmax = 0.330
μlin = 0.008 h-1
NH4+ = 3.000 mM, PO43- = 0.003 mM
R2 = 0.999
ODmax = 0.292
μlin = 0.007 h-1
NH4+ = 6.000 mM, PO43- = 0.003 mM
R2 = 0.998
ODmax = 0.259
μlin = 0.007 h-1
OD
Time [h]

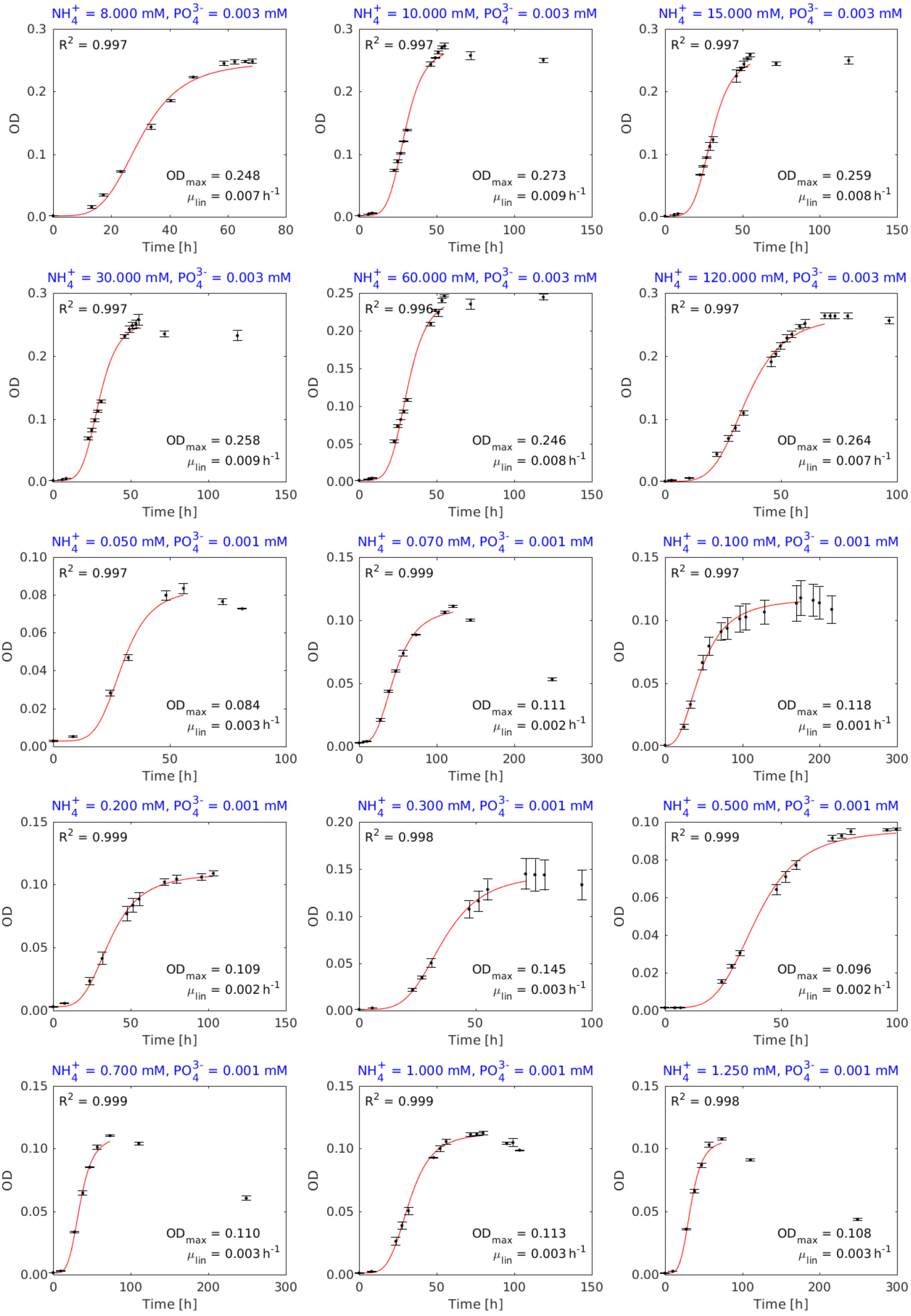
NH4+ = 8.000 mM, PO43- = 0.003 mM
R2 = 0.997
ODmax = 0.248
μlin = 0.007 h-1
NH4+ = 10.000 mM, PO43- = 0.003 mM
R2 = 0.997
ODmax = 0.273
μlin = 0.009 h-1
NH4+ = 15.000 mM, PO43- = 0.003 mM
R2 = 0.997
ODmax = 0.259
μlin = 0.008 h-1
NH4+ = 30.000 mM, PO43- = 0.003 mM
R2 = 0.997
ODmax = 0.258
μlin = 0.009 h-1
NH4+ = 60.000 mM, PO43- = 0.003 mM
R2 = 0.996
ODmax = 0.246
μlin = 0.008 h-1
NH4+ = 120.000 mM, PO43- = 0.003 mM
R2 = 0.997
ODmax = 0.264
μlin = 0.007 h-1
NH4+ = 0.050 mM, PO43- = 0.001 mM
R2 = 0.997
ODmax = 0.084
μlin = 0.003 h-1
NH4+ = 0.070 mM, PO43- = 0.001 mM
R2 = 0.999
ODmax = 0.111
μlin = 0.002 h-1
NH4+ = 0.100 mM, PO43- = 0.001 mM
R2 = 0.997
ODmax = 0.118
μlin = 0.001 h-1
NH4+ = 0.200 mM, PO43- = 0.001 mM
R2 = 0.999
ODmax = 0.109
μlin = 0.002 h-1
NH4+ = 0.300 mM, PO43- = 0.001 mM
R2 = 0.998
ODmax = 0.145
μlin = 0.003 h-1
NH4+ = 0.500 mM, PO43- = 0.001 mM
R2 = 0.999
ODmax = 0.096
μlin = 0.002 h-1
NH4+ = 0.700 mM, PO43- = 0.001 mM
R2 = 0.999
ODmax = 0.110
μlin = 0.003 h-1
NH4+ = 1.000 mM, PO43- = 0.001 mM
R2 = 0.999
ODmax = 0.113
μlin = 0.003 h-1
NH4+ = 1.250 mM, PO43- = 0.001 mM
R2 = 0.998
ODmax = 0.108
μlin = 0.003 h-1
OD
Time [h]

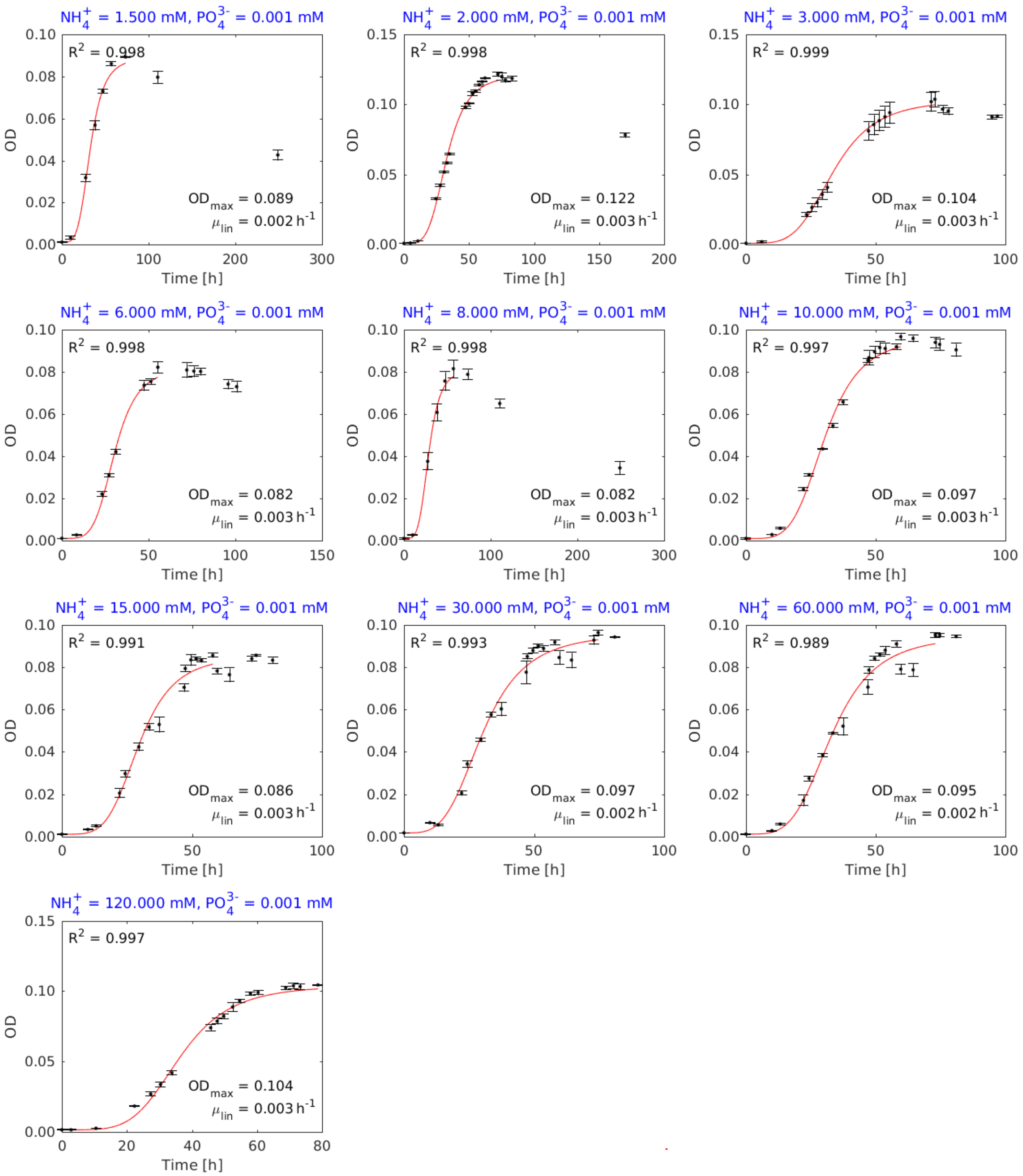

Figure S2 Compiled growth curves for *P. inhibens* DSM 17395 with fitted LDR function for 415 different NH_4^+ and PO_4^{3-} concentration pairs (data basis for Figs. 2–5). Each plot displays recorded experimental data for optical densities (OD) at 600 nm (black) and the fitted LDR function (red) with corresponding R^2 values, as well as the maximal OD (OD_{max}; at the transition into stationary growth phase) and the calculated maximal linear growth rate (μ_{lin}) achieved during the main active growth phase.

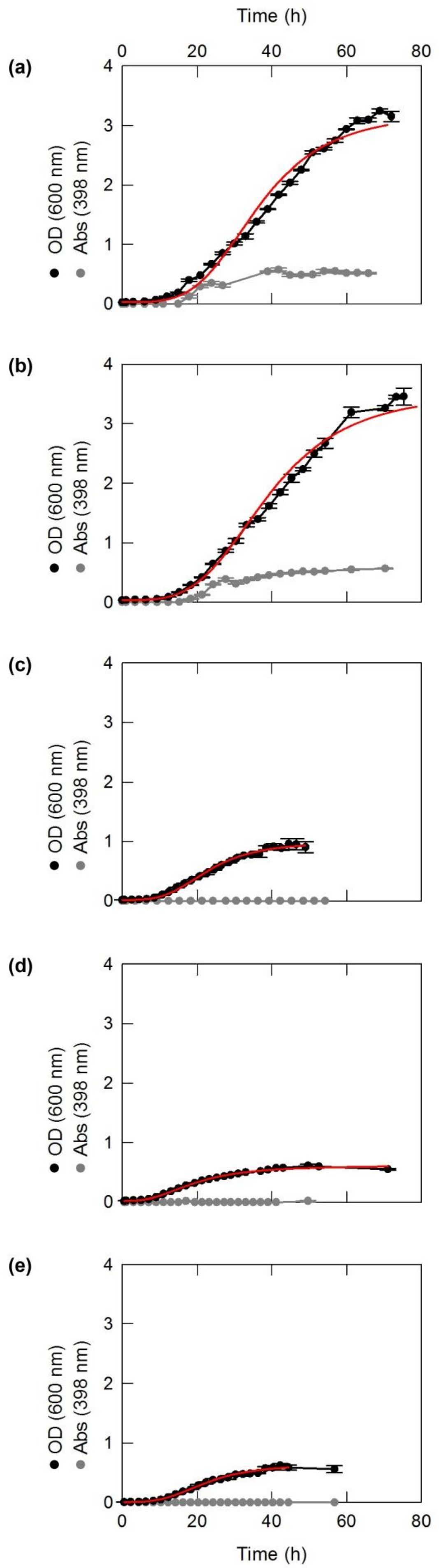

Figure S3 High-resolution growth curves of *P. inhibens* DSM 17395 with fitted LDR function (red line) for five selected NH_4^+ and PO_4^{3-} concentration pairs. Corresponding external N:P supply ratios were (values in brackets: NH_4^+ and PO_4^{3-} concentration pair): (**a**) 267 (8.0 mM, 30 µM), (**b**) 67 (2.0 mM, 30 µM), (**c**) 17 (0.5 mM, 30 µM), (**d**) 4 (0.5 mM, 125 µM) or (**e**) 1 (both 0.5 mM). An absorbance (Abs) increase at 398 nm indicates formation of the antibiotic tropodithietic acid (TDA).

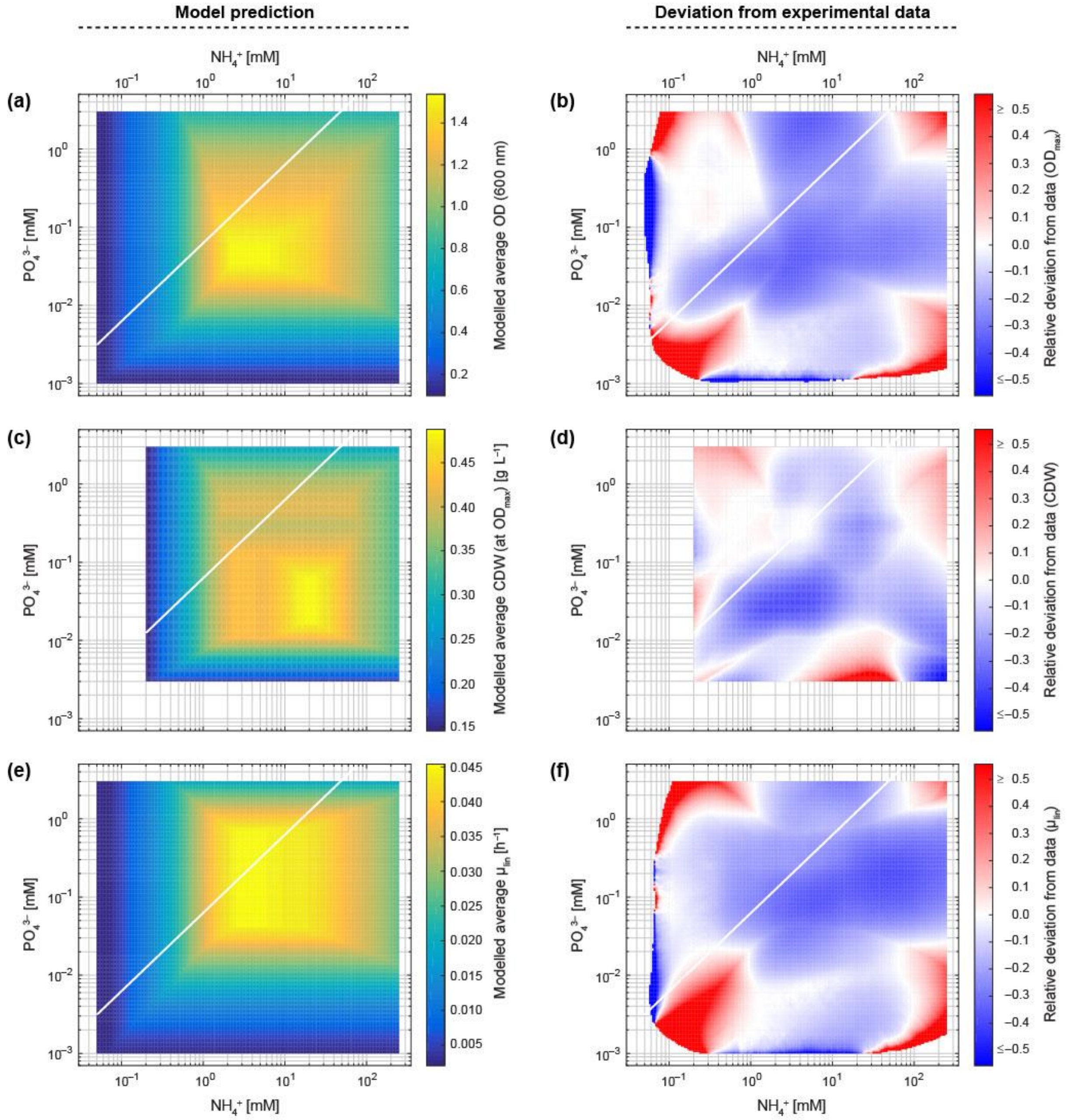

Figure S4 Comparison of experimental data with model prediction based on Liebig's law of the minimum. (**a, b**) maximal optical density (OD_{max}), (**c, d**) cellular dry weight (CDW) at OD_{max}, and (**e, f**) maximal linear growth rate (μ_{lin}) across the studied concentration range of NH_4^+ and PO_4^{3-}. (**a, c, e**) Values predicted by model based on Liebig's law of the minimum. (**b, d, f**) Relative deviation of model prediction from experimental values: red color, model prediction higher than experimental value; blue color, model prediction lower than experimental value; white, exact match between model prediction and experimental value.

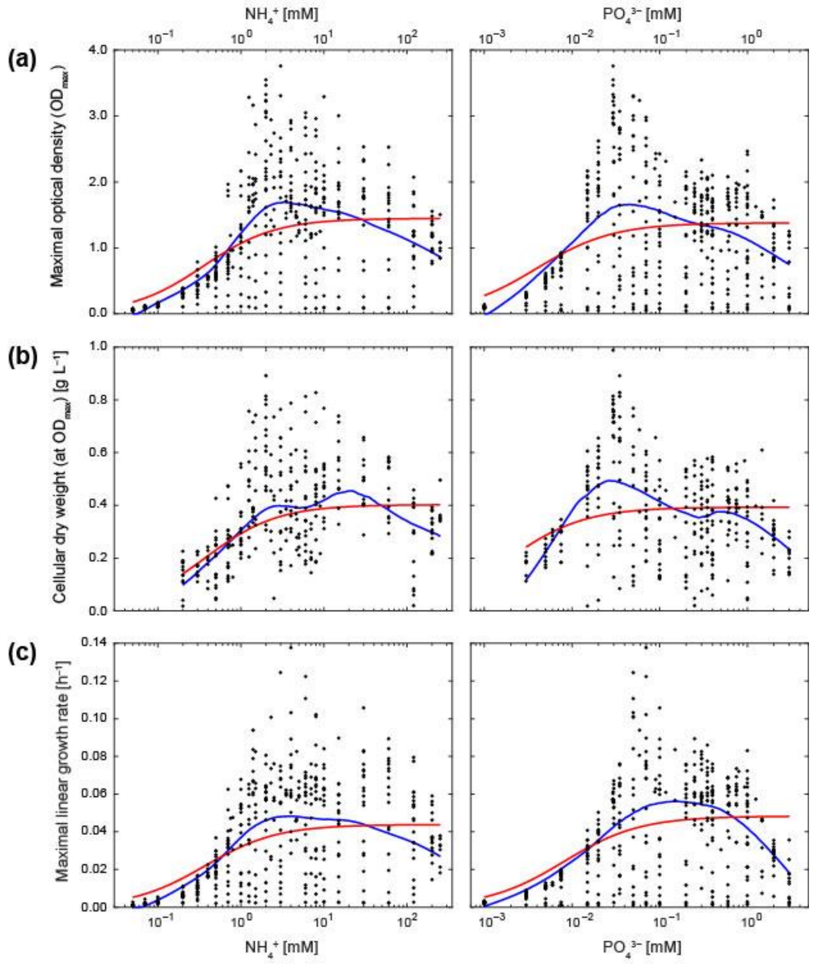

Figure S5 Experimental data (black dots) with fitted Monod function (red line). Semi-logarithmic profiles of (**a**) maximal optical density (OD_{max}), (**b**) cellular dry weight at OD_{max}, and (**c**) maximal linear growth rate (μ_{lin}) for *P. inhibens* DSM 17395 as a function of NH_4^+ or PO_4^{3-} concentration (see Fig. 2b–d for joint display in color maps). A Monod function was fitted to experimental data by minimizing the sum of squared errors. The blue line corresponds to the median derived from a 2D LOWESS fit to experimental data (see Fig. 3).
